# Cardiac forces regulate zebrafish heart valve delamination by modulating Nfat signaling

**DOI:** 10.1371/journal.pbio.3001505

**Published:** 2022-01-14

**Authors:** Renee Wei-Yan Chow, Hajime Fukui, Wei Xuan Chan, Kok Soon Justin Tan, Stéphane Roth, Anne-Laure Duchemin, Nadia Messaddeq, Hiroyuki Nakajima, Fei Liu, Nathalie Faggianelli-Conrozier, Andrey S. Klymchenko, Yap Choon Hwai, Naoki Mochizuki, Julien Vermot

**Affiliations:** 1 Institut de Génétique et de Biologie Moléculaire et Cellulaire (IGBMC), Centre National dela Recherche Scientifique UMR7104, Institut National de la Santé et de la Recherche Médicale U1258 and Université de Strasbourg, Illkirch, France; 2 Department of Cell Biology, National Cerebral and Cardiovascular Center Research Institute, Suita, Japan; 3 Department of Biomedical Engineering, National University of Singapore, Singapore; 4 Laboratoire de Bioimagerie et Pathologies, UMR 7021 CNRS, Université de Strasbourg, Faculté Rhin, Illkirch, France; 5 Department of Bioengineering, Imperial College London, London, United Kingdom; University of Pittsburgh, UNITED STATES

## Abstract

In the clinic, most cases of congenital heart valve defects are thought to arise through errors that occur after the endothelial–mesenchymal transition (EndoMT) stage of valve development. Although mechanical forces caused by heartbeat are essential modulators of cardiovascular development, their role in these later developmental events is poorly understood. To address this question, we used the zebrafish superior atrioventricular valve (AV) as a model. We found that cellularized cushions of the superior atrioventricular canal (AVC) morph into valve leaflets via mesenchymal–endothelial transition (MEndoT) and tissue sheet delamination. Defects in delamination result in thickened, hyperplastic valves, and reduced heart function. Mechanical, chemical, and genetic perturbation of cardiac forces showed that mechanical stimuli are important regulators of valve delamination. Mechanistically, we show that forces modulate Nfatc activity to control delamination. Together, our results establish the cellular and molecular signature of cardiac valve delamination in vivo and demonstrate the continuous regulatory role of mechanical forces and blood flow during valve formation.

## Abstract 2

Why do developing zebrafish atrioventricular heart valves become hyperplastic under certain hemodynamic conditions? This study suggests that part of the answer lies in how the mechanosensitive Nfat pathway regulates the valve mesenchymal-to-endothelial transition.

## Introduction

Heart valves are structures critical for ensuring unidirectional blood flow, and heart valve disease is a significant cause of illness and death worldwide. Given the intimate relationship between cardiac forces and cardiovascular development [1–5], a better understanding of how mechanical forces regulate heart valve morphogenesis can yield valuable insights into the origins of heart valve disease.

Heart valve development is initiated by the endothelial–mesenchymal transition (EndoMT) of a subset of endocardial cells, which migrate into the cardiac jelly (CJ) and subsequently proliferate [6]. Post-EndoMT, cellularized endocardial cushions remodel to form valve leaflets via various processes, including delamination, excavation, and elongation [7]. Cellular and molecular mechanisms underlying valve EndoMT and early endocardial cushion morphogenesis have been extensively studied [8–10], and the importance of mechanical forces in regulating EndoMT stages of valve development is becoming ever more appreciated [11–16]. By contrast, mechanisms underlying post-EndoMT valve morphogenesis remain poorly understood, and, except for a few studies [17], the role of cardiac forces in post-EndoMT processes remains unexplored. This is despite the fact that genetically modified mice with EndoMT defects rarely survive till birth, suggesting that most congenital valvuloseptal defects likely arise from errors that occur post-EndoMT [18].

The zebrafish is a valuable model to study the role of cardiac forces on valve development due to their optical accessibility and their ability to survive even with severe heart defects [19]. The current model of zebrafish atrioventricular valve (AV) formation postulates that ventricular and atrioventricular endocardial cells first undergo a partial EndoMT and migrate collectively into the CJ to form a bilayered structure [20–22]. The cellularized cushions then transform into free-moving valve leaflets via cellular rearrangement and elongation. Finally, the abluminal endocardial-derived valve interstitial cells (VICs) are joined by cells derived from the neural crest during valve maturation [23].

Here, we focus on the second step of zebrafish valve development and show that superior AV valve leaflets are first formed via delamination, where the tissue bilayer splits. By analyzing various EndoMT markers, we show that the extent of EndoMT is carefully controlled and that most abluminal cells of the bilayer undergo mesenchymal–endothelial transition (MEndoT) during delamination. By examining *gata1* mutants, where red blood cell formation is inhibited and wall shear stresses are low, we find that interfering with mechanical forces can cause defects in valve delamination that lead to hyperplastic and thickened superior AV valves. Finally, we show that the nuclear factor of activated T cells (Nfat) signaling pathway plays a critical role in the flow response during delamination stages. We propose a model whereby flow-dependent Nfat signaling in luminal endocardial cells of the atrioventricular canal (AVC) is required to inhibit *twist1b* expression in abluminal valve cells, thereby allowing these abluminal cells to undergo MEndoT and transform AV cellularized endocardial cushions into free-moving valve leaflets.

## Results

### Superior AV valves form via tissue sheet delamination

We first sought to better characterize how AVs first attain leaflet morphology. It has previously been suggested that imaging fixed hearts or hearts in which heartbeat have been stopped hearts can lead to misleading interpretations of valve morphology [24]. We thus assessed valve morphology at various stages between 48 and 98 hours postfertilization (hpf) by imaging both hearts that have been stopped using the drug 2,3-butanedione monoxime (BDM) and hearts that are beating normally. We focus on the superior AV valve as it forms earlier than the inferior AV valve [20,25] when the embryo is more transparent.

**Fig 1:**
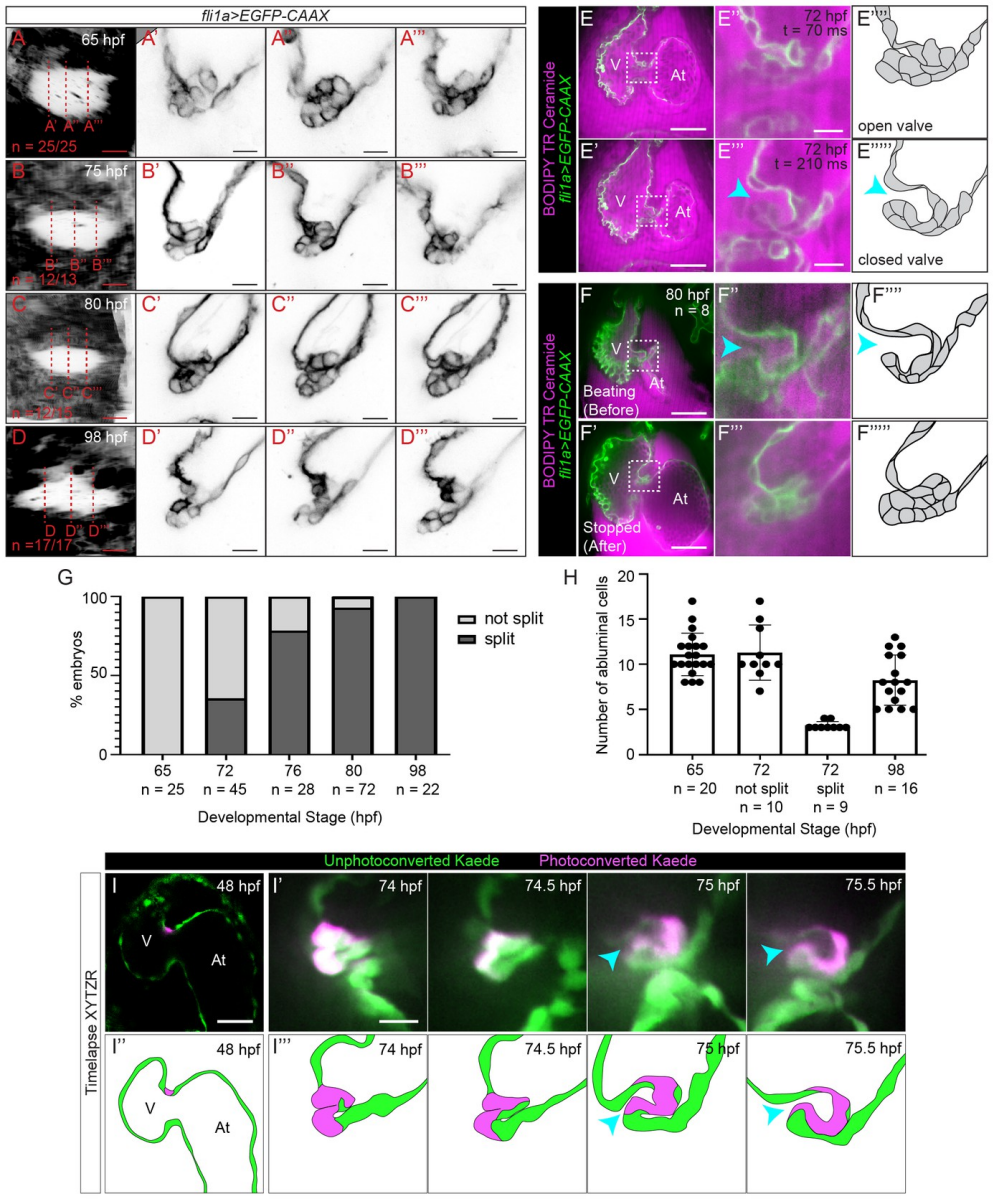
Imaging valves in beating hearts reveals a delamination step during superior AV valve morphogenesis. (A–C”’) Superior AV valve morphology as revealed using *Tg(fli1a*:*gal4ff;UAS*:*EGFP-CAAX)* at 65, 75, 80, and 98 hpf. (A, B, C, D) shows the AVC as seen when one looks from the ventricle through the AVC lumen into the atrium. The red dotted lines indicate image planes in which (A’–A”’), (B’–B”’), (C’–C”’), and (D’–D”’) are taken. (E–E”’) Selected frames from a movie of a beating heart where a superior AV valve leaflet has formed at 72 hpf. (E) shows the opened state of the valve leaflet during atrial contraction, while (E’) shows the closed state of the valve leaflet during atrial filling. (E”) and (E”’) are drawings showing our interpretations of valve morphology. (F–F””’) Example of an embryo that was imaged twice, first while the heart was beating (F,F”) and again once the heart has been stopped using BDM and tricaine (F’-F”’). (F”) and (F”’) correspond to the boxed regions in (F) and (F’), while (F””) and (F””’) are drawings showing our interpretations of valve morphology. (G) Graph showing percentage of developing superior AV valves that have split to form free-moving valve leaflets. (H) Graph showing number of abluminal cells at various developmental stages as determined using *Tg(fli1a*:*gal4ff;UAS*:*EGFP-CAAX)* embryos that have been immersed in BODIPY TR Ceramide. (I) Image of a *Tg(fli1a*:*Gal4ff;UAS*:*Kaede)* heart at 48 hpf, where a cell located in the AVC has been photoconverted. Scale bar: 50 μm. (I’) Images from a time-lapse movie of the embryo shown in (I) where images of the 3D beating heart were acquired every 30 minutes starting from 74 hpf. The images shown correspond to the point in the cardiac cycle when valve cells are the least compressed. A gap between the 2 cell layers is first observed at 75 hpf and the valve leaflet appears to be free moving by 75.5 hpf. Scale bar 10 μm. (I”,I”’) Interpretation of images shown in (I) and (I’). Cyan arrowheads point to the gap between the valve and the AVC wall. Note that the superior AV valve leaflet is in contact with the developing inferior AV valve in (E’), (E”’), (F), (F”), and the last 2 panels of (I’). The data underlying both graphs can be found in S1 Data. At, atrium; AV, atrioventricular valve; AVC, atrioventricular canal; BDM, 2,3-butanedione monoxime; hpf, hours postfertilization; V, ventricle.

Consistent with earlier studies [20–22], we found that cells from the ventricular side of the AVC send protrusions into the CJ as early as 48 to 50 hpf and appear to undergo collective cell migration into the CJ until 60 hpf (S1A–S1C Fig, S1 and S2 Movies). However, in contrast to a recent study [23], we find that cells do not appear to lose their packed morphology from 60 hpf. Rather, at 65 hpf, cells increase their organization such that, cells appear as though they are arranged in a rosette near the center of the valve (Fig 1A”). The valves remain highly organized at 75 and 80 hpf (Fig 1B–1B”’ and 1C–1C”’) and at 98 hpf, appear to be 2 cell layers thick except near the base of the valve and near the edges of the lumen (Fig 1D–1D”’). Surprisingly, using one of several transgenic lines that label endocardial cells (*Tg(fli1a*:*gal4ff;UAS*:*EGFP-CAAX)*, *Tg(fli1a*:*gal4ff;UAS*:*Kaede)*, and *Tg(fli1a*:*LifeAct-EGFP)*), we found that valve leaflets are formed as early as 72 hpf (Fig 1E–1E””’ and 1G, S3 Movie). To increase spatial resolution, we imaged *Tg(fli1a*:*gal4ff;UAS*:*EGFP-CAAX)* together with BODIPY-TR Ceramide, a red fluorescent dye, which counterstains the blood plasma and CJ. This allowed us to determine valve morphology at cellular resolution and perform cell counting. Approximately 11 cells migrate into the CJ by 65 hpf. At 72 hpf, valve leaflets were 2 cell layers thick except at the base of the leaflet, where 3 to 4 cells remain in the CJ and form the abluminal hinge cells of the leaflet (Fig 1E–1E””’ and 1H). Until 80 hpf, we find that valve leaflets tend to fold in stopped hearts such that the inner layer of valve endocardial cells is in contact with endocardial cells of the AVC wall (Fig 1F–1F””’, S2A–A””’ and S2B–S2B””’ Fig).

It has been proposed that valves transform from primitive structures into valve leaflets via the gradual process of elongation, involving abluminal cell rearrangement/VIC invasion, cell proliferation, and the thinning of luminal valve cells [23]. Given the tissue morphologies we observed, we hypothesized that primitive structures instead transition to leaflet morphology via tissue sheet delamination, where the bilayer of cells that have migrated into the CJ splits into 2 separate layers. The fact that we only see 2 main phenotypes—cellular cushions and valve leaflets—between 72 and 80 hpf suggests that this is a very fast process. We verified that delamination occurs and that it can take place within an hour via time-lapse imaging (3D + cardiac cycle + developmental time) of the beating heart (Fig 1I–1I”, S2C–C’ Fig, S4 and S5 Movies).

### Abluminal hinge cells give rise to VICs

**Fig 2:**
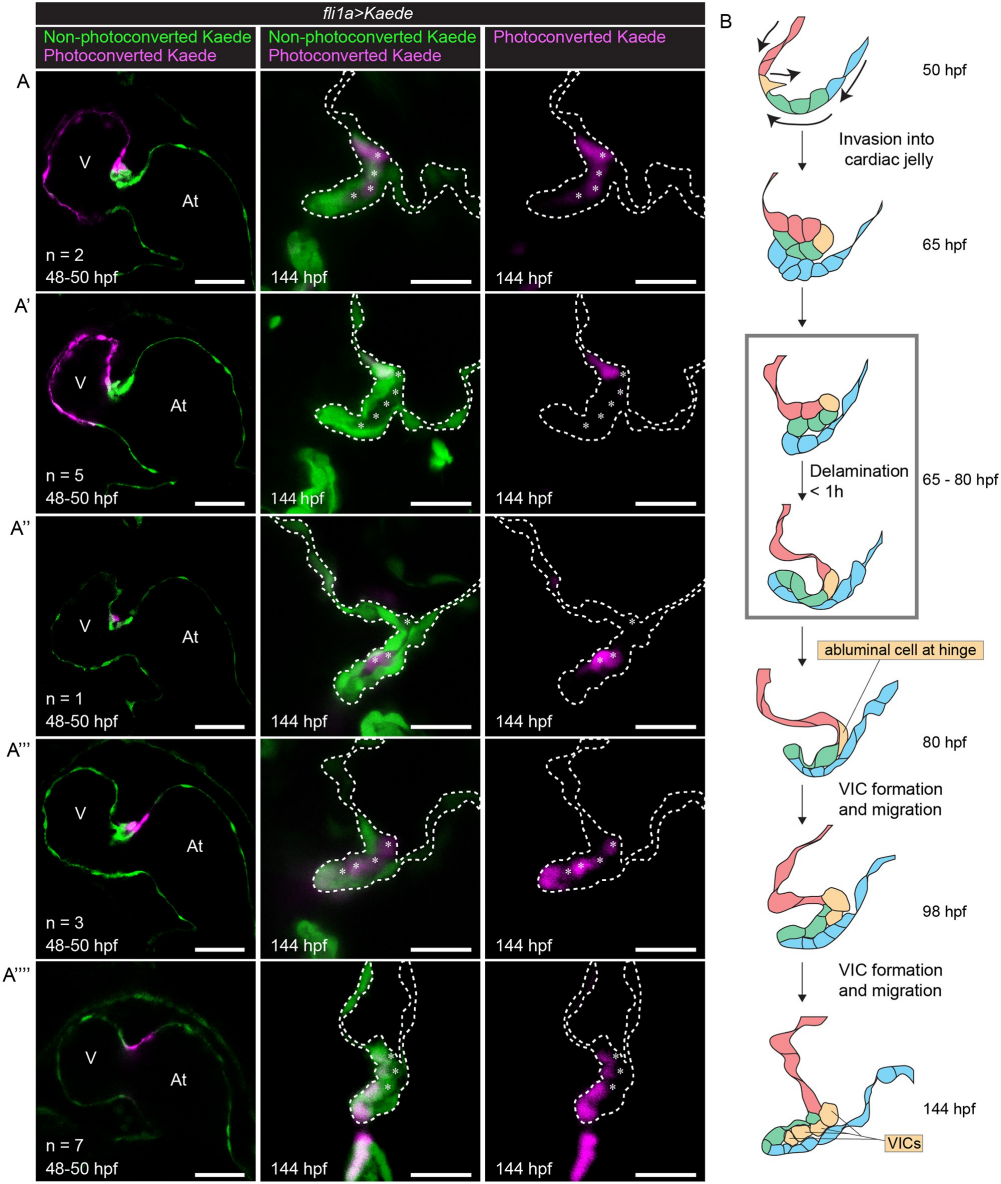
VICs at 144 hpf are derived from abluminal hinge cells at 80 hpf. (A–A””) *Tg(fli1a*:*gal4ff;UAS*:*Kaede)* embryonic hearts stopped using BDM and photoconverted at 48 to 50 hpf (left column). Embryos were then returned to normal media and allowed to grow normally until 144 hpf, when the hearts were stopped using BDM again and imaged (middle and right columns). (A) Embryos where the ventricle and the cell at the ventricular side of the AVC is photoconverted. Photoconverted cells can be seen between the 2 luminal layers of the valve at 144 hpf. (Ventricular cells appear to have degraded photoconverted Kaede.) (A’) Embryos where the ventricle is photoconverted. Photoconverted cells can be seen at the luminal side of the valve base at 144 hpf. (Ventricular cells appear to have degraded photoconverted Kaede.) (A”) Embryo where one cell migrating in is photoconverted. Photoconverted cells can be seen between the 2 luminal layers of the valve at 144 hpf. (A”’) Embryos where the deepest cell inside the CJ and the atrial edge of the AVC are photoconverted. At 144 hpf, photoconverted cells can be seen between the 2 luminal layers of the valve, presumably derived from the cell that was deepest inside the CJ at 48 to 50 hpf. Luminal photoconverted cells can also be seen at the tip of the valve, presumably derived from the atrial side of the AVC at 48 to 50 hpf. (A””) Embryos where the atrial side of the AVC is photoconverted. Photoconverted cells can be found at the ventricular luminal layer of the valve leaflet. Asterisks label abluminal cells. Scale bar left column: 50 μm. Scale bar middle and right columns: 20 μm. (B) Model for zebrafish valve leaflet formation, cells are colored to indicate their position and fate over time. At 50 hpf, red cells represent ventricular endocardial cells. Yellow cells represent endocardial cells at the ventricular edge of the AVC. Green cells represent the remaining endocardial cells in the AVC. Blue cells represent atrial endocardial cells in the AVC. In subsequent stages, color schemes are kept to show the position and fate of cells over time. Cells derived from cells at 50 hpf due to cell proliferation are colored the same color as their mothers. Arrows in the top drawing indicate cell movements. Gray box marks period at which delamination can occur (65 to 80 hpf). Delamination itself takes place within 1 hour. At, atrium; AVC, atrioventricular canal; BDM, 2,3-butanedione monoxime; CJ, cardiac jelly; hpf, hours postfertilization; V, ventricle; VIC, valve interstitial cell.

Abluminal cells of the bilayer have been proposed to become future VICs [23,26]. We thus wondered if the few abluminal cells at the hinge of newly formed superior AV valve leaflets can give rise to the much larger population of endocardial-derived VICs seen at 98 hpf and 144 hpf. To address this, we performed photoconversion analysis [27], where we photoconverted valve progenitors at 48 hpf and imaged the beating heart at 80 hpf when most valves have gained leaflet morphology. As expected, we found that the endocardial cells leading the initial collective migration into the CJ form abluminal cells of the hinge, while cells that enter the CJ behind the leading cells form the AVC wall and the inner layer of the valve leaflet (S3A–S3A”’ Fig). We then repeated our photoconversion experiments but stopped hearts at 98 hpf (S3B Fig and 144 hpf (Fig 2A–2A””) observe the origin of VICs. We find that luminal cells of the valve leaflet remain luminal at later stages, suggesting that the few abluminal hinge cells proliferate at the hinge and their progeny migrates toward the distal part of the valve and give rise to all endocardial-derived VICs (S3C Fig, Fig 2B).

### Characterization of valve EndoMT/MEndoT in zebrafish

Valve delamination requires most abluminal cells of the bilayer to become luminal again, suggesting that these cells undergo MEndoT. We thus sought to examine the cellular signature of valve delamination by examining various endothelial and mesenchymal cell markers.

**Fig 3:**
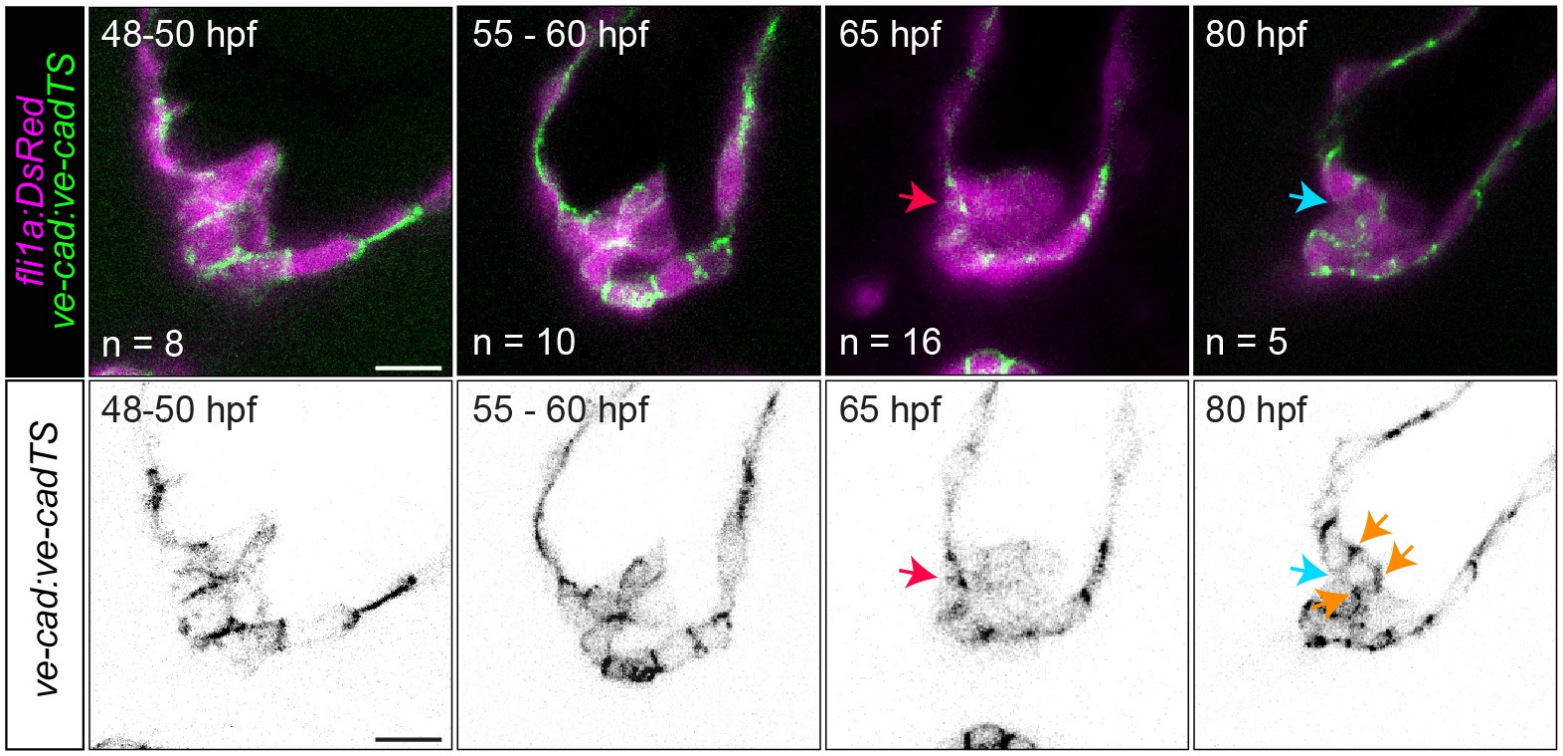
VE-cadherin is reexpressed during delamination stages. Images of *Tg(ve-cad*:*ve-cad-TS)* embryos at 48 to 50, 60, 65, and 80 hpf. Red arrow points to strong VE-cadherin signal between luminal cells at the future delamination site. Cyan arrow points to low VE-cadherin signal between the inner layer of the leaflet and the endocardial cells comprising the AVC wall. Orange arrows point to interfaces where VE-cadherin is up-regulated after being down-regulated following cell migration. Scale bar: 10 μm. AVC, atrioventricular canal; hpf, hours postfertilization.

We began by using immunohistochemistry to study the dynamics of VE-cadherin, the major endothelial adhesion molecule. [28] We found that VE-cadherin is expressed between all cell–cell interfaces between 48 and 55 hpf. At 65 hpf, VE-cadherin expression is down-regulated in abluminal cells. At 80 hpf, when most embryos have already formed valve leaflets, VE-cadherin is once again expressed between adjacent cells (S4A and S4B–B” Fig). At 120 hpf, VICs that have migrated further from the valve base appear to lose VE-cadherin expression (S4A Fig). To confirm that VE-cadherin is reexpressed at 80 hpf, we examined VE-cadherin expression using the transgenic line *Tg*(*ve-cad*:*ve-cadTS*) (Fig 3). The higher spatial resolution obtained here also reveals that VE-cadherin between luminal cells at the site of delamination is down-regulated between 65 and 80 hpf (Fig 3).

**Fig 4:**
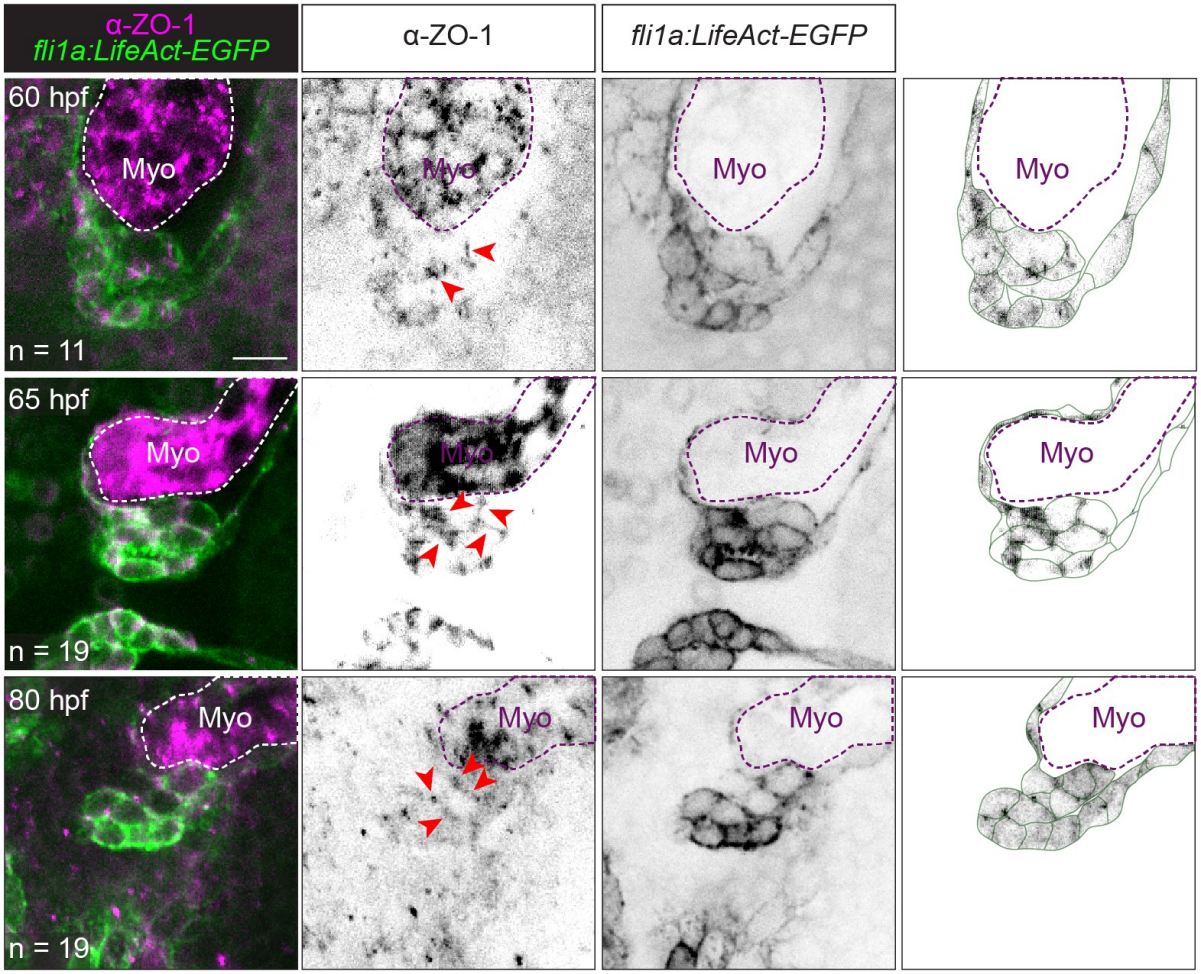
Tight junctions remain present during migration and delamination stages. Representative images of *Tg(fli1a*:*LifeAct-EGFP)* embryos immunostained with ZO-1 at 60, 65, and 80 hpf. The rightmost column shows the ZO-1 signal overlaid on top of an image showing our interpretation of valve cell morphology based on the EGFP signal. Dotted lines outline the Myo. Scale bar: 10 μm. hpf, hours postfertilization; Myo, myocardium; ZO-1, zonula occludens-1.

To examine tight junction dynamics, we performed immunostaining of zonula occludens-1 (ZO-1). We found that ZO-1 is expressed at cell–cell interfaces of all cells from 60 hpf through to 80 hpf (Fig 4). To confirm that tight junctions are present at 65 hpf when VE-cadherin is down-regulated in abluminal cells, some 65 hpf embryos were co-stained with the junctional protein Esama. We found that ZO-1 colocalized with Esama in abluminal cells (S5A Fig). Finally, we performed electron microscopy on 65 hpf *Tg(ve-cad*:*ve-cadTS)* embryos, further validating the presence of tight junctions when VE-cadherin is down-regulated (S5B–S5B”’ Fig).

Fibronectin deposition is considered a biomarker of EMT [29,30]. Previous studies have shown that fibronectin is expressed at the basal side of endocardial cells at 48 hpf [21] and surrounds early VICs at 120 hpf [23]. Using immunohistochemistry, we show that while fibronectin is consistently and strongly deposited along the basal side of AVC luminal cells from 48 hpf through to 98 hpf, the presence of fibronectin around other valve progenitors varies dynamically. At 60 and 65 hpf, fibronectin surrounds the cluster of migrating cells. At 80 hpf, when valves are expected to have delaminated, fibronectin can no longer be seen along the basal side of endocardial cells of the AVC wall. By 98 hpf, fibronectin is expressed strongly around abluminal cells (S6 Fig). This suggests that cells deposit fibronectin as they migrate into the CJ, but while some future luminal cells of the AVC wall degrade fibronectin, future abluminal cells begin to deposit new fibronectin into the CJ.

To examine whether valve cells lose and regain apical–basal polarity, we sought to examine the localization of podocalyxin, a CD34-related sialomucin protein that is commonly used as an apical cell surface marker in epithelial cells [31,32], including endothelial cells [33–35]. We thus generated a transgenic line where EGFP-tagged podocalyxin is expressed under the *fli1a* promoter, *Tg(fli1a*:*EGFP-Podxl)*, and crossed it with *Tg(fli1a*:*myr-mCherry)*, a transgenic line that labels endothelial cell membranes with mCherry, to see how podocalyxin localization changes between 48 and 80 hpf (S7 Fig). By analyzing z-slices corresponding to the middle half of the valve, we found that podocalyxin tends to be apically localized in endocardial cells of the AVC at 48 to 50 hpf in both embryos where luminal cells are nonprotrusive (S7A–S7C’ and S7G–S7H’ Fig) and in embryos where some luminal cells are sending protrusions into the CJ (S7D–S7F’, S7I and S7K Fig). However, when we analyzed a smaller subset of cells corresponding to cells sending protrusions into the CJ and the few endocardial cells just adjacent to these cells at 48 to 50 hpf, we find that podocalyxin is not specifically apically localized, appearing instead to localize also in the cytoplasm, at the basal membrane, and at the cell–cell junctions (S7D–S7E’, S7J and S7K Fig). At 60 hpf, podocalyxin does not appear preferentially apically localized in abluminal cells (S7L–S7L” and S7O Fig). At 65 hpf, podocalyxin starts localizing at the future apical membrane of abluminal cells (S7M–S7M” and S7P Fig). At 80 hpf, podocalyxin is apically polarized in cells that were once abluminal (S7N–S7N” and S7Q Fig). These findings suggest that cells that migrate into the CJ lose apical–basal cell polarity and, except for hinge cells that remain in the CJ, regain it just prior to delamination.

**Fig 5:**
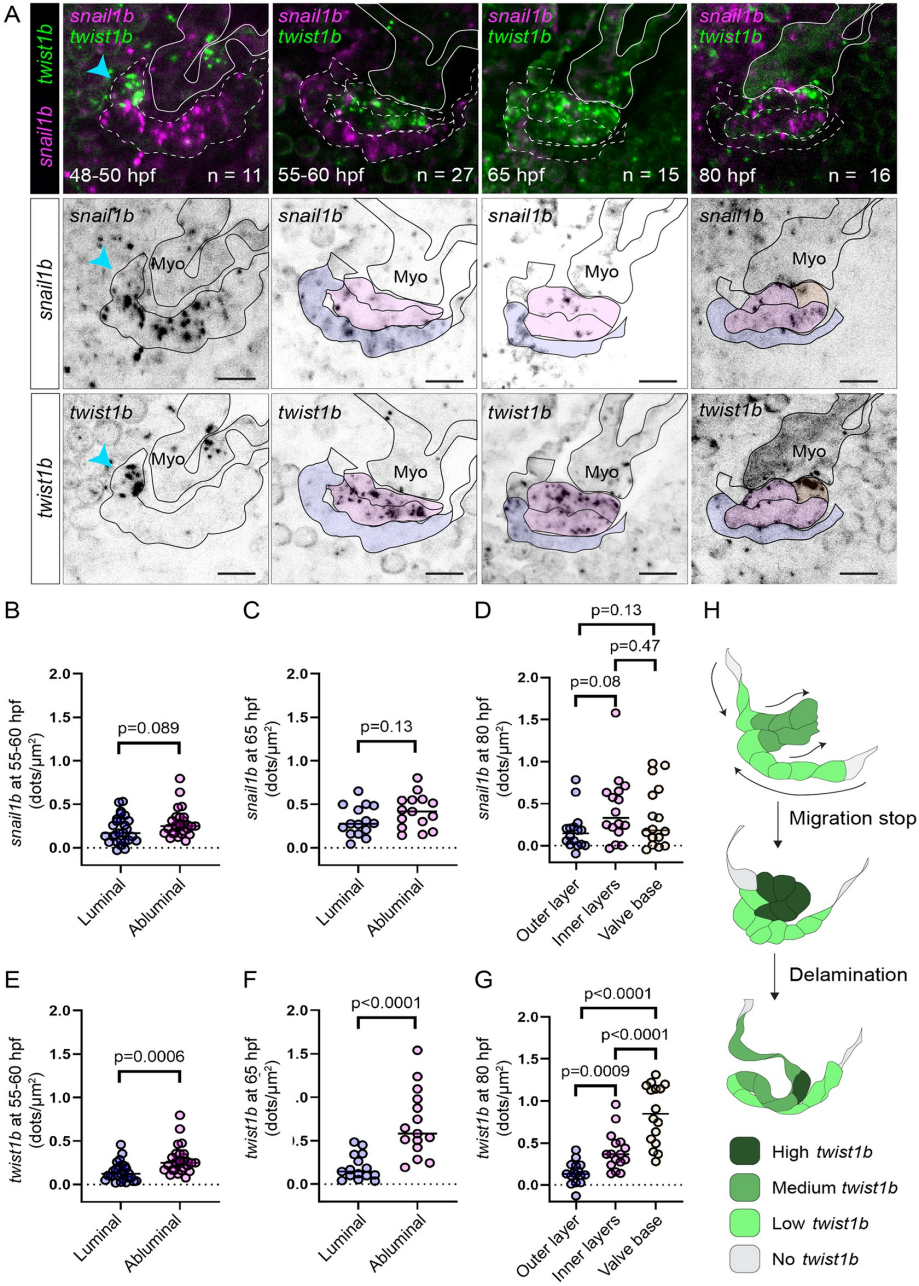
*Twist1b*, but not *snail1b*, is expressed highly specifically in abluminal cells. (A) Representative images of developing superior AV valves between 48 and 80 hpf that have been stained using *snail1b* and *twist1b* probes. The top row shows images where channels for *snail1b* (magenta) and *twist1b* (green) expression are shown simultaneously. The second and third rows show *snail1b* and *twist1b* signal, respectively, with our interpretation of valve morphology superimposed on the images. (B–D) and (E–G) are dot plots showing the number of detected dots, each corresponding to 1 *snail1b* (B–D) or 1 *twist1b* (E–G) mRNA molecule, calculated for different valve regions at 55 to 60, 65, and 80 hpf. The color of the dots corresponds to the regions shown in (A) and indicates where the measurement was performed. *p*-Values were determined using unpaired *t* test in (B, C, E, F) and using 1-way ANOVA in (D,G). (H) Suggested model showing the dynamics of *twist1b* expression. Scale bars: 10 μm. AV, atrioventricular valve; hpf, hours postfertilization.

We next analyzed the expression of the classical EMT markers *snail1/2* and *twist1* that are known to be important for heart valve formation in amniotes [36,37]. We examined the expression of *snail1b* and *twist1b* in hearts between 48 hpf and 80 hpf using RNAscope and found that both *snail1b* and *twist1b* are enriched at the AVC and outflow tract at all time points analyzed (Fig 5). Looking more closely at the superior AV valve region, *snail1b* is expressed in both luminal and abluminal endocardial cells to a similar extent (Fig 5A and 5B–5D). *Twist1b* is also expressed in both luminal and abluminal endocardial cells but shows greater differences in expression level depending on the valve region and is also expressed in the myocardial tongue. At 48 hpf, *twist1b* is expressed specifically in the region where we expect to find cells that are initiating migration into the CJ (Fig 5A), delimitating the cell pool that will eventually form the abluminal hinge cells of the valve. Between 55 and 65 hpf, *twist1b* is expressed at higher levels in abluminal cells (Fig 5A, 5E and 5F). At 80 hpf, when delamination is presumed to have occurred, *twist1b* expression is particularly high near the valve base, where we expect abluminal cells to reside (Fig 5A and 5G). As an alternative method to assess *twist1b* expression, we used the transgenic line *TgBAC(twist1b*:*GFP)* and imaged embryos at 75 hpf. Since GFP is stable, we were able to observe patterns of cumulative *twist1b* expression. In examining the AV valve forming region, we found that, consistent with our RNAscope analyses, *twist1b* is expressed in valve cells that have at an earlier point in development migrated into the CJ as well as cells of the myocardial tongue (S8 Fig).

Overall, we find that while cells transiently lose some endothelial characteristics/gain mesenchymal characteristics after migrating into the CJ, delamination comes with most cells regaining their endothelial characteristics. That ZO-1 remains expressed between these cells both during and after their migration into the CJ shows that cells do not become individualized, but rather stay in their highly organized layers as they remodel their adhesive contacts during delamination.

### Abnormal cardiac forces can block or delay valve delamination

Considering mechanical forces are key modulators of valve morphogenesis [5,38,39], we next sought to better understand the role of cardiac forces on valve cell behavior underlying tissue delamination. We first assessed whether valve delamination is dependent on normal flow by performing 2 types of flow manipulation: The first involves surgically inserting an approximately 30-micron diameter bead into the ventricle of the heart and thereby occluding blood flow (S9A Fig), and the second involves transferring embryos to media with 15 mM BDM, a myosin II inhibitor, to the water. Because prolonged perturbation of heartbeat and blood flow causes the heart to unloop and the chambers to collapse, we limited the length of intervention to 4 hours, performing the interventions at 72 hpf and analyzing the effect of the intervention at 76 hpf.

Inserting a bead into the ventricle predictably causes a drastic change in the flow profile (S9B Fig, S6 Movie), as well as a reduction in heartrate (S9C Fig). In imaging the beating heart, we found that inserting a bead into the ventricle completely stops the delamination process (S9D and S9E Fig). We confirm our results by immunostaining bead-inserted and sham embryos for VE-cadherin (S9F Fig) after imaging the beating heart, where we found that bead-inserted embryos fail to reexpress VE-cadherin (S9F and S9G Fig). BDM-treated embryos show reduced heartrate that is reversible upon returning the embryos to normal media for imaging (S9H Fig). Like bead insertion, BDM treatment completely stops the delamination process (S9I Fig).

Given that bead injection and BDM treatment causes severe changes to cardiac forces and complete abolition of valve delamination, we searched for ways to manipulate flow more subtly. We, therefore, examined *gata1* mutants where red blood formation is inhibited. A previous study approximating the heart as a linear tube has shown that the absence of red blood cells leads to a decrease in maximal wall shear stress of 2 to 3 times at 48 hpf [13]. Using a recently developed motion estimation algorithm [40], we were able to extract the position and motion of the cardiac wall (and blood cells in wild-type controls) in our movies of the beating heart and model wall shear stress in wild-type and *gata1* mutants at 65 hpf. At this stage, when heartbeat is faster and stronger, we found that maximal wall shear stress at the AVC is approximately 3 to 4 times lower in *gata1* mutants (S10 Fig).

*Gata1* mutant hearts appear normal at 48 hpf and, unlike *gata1* morphants, which have increased numbers of AVC endocardial cells [41], we found no differences in the number of AVC endocardial cells in *gata1* mutants compared to controls (S11A and S11B Fig). Searching for possible delamination defects, we found that we could screen out the majority of *gata1* mutants with early superior AV valve defects by removing embryos with pericardial edema at 65 hpf (S11C–S11H Fig). Imaging the beating heart of *gata1* mutants in the *Tg(fli1a*:*gal4ff;UAS*:*Kaede)* background at 80 hpf, we found that only 17% of *gata1* mutant superior AV valves manage to delaminate by 80 hpf compared to 92% of *gata1* control superior AV valves (S12A and S12B Fig, S7 and S8 Movies). Superior AV valves that have failed to delaminate by 80 hpf were imaged again at 98 hpf (S12A and S12B Fig, S8 and S9 Movies). In doing so, we found that for the majority of mutants, delamination is delayed rather than completely prevented. Taken together with the bead injection and BDM experiments, these results show that perturbation of cardiac forces can block or delay delamination.

### Abnormal hemodynamics causes delamination defects and thick valve phenotypes

**Fig 6:**
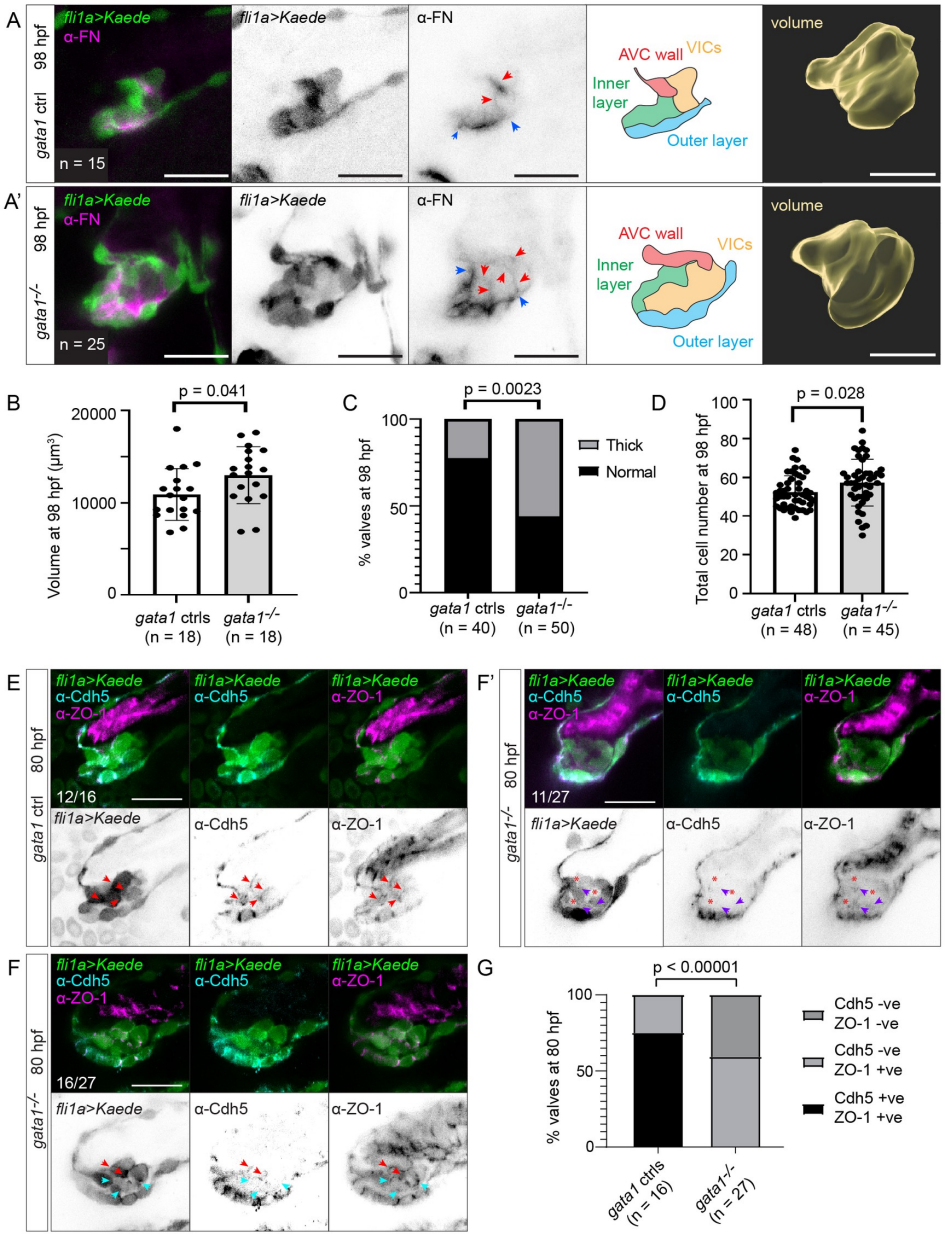
*Gata1* mutants have delamination defects and abnormally thick valves. (A) *Gata1* controls and *gata1* mutants in the *Tg(fli1a*:*gal4ff;UAS*:*Kaede)* background were analyzed at 98 hpf. Some of these were stained for FN (first to third column). Both control and mutant valves show FN immunoreactivity basal to the luminal layer of valve endocardial cells (between blue arrows), as well as around abluminal cells (red arrows). Schematics in the fourth column show how we demarcate different valve regions at 98 hpf. Fifth column shows 3D rendering of valve surface (inner layer, outer layer, and VICs). Scale bars: 20 μm. (B) Graph showing volume of valves at 98 hpf. *p*-Value: Welch *t* test. (C) Graph showing percentage of valves at 98 hpf that appear thick based on fixed embryos. *p*-Value: Fisher exact test. (D) Total number of cells in the valve region at 98 hpf (AVC wall, inner layer, outer layer, and VICs). *p*-Value: Welch *t* test. (E) *Gata1* controls immunostained for VE-cadherin and ZO-1 at 80 hpf. In 12/16 embryos, valve cells were immunopositive for both VE-cadherin and ZO-1 (red arrows). (F–F’) *Gata1* mutants immunostained for VE-cadherin and ZO-1 at 80 hpf. (F) In 16/27 embryos, some abluminal cells appear to have reexpressed VE-cadherin normally (red arrows), while others are immunopositive for ZO-1 but fail to reexpress VE-cadherin (cyan arrows). (F’) In 11/27 embryos, there is only residual ZO-1 signal in abluminal cells (purple arrows). In regions where ZO-1 signal is absent, gaps are formed between cells (red asterisks). (G) Graph showing quantification of Cdh5 and ZO-1 staining. *p*-Values are calculated using Fisher exact test for normal expression Cdd5 and ZO-1 expression pattern (Cdh5 +ve, ZO-1 +ve) versus abnormal expression Cdd5 and ZO-1 pattern (Cdh5 –ve, ZO-1 –ve; Cdh5 –ve, ZO-1 –ve). Scale bars: 20 μm. AVC, atrioventricular canal; FN, fibronectin; hpf, hours postfertilization; VIC, valve interstitial cell; ZO-1, zonula occludens-1.

In imaging the beating heart, we found that even delaminated *gata1* mutant superior AV valves appear thick (S12A Fig). We confirm that the majority of *gata1* mutant superior AV valves are thick by imaging stopped/fixed hearts at 98 hpf (Fig 6A and 6B). Segmenting *gata1* mutant valves showed that they are also enlarged (Fig 6A and 6C), and cell counting showed that they are hyperplastic (Fig 6D). By performing fibronectin immunostaining, we found that extra abluminal cells in *gata1* mutants express fibronectin, suggesting that these cells proceed to differentiate into VICs (Fig 6A, S10 and S11 Movies).

**Fig 7:**
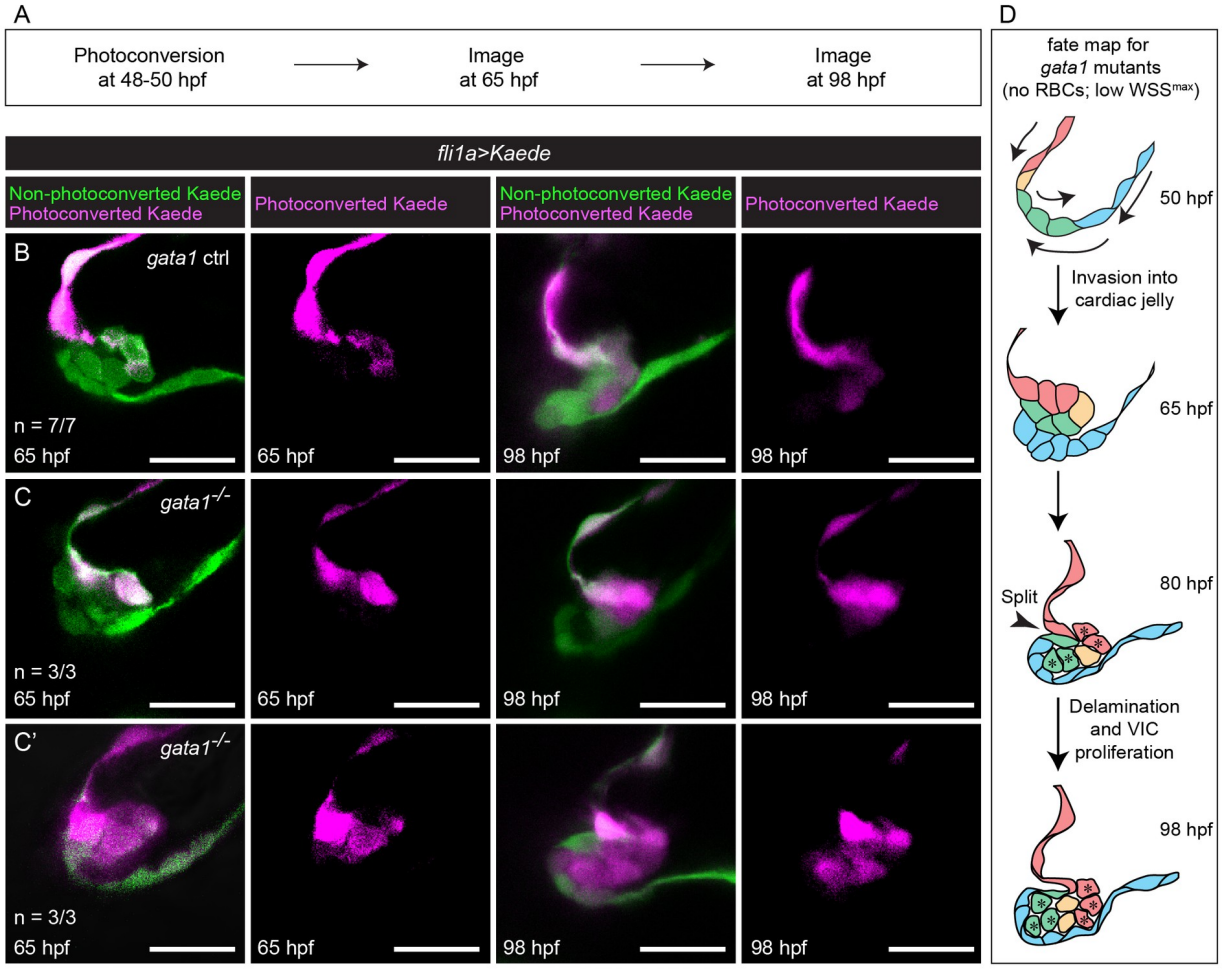
Photoconversion experiments show that delamination defects in *gata1* mutants result in misassignment of cells from luminal to abluminal cell fate. (A) Flow diagram summarizing the method used in this set of photoconversion experiments. At 48 to 50 hpf, embryonic hearts were stopped using BDM and the ventricle and the ventricular side of the AVC were photoconverted. The embryos were then returned to normal media and allowed to grow normally until 65 hpf, when the heart was stopped again using BDM and the valve imaged (first and second columns in (B–C’)). They were then returned to normal media and allowed to grow normally until 98 hpf, when the heart was stopped using BDM and they were imaged again (third and fourth columns in (B–C’)). (B) Representative images of *gata1* controls showing that if the superior layer of the abluminal bilayer and the cell deepest in the CJ is photoconverted at 65 hpf, then all abluminal cells will be photoconverted at 98 hpf. (C) Representative images of *gata1* mutants showing that if the superior layer of the abluminal bilayer and the cell deepest in the CJ is photoconverted at 65 hpf, then only some abluminal cells near the base of the valve will be photoconverted at 98 hpf. (C’) Representative images of *gata1* mutants, showing that if the entire abluminal bilayer is photoconverted at 65 hpf, then all abluminal cells are photoconverted at 98 hpf. (D) Proposed fate map of *gata1* mutants. Cells are colored to indicate position and fate over time. Asterisks at 80 hpf denote cells that have been misassigned from luminal to abluminal cell fate and their progeny. AVC, atrioventricular canal; BDM, 2,3-butanedione monoxime; CJ, cardiac jelly; hpf, hours postfertilization; RBC, red blood cell; VIC, valve interstitial cell; WSS, wall shear stress.

We hypothesized that the cellular origins of the thick valve phenotype could be due to abnormal delamination, where cells originally destined to become luminal cells end up remaining in the CJ. To test this, we immunostained 80 hpf *gata1* mutants for Cdh5 and ZO-1. We found that while 75% *gata1* controls reexpress Cdh5 and maintain expression of ZO-1, 100% of *gata1* mutants do not properly reexpress Cdh5 (Fig 6E, 6F–6F’ and 6G). In 41% of *gata1* mutants, ZO-1 signal appears diffuse or is absent around abluminal cells, which lose their packed cell morphology (Fig 6F’ and 6G). This supports our hypothesis as it suggests that abluminal bilayer cells in *gata1* mutants often fail to undergo MEndoT. We then performed photoconversion experiments, which confirmed that some valve cells in *gata1* mutants are incorrectly assigned to abluminal cell fate (Fig 7, S13 Fig).

**Fig 8:**
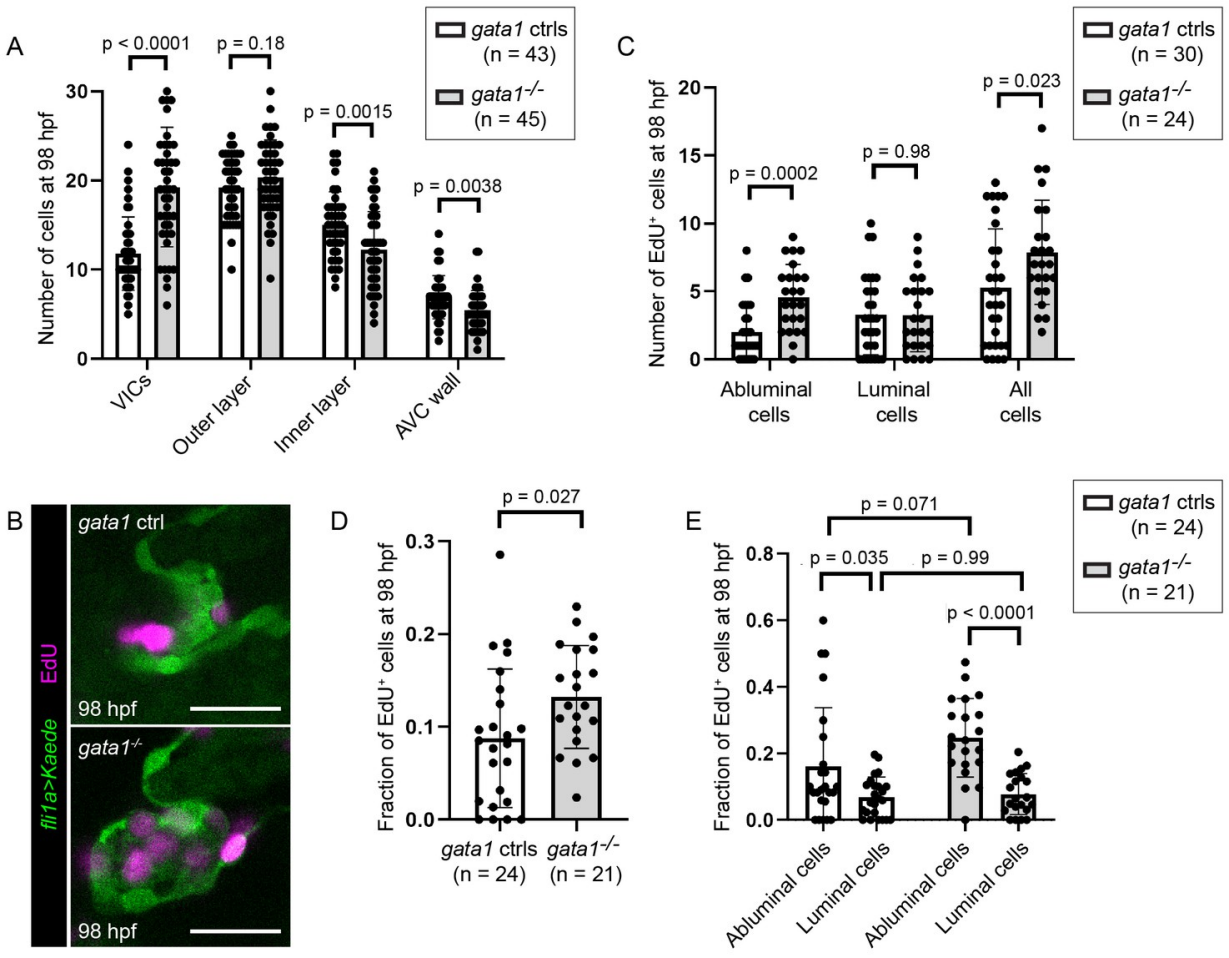
Delamination defects in *gata1* mutants leads to increased cell proliferation. (A) Number of cells in different valve regions at 98 hpf. *p*-Values based on unpaired *t* test. (B) Representative images of 98 hpf *gata1* control and mutant valves incubated with EdU from 74 to 98 hpf. Scale bars: 20 μm. (C) Graph showing number of EdU positive cells in 98 hpf *gata1* controls and mutant valves. *p*-Values are based on unpaired *t* tests. (D) Graph showing fraction valve cells that stained positive for EdU in 98 hpf *gata1* controls and mutant valves. *p*-Values are based on Welch *t* test. (E) Graph showing fraction of abluminal and luminal cells in 98 hpf *gata1* controls and mutant valves that stained positive for EdU. *p*-Values are based on 2-way ANOVA. AVC, atrioventricular canal; hpf, hours postfertilization; VIC, valve interstitial cell.

Misassignment of cell fate alone cannot completely account for hyperplasticity in the valve region in *gata1* mutants (Fig 6D). We thus wondered if cells misassigned from luminal to abluminal fate overproliferate. To test this, we first performed cell counting in 98 hpf embryos. We confirmed that while 98 hpf *gata1* mutant valves have decreased numbers of cells in the inner layer of the valve and the AVC wall, this decrease cannot account for all of the additional VICs (Fig 8A). We then performed EdU staining from 74 to 98 hpf to assess cell proliferation (Fig 8B). We found that *gata1* mutants valves have greater numbers of EdU^+^ abluminal cells (Fig 8C) and a greater fraction of valve cells that are EdU^+^ (Fig 8D). By measuring the fraction of EdU^+^ abluminal and luminal cells, we found that the difference in proliferation is not statistically different between abluminal cells in *gata1* mutants and controls (Fig 8E). However, abluminal cells were found to be more proliferative than luminal cells in both mutants and controls (Fig 8E). Together, these results suggest that cells misassigned from the inner layer of the valve/AVC wall to the valve interstitial space in *gata1* mutants become more proliferative, thereby exacerbating the overabundance of VICs and causing valve hyperplasticity (Fig 7D).

To test our hypothesis that wall shear stresses regulate valve delamination, we injected a nanoemulsion into the bloodstream of *gata1* mutants at 60 hpf (S14A Fig). This increased the blood viscosity of these mutants by approximately 17%, according to microfluidic rheometry measurements. We found that these nanoemulsion injections were able to partially rescue the *gata1* phenotype—while we did not see a significant decrease in the total number of thick valves in injected embryos (S14B and S14C Fig), injected embryos had significantly fewer abluminal cells compared uninjected embryos (S14B and S14D Fig). Altogether, these results suggest that abnormal wall shear stresses in *gata1* mutants could lead to delamination defects and thick, hyperplastic valves.

### Thick valves are associated with decreased valve function

We next sought to see if thick valves could decrease valve function. We find that all *gata1* mutants have pericardial edema by 98 hpf (S15A Fig), a phenotype commonly associated with poor heart function. However, *gata1* mutants at 80 hpf also have decreased heartrate (S15B Fig), making it difficult to isolate the role of valve morphology alone. This, as well as the difficulties in assessing flow profiles in *gata1* mutants, led us to examine embryos with mutations in the transcription factors *klf2a* and *2b*, 2 genes that are well known to have redundant roles in the endocardial cell response to cardiac forces [42]. It has been previously shown that at 96 hpf, about half of *klf2a* morphants have abnormally thick superior or inferior AV valves, while the other half have defects suggestive of failed EndoMT [21]. It is known that *klf2a klf2b* double mutants (henceforth referred to as simply *klf2* mutants in text) have increasing incidences of pericardial edema as they age from 2 dpf to 5 dpf [43]. Excluding approximately 50% of embryos that have pericardial edema at 65 hpf, we were able to screen out the majority of mutants with early cell migration defects in the superior AV valve (S15D Fig) and approximately 57% of remaining embryos have thick superior AV valves by 98 hpf (S15E and S15F Fig). We also confirmed that thick valves are more prevalent in *klf2a*^*+/−*^ *klf2b*^*−/−*^ mutants than their *klf2a*^*+/−*^ *klf2b*^*+/−*^ siblings (S15G Fig). Unlike *gata1* mutants, *klf2* mutants without pericardial edema at 65 hpf have normal heartrate at 80 hpf (S15C Fig), allowing us to examine the effect of thick valves on valve function.

We found that *klf2* mutants have lower survival rates compared to controls (S15H Fig). Brightfield imaging of the beating heart at 500 frames per second allowed us to visualize the movement of red blood cells over the cardiac cycle and demonstrated that both *klf2* mutants with thick superior AV valves and those with normal superior AV valves show increased reversing flow compared to wild-type controls, most likely because mutants with normal superior AV valves may still have abnormal inferior AV valves. However, the amount of reversing flow is greatest in mutants with thick superior AV valves (S15I, S15J–S15J”’, and S15K Fig, S12 and S13 Movies), thus suggesting that thick valves are associated with decreased valve function.

### Flow-dependent Nfatc signaling is required for proper valve delamination

We then proceeded to determine the mechanotransduction pathways involved in delamination. Since we have already confirmed that both *klf2a*^*−/−*^ *klf2b*^*−/−*^ and *klf2a*^*+/−*^ *klf2b*^*−/−*^ mutants have thick valve defects (S15E–S15G Fig), we wondered if down-regulation of these genes could be responsible for the delamination defects seen in the *gata1* mutant. However, while we found that *klf2* mutants have both delayed delamination and cell fate maps suggesting defects in delamination similar to that found in *gata1* mutants (S16A, S16B and S16F Fig), *klf2a* and *klf2b* appear up-regulated rather than down-regulated in *gata1* mutants (S16C and S16E Fig).

Searching elsewhere, we wondered if abnormal Nfat signaling could underlie the *gata1* mutant phenotype. In mice, Nfatc2/3/4 are expressed in the AVC myocardium, where it suppresses the expression of VEGF, thereby allowing the initiation of EndoMT in the AVC endocardium [44]. By contrast, Nfatc1 is expressed in the endocardium [44], and targeted deletion of *nfatc1* in the endocardium results in an overabundance of EndoMT-derived cushion mesenchyme, decreased cardiac neural crest-derived mesenchyme, and subsequent defects in valve remodeling [45]. In zebrafish, *nfatc1* is expressed in valve precursors in the endocardium [22,23], and endocardial Nfatc has recently been shown to be activated in blood flow-dependent manner [46].

**Fig 9:**
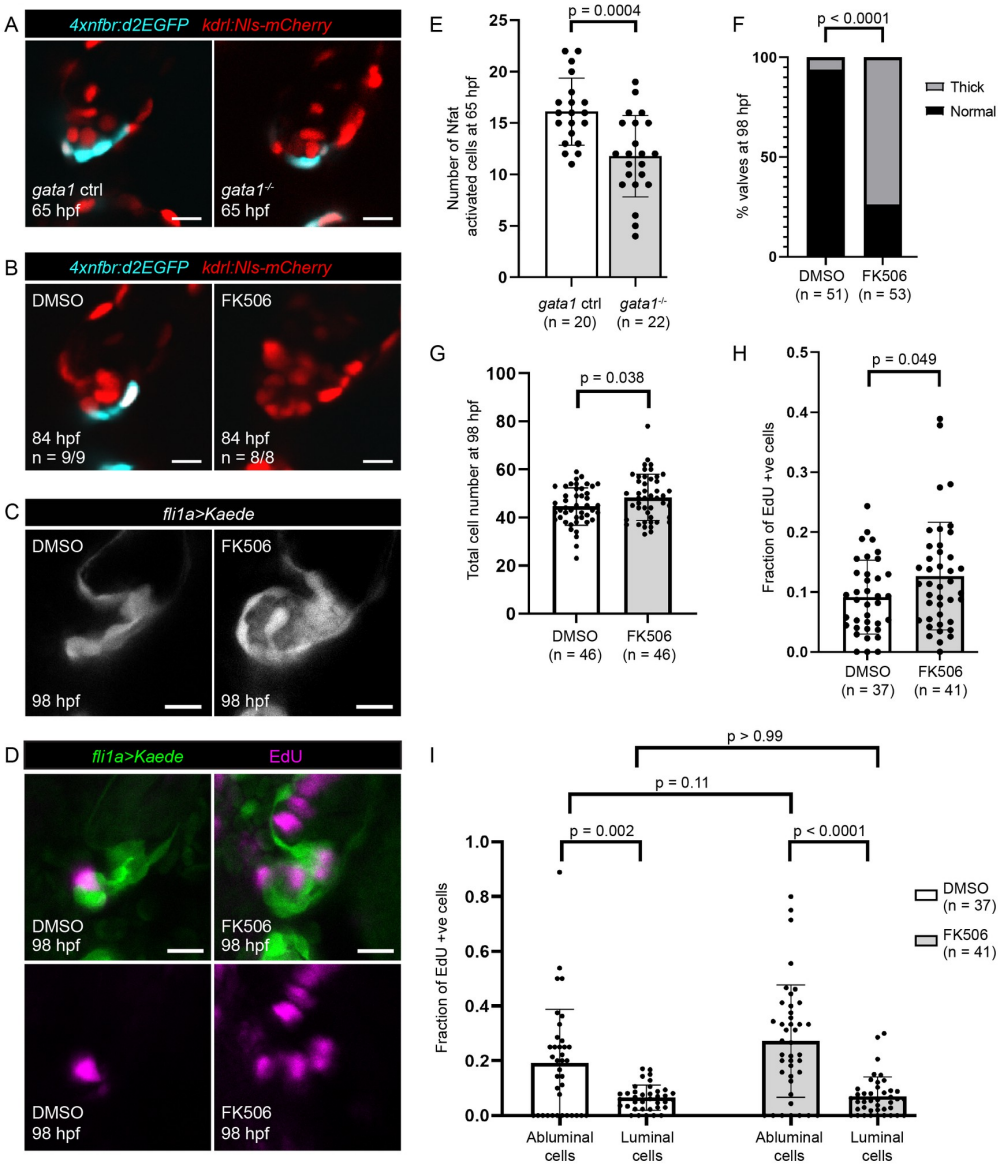
Inhibition of flow-dependent Nfatc signaling results in abnormally thick valves. (A) *Tg(4xnfbr*:*d2EGFP)* is a reporter line for Nfat activity. Representative images of *gata1* mutants and controls in the *Tg(4xnfbr*:*d2EGFP);Tg(kdrl*:*nls-mCherry)* background at 65hpf. (B) Representative images of valves in 84 hpf *Tg(4xnfbr*:*d2EGFP);Tg(kdrl*:*nls-mCherry)* embryos that have been treated with DMSO or FK506 from 60 hpf. (C) Representative images of 98 hpf valves in *Tg(fli1a*:*gal4;UAS*:*Kaede)* embryos treated with DMSO or FK506 from 60 to 98 hpf. (D) Representative images of embryos treated with DMSO or FK506 from 60 to 98 hpf and EdU from 74 to 98 hpf. (E) Graph showing number of Nfat activated cells in 65 hpf superior AV valves as determined using the *Tg(4xnfbr*:*d2EGFP);Tg(kdrl*:*nls-mCherry)* line. *p*-Values were calculated using unpaired *t* test. (F) Graph showing percentage of embryos with thick superior AV valves in embryos treated with DMSO and FK506 from 60 to 98 hpf. Statistical significance was calculated using Fisher exact test. (G) Graph showing number of cells in the superior AVC in embryos treated with DMSO and FK506 from 60 to 98 hpf. Statistical significance was calculated using Student *t* test. (H) Graph showing the fraction of EdU positive cells in the superior AVC in embryos treated with DMSO or FK506 from 60 to 98 hpf and EdU from 74 hpf to 98 hpf. Statistical significance determined by Student *t* test. (I) Graph showing the fraction of EdU positive luminal cells and EdU positive abluminal cells in embryos treated with DMSO or FK506 from 60 to 98 hpf and EdU from 74 hpf to 98 hpf. Statistical significance was determined by 2-way ANOVA. AV, atrioventricular valve; AVC, atrioventricular canal; hpf, hours postfertilization; Nfat, nuclear factor of activated T cells.

To see if flow forces could regulate delamination via Nfat signaling, we first sought to visualize Nfat activity in *gata1* mutants and controls. Using the Nfat binding element reporter line *Tg(4xnfbr*:*d2EGFP)*, which expresses d2EGFP in response to the binding of nuclear-localized Nfat protein [46], we found that Nfat is activated in the region corresponding to the outer layer of the future superior AV valve leaflet at 65 hpf and that the number of Nfat activated cells is decreased in *gata1* mutants compared to controls (Fig 9A). These results suggest that flow forces regulate Nfat activity in luminal endocardial cells at the stage when valves are preparing for delamination.

Next, we examined whether inhibition of Nfat signaling could lead to delays in delamination. To do so, we used FK506, an immunophilin ligand that down-regulates the mRNA and protein levels of the phosphatase calcineurin. Since calcineurin activity is necessary for the nuclear translocation of Nfatc proteins, FK506 inhibits Nfat activation [47–50]. Using the *Tg(4xnfbr*:*d2EGFP)* line, we confirmed that FK506 effectively suppresses Nfat activity in this system (Fig 9B). Then, to see if reduced Nfatc activity causes delays in delamination, we treated embryos with FK506 from 60 to 80 hpf and imaged the beating heart. We found that, unlike the *gata1* mutant, FK506-treated embryos do not show delays in delamination (S17A Fig).

**Fig 10:**
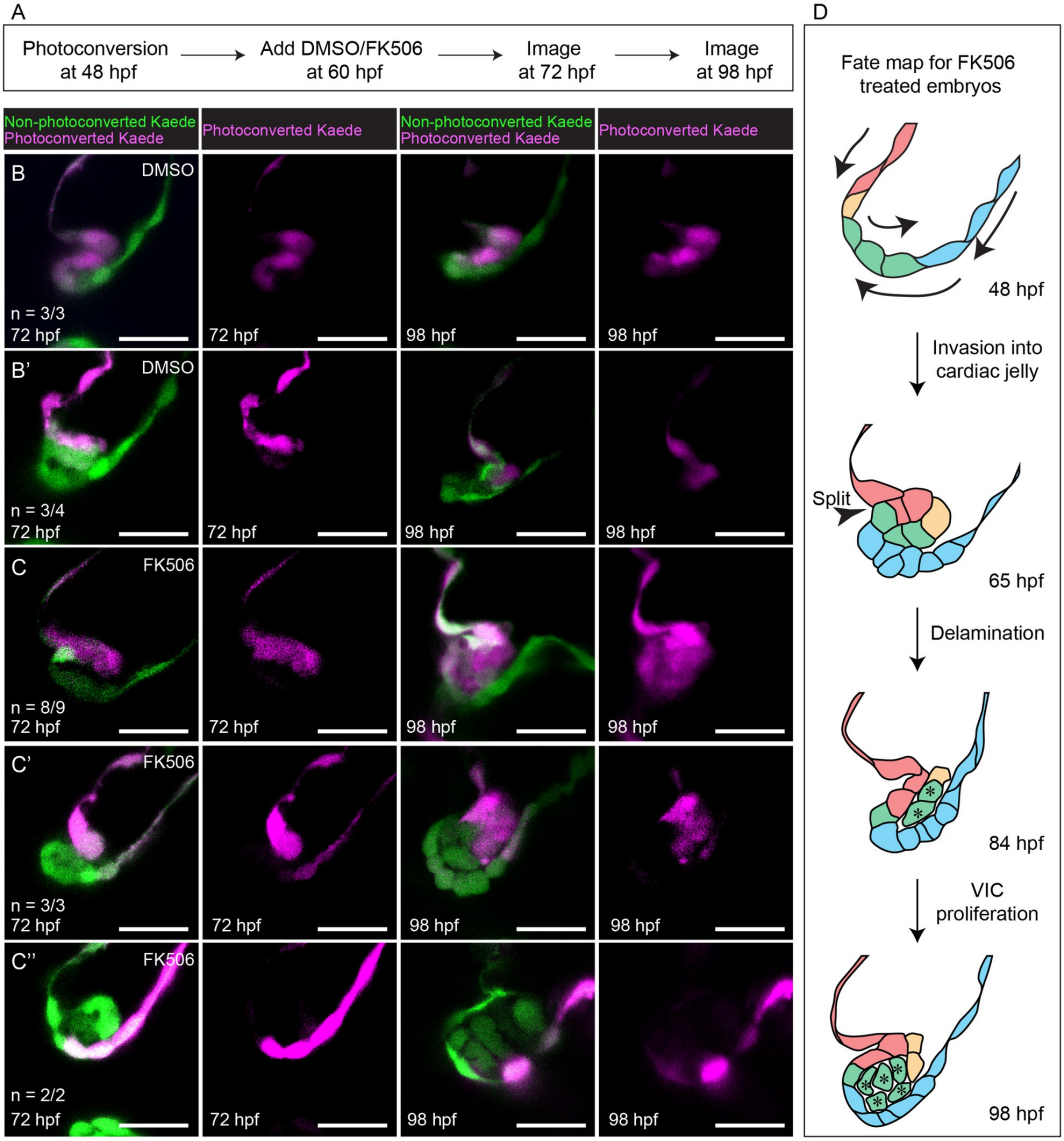
Photoconversion experiments show that delamination defects in FK506-treated embryos results in misassignment of cells from luminal to abluminal cell fate. (A) Flow diagram summarizing the method used in these photoconversion experiments. (B–B’) Representative images showing that DMSO treatment does not cause misassignment of cell fate. (C–C”) Representative images showing that FK506 treatment results in misassignment of cells from luminal to abluminal cell fate. Scale bars: 20 μm (D) Proposed fate map of FK506-treated embryos. Cells are colored to indicate position and fate over time. Asterisks denote cells that have been misassigned from luminal to abluminal cell fate and their progeny. hpf, hours postfertilization; VIC, valve interstitial cell.

We then sought to determine whether inhibition of Nfat signaling could cause delamination defects. To do so, we treated embryos with FK506 either from 60 to 98 hpf or from 60 to 80 hpf. We found that FK506 treatment resulted in thick, hyperplastic superior AV valves (Fig 9C, 9F and 9G, S17B and S17C Fig) and increased incidence of pericardial edema (S17D and S17E Fig). Then, to see if these thick valve phenotypes are caused by delamination defects, we performed VE-cadherin and ZO-1 immunostaining. We found that embryos treated with FK506 from 60 to 84 hpf results in an extra layer of VE-cadherin negative, ZO-1 positive cells situated between the 2 luminal layers of the valve leaflet in FK506-treated embryos, suggesting that, like *gata1* mutants, some abluminal cells may have failed to undergo MEndoT (S17G Fig). Cell counting in 98 hpf, FK506-treated embryos suggests that thick valves have an overabundance of VICs and a reduced number of cells at the AVC wall (S17F Fig), suggesting that some cells of the abluminal bilayer may be misassigned from luminal to abluminal cell fate and that cells of the AVC wall compensate by helping form the inner layer of the valve leaflet. We confirm this via photoconversion experiments (Fig 10). Together, this suggests that inhibition of Nfat signaling could cause defects in delamination, leading to thick valve phenotypes.

Finally, we questioned if inhibition of Nfat signaling could cause increased cell proliferation. To test this, we performed EdU staining in FK506-treated embryos and their DMSO treated controls. Our results suggest that, like *gata1* mutants, the superior AV valves of FK506-treated embryos have increased cell proliferation and that this increase is most likely attributable to the increased proliferation of cells misassigned from luminal to abluminal cell fate (Fig 9D, 9H and 9I).

Altogether, these results suggest that lower maximal wall shear stresses in the *gata1* mutant lead to lower Nfatc activity, which, in turn, leads to defects in delamination, increased cell proliferation, and thick, hyperplastic valves.

### *Twist1b* is down-regulated in both FK506-treated embryos and *gata1* mutants

**Fig 11:**
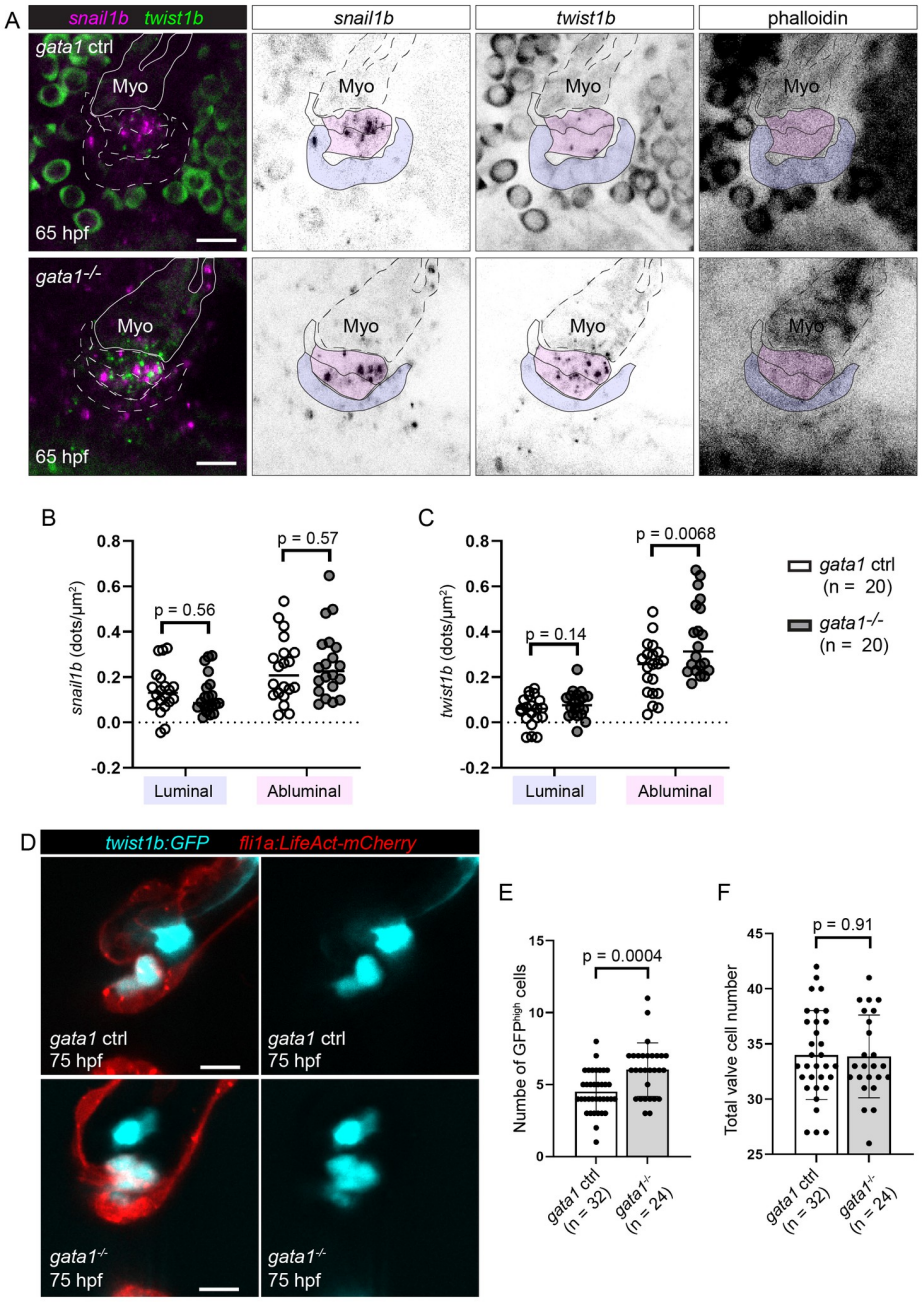
*Gata1* mutants have increased *twist1b*, but not *snail1b*, expression at 65 hpf. (A) Representative images of the developing superior AV valves at 65 hpf that have been stained using *snail1b* and *twist1b* probes in *gata1* controls (top row) and mutants (bottom row). Our interpretation of valve morphology superimposed on the images and blue (luminal) and magenta (abluminal) regions are used for quantifications in (B) and (C). (B, C) Dot plots showing the number of detected dots, each corresponding to1 *snail1b* (B) or 1 *twist1b* (C) mRNA molecule, in luminal and abluminal valve regions (shaded in purple and pink, respectively, in (A)). Statistical significance was calculated using Student *t* test. (D) Example images of *gata1* mutants and controls in the *Tg(twist*:*GFP);Tg(fli1a*:*Lifeact-mCherry)* background. (E) Graph showing the number of cells with bright GFP signal (GFP^high^ cells) in *Tg(twist*:*GFP);Tg(fli1a*:*Lifeact-mCherry) gata1* mutants and controls. Statistical significance was determined using Student *t* test. (F) Total valve cell number in the same embryos used for quantification in (E). Statistical significance was determined using Student *t* test. Scale bars: 10 μm. AV, atrioventricular valve; hpf, hours postfertilization; Myo, myocardium.

**Fig 12:**
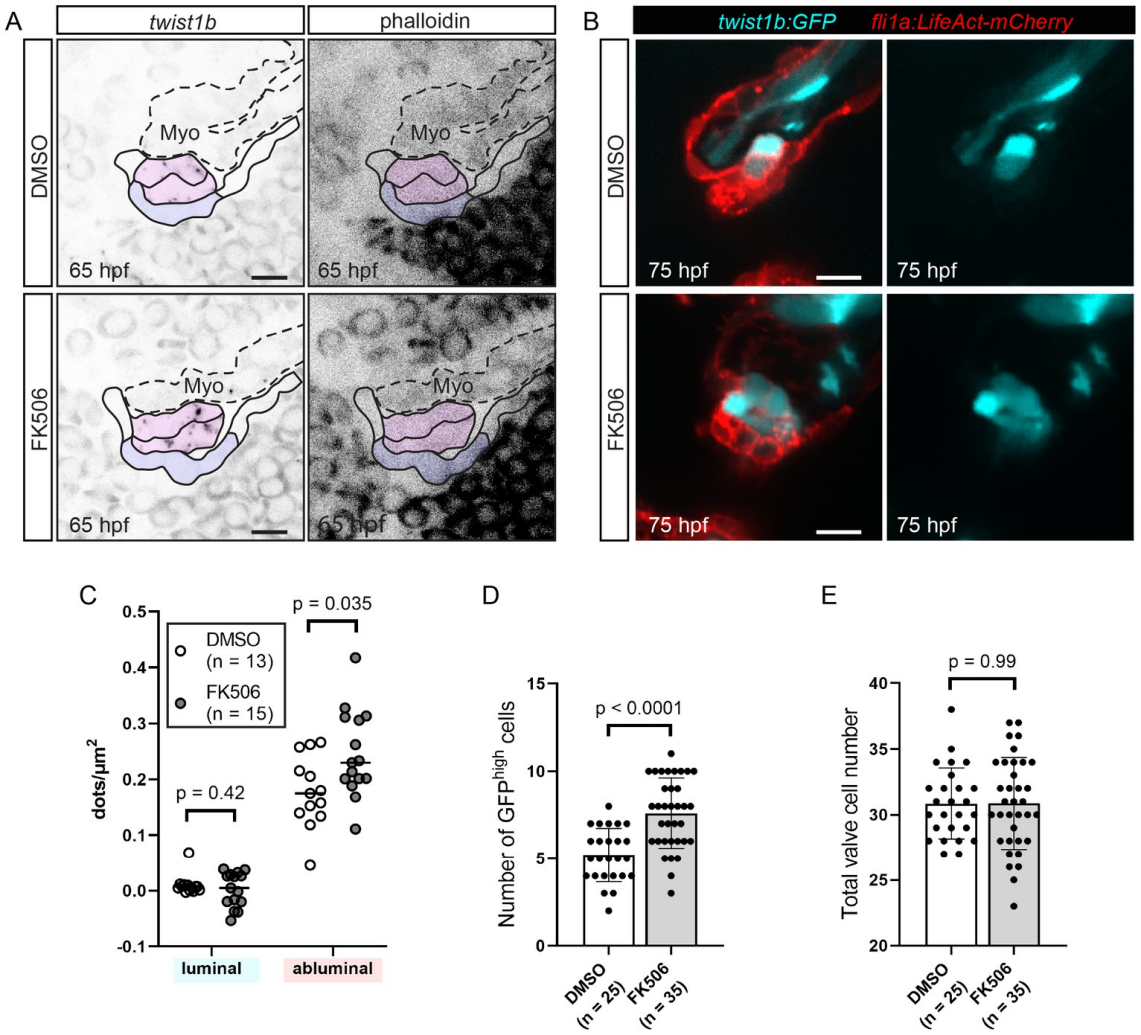
FK506-treated embryos have increased *twist1b* expression at 65 hpf. (A, C) Wild-type embryos were treated with DMSO or FK506 from 60 hpf, fixed at 65 hpf, and *twist1b* RNAscope was performed. (A) Representative images of developing superior AV valves at 65 hpf that have been stained using the *twist1b* probe in DMSO-treated controls (top row) and FK506-treated embryos (bottom row). Our interpretation of valve morphology superimposed on the images and blue (luminal) and magenta (abluminal) regions are used for quantifications in (C). (B, D, E) *Tg(twist*:*GFP);Tg(fli1a*:*Lifeact-mCherry)* embryos were treated with DMSO or FK506 from 60 hpf to 75 hpf. (B) Example images of DMSO-treated and FK506-treated embryos in the *Tg(twist*:*GFP);Tg(fli1a*:*Lifeact-mCherry)* background. (C) Dot plots showing the number of detected dots, each corresponding to 1 *twist1b* mRNA molecule in luminal and abluminal valve regions (shaded in purple and pink, respectively, in (A)). Statistical significance was calculated using Student *t* test. (D) Graph showing the number of cells with bright GFP signal (GFP^high^ cells) in *Tg(twist*:*GFP);Tg(fli1a*:*Lifeact-mCherry)* embryos treated with DMSO or FK506. Statistical significance was determined using Student *t* test. (E) Total valve cell number in the same embryos used for quantification in (D). Statistical significance was determined using Student *t* test. Scale bars: 10 μm. AV, atrioventricular valve; hpf, hours postfertilization; Myo, myocardium.

**Fig 13:**
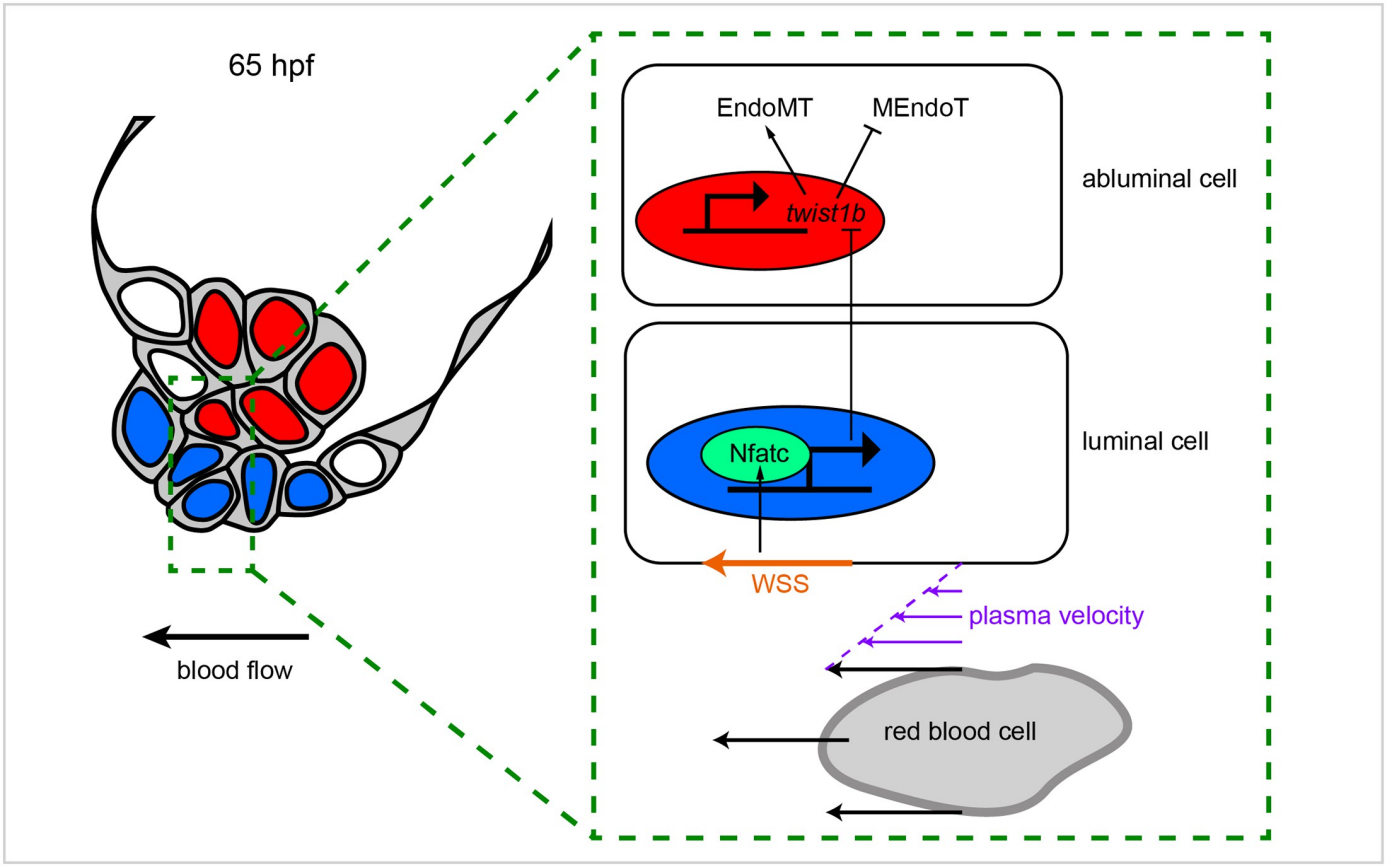
Proposed mechanotransduction pathway regulating valve delamination. At 65 hpf, just prior to delamination, WSS (orange arrow) activates Nfatc signaling in luminal cells. This leads to the production of a secreted signal that results in the inhibition of *twist1b* expression in abluminal cells. Since *twist1b* causes abluminal cells to transition toward more mesenchymal cell states, its inhibition is necessary to allow MEndoT, a process key to correct valve delamination. EndoMT, endothelial–mesenchymal transition; hpf, hours postfertilization; MEndoT, mesenchymal–endothelial transition; WSS, wall shear stress.

Since Nfat is activated in luminal cells, we wondered how a decrease in Nfat activity could lead to defects in MEndoT in abluminal cells. Since we have shown that *snail1b* and *twist1b* are expressed in abluminal cells in wild-type embryos (Fig 5), we wondered if their expression is changed in *gata1* mutants and FK506-treated embryos. To test this, we first assessed *snail1b* and *twist1b* expression in *gata1* mutants using RNAscope and found that *twist1b*, but not *snail1b*, is up-regulated in *gata1* mutants (Fig 11A–11C). Next, we sought to confirm that *twist1b* is up-regulated in *gata1* mutants by analyzing the *TgBAC(twist1b*:*GFP)* line in the *gata1* mutant background. We found that the number of cells expressing high levels of GFP (indicative of high cumulative expression of *twist1b* throughout the life of the embryo) is increased in mutants compared to controls (Fig 11D and 11E). Cell counting in the same embryos showed that total superior AV valve cell number is unchanged, indicating that the increase in the number of cells expressing high levels of GFP is due to changes in *twist1b* expression and not in increased proliferation of *twist1b* positive cells (Fig 11F). Finally, we performed *twist1b* RNAscope and analyzed *TgBAC(twist1b*:*GFP)* embryos treated with FK506. In doing so, we found that *twist1b* is up-regulated in FK506-treated embryos (Fig 12). We thus propose a model (Fig 13) whereby blood flow-derived wall shear stress is necessary to activate Nfatc in luminal cells prior to delamination and that the transcription of Nfat target genes leads to the inhibition of *twist1b* in abluminal cells, thereby preventing these abluminal cells from undergoing further EndoMT. Perturbation of this pathway leads to defects in delamination, resulting in thick, hyperplastic valves and decreased heart function.

## Discussion

Understanding the mechanobiology of developing valves is challenging due to the dynamic changes in force over 2 timescales: (1) Developing valves experience some of the largest and most rapid changes in mechanical force in the embryo within each heartbeat; and (2) cardiac forces due to heartbeat also change throughout development in concert with heart morphogenesis and changes in heart material composition [21,51–53]. Elucidating the overarching mechanisms by which cardiac forces govern valve morphogenesis thus requires careful examination of their role at different developmental stages, and currently, the role of cardiac forces during post-EndoMT stages of valve morphogenesis is poorly understood. Here, we show for the first time that zebrafish superior AV valve morphogenesis involves a delamination step after endocardial cells migrate into the CJ (Fig 1, S2 Fig). We additionally show that valve delamination in *gata1* mutants is delayed (S12 Fig) and that delamination, when it does occur, often occurs incorrectly, resulting in misassignment of cells from luminal to abluminal cell fate (Figs 6 and 7, S13 Fig). Misassigned cells proliferate, resulting in thick, hyperplastic valves with an overabundance of VICs (Fig 8). Finally, we show that decreased Nfatc activation in the endocardium is in part responsible for delamination defects in the *gata1* mutant (Figs 9 and 10, S17 Fig) and that the well-established EndoMT transcription factor *twist1b* is up-regulated in both *gata1* mutants and in embryos where Nfatc1 activity is inhibited (Figs 11 and 12), pointing toward a possible mechanotransduction pathway (Fig 13).

### Zebrafish AV valves form via tissue sheet delamination

The debate as to how zebrafish AV valves form has in part been fueled by experimental data and also by the fact that valves in different parts of the circulatory system form via different mechanisms. Since MEndoT has not been demonstrated in amniotic valves, the idea that all cells that migrate into the CJ undergo EndoMT and become future VICs is intuitive [26]. Perhaps due to this, various studies that have analyzed superior AV valve morphology between 72 and 80 hpf in fixed or stopped hearts have presumed that most valve cells at these stages are abluminal [21,22,25,54,55], contributing to a model whereby AV valves attain their leaflet morphology via the gradual process of elongation [20]. Such a model is attractive as it draws parallels to the formation of the septal leaflet of the mitral heart valve in amniotes [56]. Inconsistent with this idea, an earlier study has shown that thin superior AV valve leaflets can be seen when one images the beating heart at 72 hpf [24]. Most recently, a study analyzing stopped and fixed hearts has suggested that elongation is preceded by “VIC invasion,” whereby abluminal cells first undergo cellular rearrangement [23]. The authors note that only some abluminal cells become future VICs [23], leaving open the possibility that other mechanisms may be at play.

Here, we combine analyses of fixed/stopped hearts, beating hearts, and XYTZR time-lapse imaging to uncover how cellularized endocardial cushions transition into valve leaflets. We show that zebrafish superior AV valves first attain leaflet morphology via delamination, a rapid process that relies neither on the lengthening of the endocardial cushion nor cellular rearrangements (Fig 1, S2 and S3 Figs). Rather, delamination relies on the remodeling of cell–cell adhesions of cells that have already migrated to their correct location (Figs 1 and 3, S2 and S3 Figs). Delamination can occur as early as 72 hpf (thus explaining the observation of free-moving valve leaflets in the beating heart at this stage [24]) and is followed by valve elongation and the proliferation of abluminal cells at the valve base, the progeny of which migrate into the valve interstitial space to become VICs (Fig 2). This way of valve formation resembles the formation of mural heart valves and the septal leaflet of the tricuspid valve in amniotes [56]. However, among valve systems studied so far, the zebrafish superior AV valve appears unique in that delamination occurs via an apoptosis-independent process between 2 layers of endocardial-derived abluminal cells. Inherent to this process is the requirement that some cells transition toward a more mesenchymal state before transitioning back toward an endothelial cell state. Given that cells do not lose tight junctions throughout early migration and delamination stages (Fig 4), we suspect that EndoMT must be tightly controlled to ensure cells do not transition too far toward the mesenchymal state and retain their relative positions. While MEndoT has not been reported in any other valve system thus far, there is some evidence that MEndoT may be involved in cardiovascular processes such as angiogenesis [57] and artery reassembly [58,59]. Given that MEndoT is understudied relative to EndoMT, further investigation of zebrafish superior AV valve delamination may be a useful model to study how cells transition between EndoMT and MEndoT programs.

### Mechanotransduction during AV valve delamination

Previous studies in zebrafish have shown that cardiac forces regulate EndoMT during the early cell migration stage of valve formation [11,46,60–62], but whether they play a role in later stages of valve development is unclear. Here, we focus on valves growing under abnormal flow conditions that have managed to bypass early developmental defects. We find that endocardial Nfat activity is decreased in *gata1* mutants (Fig 9A and 9E). This leads to the up-regulation of EndoMT-promoting gene *twist1b* in abluminal cells (Fig 12), the incorrect assignment of cells from luminal to abluminal cell fate during valve delamination (Fig 10), and thick valves with an overabundance of VICs (Fig 9, S17 Fig). That endocardial Nfatc can suppress EndoMT seems to be conserved in mice [63,64]. Surprisingly, zebrafish mutants in which the sequence that encodes the DNA binding domain of *nfatc1* is deleted have been shown to have decreased *twist1b* expression in the AVC region and an abnormally low number of VICs at 4 dpf [23]. Possible reasons for the difference between the valve phenotypes of *nfatc1* mutants and FK506-treated embryos include (1) expression of other Nfatc proteins in the zebrafish valve endocardium whose activity is suppressed by FK506; (2) differences in the period in which Nfatc1 activity is down-regulated; (3) noncanonical actions of Nfatc1 in FK506-treated embryos; and (4) genetic compensation in the *nfatc1* mutant.

We find that although *gata1* mutant superior AV valves do not down-regulate the flow-sensitive transcription factor *klf2*, *klf2* mutants have thick superior AV valves and delamination delays (S16 Fig). Why *klf2* mutants that have bypassed early EndoMT defects could then develop thick valve phenotypes remains unclear, although recent work on how Klf2 functions mechanistically in developing valves allows us to postulate some hypotheses. Klf2a has been shown to act in parallel with Notch to inhibit Flt4, a protein that is thought to be expressed in abluminal cells of zebrafish superior AV valves and strongly expressed in cells undergoing EndoMT in mouse AV valves [55]. Meanwhile, mechanosensitive Notch-Delta-like-4 and Erk5-Klf2-Wnt9a signaling pathways act together to regulate early zebrafish superior AV valve formation [62]. Specifically, Notch-mediated lateral inhibition between endocardial cells is thought to single out Delta-like-4-positive endocardial cells, which acquire competence to respond to Wnt9a. Concurrently, Wnt9a is produced downstream of Klf2, leading to the ingression of Delta-like-4-positive endocardial cells [62]. Finally, Klf2-Wnt/β-catenin signaling is thought to limit mesenchymal cell proliferation in mouse valves during remodeling stages [17,22]. Taken together, one possible explanation for the thick valve phenotype in *klf2* mutants is that luminal cells at the delamination site require Klf2-Wnt signaling or Klf2-mediated Flt4 inhibition to break their VE-cadherin adhesions and release the valve leaflet from the AVC wall. Another possibility is that Klf2-Wnt/β-catenin signaling promotes MEndoT in abluminal cells during delamination stages. Most likely, Nfat and Klf2 mechanosensitive signaling pathways, each tuned to specific mechanical stimuli and mechanosensors [46], act synergistically to regulate both early valve EndoMT and valve delamination. However, how these pathways act together to accommodate the changing requirements in valve cell–cell adhesion remains to be determined.

Determining the precise aspects of cardiac force sensed by cells is an ongoing challenge in the field of mechanosensing. Here, we find that maximal wall shear stress is significantly decreased at the AVC at 65 hpf, while heartrate appears unaffected in *gata1* mutants (S10 and S11 Figs). Together with our finding that injection of a viscous medium into *gata1* mutants after cell migration can partially rescue the thick valve phenotype strongly suggests a role for wall shear stress in regulating valve delamination (S14 Fig). However, we cannot rule out other aspects of cardiac force (e.g., pressure and stretch) playing additional roles, nor do we know if cells are sensing oscillatory aspects of wall shear stress or simply its maximum amplitude. Given that the inferior AV valve leaflet begins to form later than the superior AV valve leaflet but appears to develop at roughly the same speed, there may be insights to be gained by performing more in-depth comparisons of force properties and Nfat activity levels in the superior versus the inferior regions of the AVC.

### A cellular model for studying the role of mechanical forces during valve delamination

In summary, we have identified a previously unappreciated step in zebrafish valve formation nestled between early cell migration stages [21,22] and valve maturation stages [23,65] that is critical to understanding how cellularized endocardial cushions morph into primitive, free-moving valve leaflets. We found that this step is sensitive to cardiac forces via a Nfatc-dependent mechanism and that disruption of this step can lead to thick valve phenotypes. Given that there are several congenital cardiac anomalies in humans associated with improper AV valve delamination, including Ebstein’s malformation, parachute mitral valve, and parachute-like asymmetric mitral valve [66–68], studying how cardiac forces affect delamination would be conducive toward finding better prevention methods and treatments for associated heart diseases.

## Materials and methods

### Zebrafish husbandry

Animal experiments were approved by the Animal Experimentation Committee of the Institutional Review Board of the Institut de Génétique et de Biologie Moléculaire et Cellulaire (IGBMC). All experiments were performed using ZF at 48 hpf, following the European directive 2010/63/EU and Home Office guidelines under the project license was PP6020928. Embryos were treated with 0.003% 1-phenyl-2-thiourea (PTU) (Sigma-Aldrich, St. Louis, USA, P7629) after 50% epiboly to prevent pigment formation. The zebrafish lines used in this study are Tg(fli1a:gal4FF^ubs^; UAS:kaede) [69], *Tg(fli1a*:*LifeAct-EGFP)* [70], *Tg(fli1a*:*gal4ff;UAS*:*EGFP-CAAX)* [71,72], *Tg(fli1a*:*myr-mCherry)* [73], *Tg*(*ve-cad*:*ve-cadTS*) [74], *Tg(gata1*:*dsRed)* [75], *Tg(fli1a*:*DsRed)* [76], *Tg(kdrl*:*EGFP)* [77], *Tg(fli1a*:*EGFP-Podxl)*^*ncv530Tg*^ (referred to as *Tg(fli1a*:*EGFP-Podxl)* in the text), *TgBAC(twist1b*:*GFP)*^*ncv534*^ [46], *Tg(4xnfbr*:*d2EGFP)*^*ncv531*^ [46], *gata1a* (*vlad tepes*^*m651*^) [78], *klf2a*^ig4^ [21], and *klf2b*^*ig5*^.

The *Tg(fli1a*:*EGFP-Podxl)*^*ncv530Tg*^ (referred to as *Tg(fli1a*:*EGFP-Podxl)* in the text), was generated using the Tol2 transposon system. The plasmid was generated by fusing EGFP after the signal peptide of zebrafish podocalyxin and then cloned into a Tol2 vector with the *fli1a* promoter.

The *klf2b*^*ig5*^ line was generated using a TALEN pair (left and right arms: 5′GGACATGGCTTTACCT-3′ and 5′AACGTTTGCAAACCAG-3′) were designed to target exon 1 of the *klf2b* gene and injected into single-cell wild-type (AB) first cell. We identified the alleles generated and confirmed that potential targeting events could be transmitted through the germline by outcrossing the F0 fish with AB animals and sequencing genomic DNA from pools of 6 F1 embryos. After screening the first generation, we focused on a 28-bp deletion mutation (5′-GGACATGGCTTTACCTTGCCTTTTGCCT-3′) leading to a premature stop codon in *klf2b* transcript. Studies were performed from F4 fish and later generations. A PCR-based genotyping strategy was established using the following primers to identify the wild-type and mutant alleles, the length of the deletion allowing their visualization directly on a 3%-agarose DNA gel: forward 5′- GGAAAGCGCGTATATTTGGA-3′, reverse 5′- CAAGTAGGAAATGCAAGTGT-3′ and sequencing forward primer 5′- AGAGCGCACTGTGCCTTATA-3′.

To obtain *gata1*^*−/−*^ embryos, *gata1*^*+/−*^ fish were incrossed and embryos without red blood cells were selected under a compound light microscope. The siblings of *gata1* homozygous mutants were used as controls. To obtain *klf2a*^*−/−*^ *klf2b*^*−/−*^ double mutants, *klf2a*^*−/−*^ *klf2b*^*−/−*^ fish were incrossed. For analyses of *klf2a*^*+/−*^ *klf2b*^*−/−*^ embryos, *klf2a* ^*+/+*^ *klf2b*^*+/−*^ fish were crossed with *klf2a*^*−/−*^ *klf2b*^*−/−*^ fish. After imaging the embryos, the images were analyzed, and the embryos were subsequently genotyped for *klf2b*.

For all experiments, embryos with improper heart looping at 48 hpf, curved tails, or left-right defects were excluded from analysis. Embryos with cells from the hatching gland obscuring the light path to the heart were also excluded from analysis. For analysis of wild-type embryos, embryos with pericardial edema were excluded from analysis. For analysis of mutants and FK506-treated embryos, embryos with pericardial edema between 65 and 72 hpf were excluded from analysis unless otherwise stated in the figure legend.

### Confocal and 2-photon imaging

Confocal and 2-photon imaging was performed using a Leica SP8 upright confocal microscope equipped with lasers operating at 488 nm, 561 nm, and 633 nm, a tunable multiphoton laser, and a Leica HCX IRAPO L, 25 ×, N.A. 0.95 objective. EGFP and Venus signals were imaged using the multiphoton laser tuned to 927-nm rather than the 488-nm laser to improve image quality in all experiments except for bead insertion experiments, where the signal from the bead is much higher than the signal from the EGFP or Venus when irradiated 927-nm light. In all experiments, detectors were set to photon counting mode.

### In vivo imaging of the beating heart

Fluorescent imaging of the beating heart was performed using a Leica DMi8 combined with a CSU-X1 (Yokogawa, Tokyo, Japan) spinning at 10,000 rpm, 2 simultaneous cameras (TuCam Flash4.0, Hamamatsu, Shizuoka, Japan), and a water immersion objective (Leica 40X, N.A. 1.1). Embryos were mounted in 0.7% low melting-point agarose (Sigma-Aldrich) in a glass-bottom petri dish (MatTek, Ashland, USA, P35G-0-14-C) and imaged at 28.5 °C at 100 frames per second. *Tg(fli1a*:*gal4ff;UAS*:*EGFP-CAAX)*, *Tg(fli1a*:*gal4ff;UAS*:*Kaede)*, and *Tg(fli1a*:*LifeAct-EGFP)* embryos were used to visualize if the AV valve has separated from the inner AVC wall. For experiments designed to visualize cell morphology at single-cell resolution, *Tg(fli1a*:*gal4ff;UAS*:*EGFP-CAAX)* embryos were incubated with 4 μM BODIPY TR Ceramide (Thermo Fisher Scientific, USA) for 20 minutes to stain the blood plasma and the CJ. To stop the heart without unmounting embryos, 2 mL of 100 mM BDM (Sigma-Aldrich) and 0.4% tricaine (Sigma-Aldrich) solution was added to the mounting dish. Once the hearts are seen to stop beating, 2 mL of normal embryo media is added to the dish, and the embryos are imaged within 10 minutes. Where necessary, realignment of the beating heart was performed postimaging using BeatSync2.0 [79].

Brightfield imaging of blood flow in 98 hpf *klf2* mutants was performed using a Leica DM IRBE microscope mounted with a Fastcam SA3 camera (Photron, Tokyo, Japan) and a water immersion objective (Leica 20X, N.A. 0.70). Embryos were mounted in 0.7% low melting-point agarose (Sigma-Aldrich) in a glass-bottom petri dish (Matek) and imaged at 500 frames per second.

### Bead injections

To occlude blood flow at the ventricle and thereby increase reversing flow at the AVC, beads (PureCube Glutathione MagBeads, Cube Biotech, Monheim am Rhein, Germany, 32225) of roughly 30 μm were inserted into the ventricle of the heart at 72 hpf using ultrafine tungsten manipulator probes (Ted Pella, Redding, USA, 13570) and tweezers. Briefly, embryos anesthetized with 0.02% tricaine (Sigma-Aldrich) were mounted in a mold [27] with 0.7% low melting point agarose (Sigma-Aldrich). Beads were then deposited on top of the low melting point agarose. Tungsten probes were used to create a hole in the center of the yolk and a path under the skin for the bead to follow from the yolk to the cardinal vein. A bead is pushed through the agarose and into the yolk using tweezers and then pushed anteriorly into the previously created path using a tungsten probe. The bead is then guided through the path using tweezers by gently pushing the bead anteriorly through the skin of the embryo. Once the bead is situated inside the cardinal vein, suction caused by the beating heart will cause the bead to move into the heart. Only hearts where the bead moves into the ventricle or the outflow tract were used for this study. Injected hearts where the bead remained in the atrium or moved beyond the outflow tract and exited the heart were not included in this study. Hearts with chambers that were collapsed 4 hours after bead injection were excluded from analysis. After insertion of the bead, embryos were allowed to develop normally in a 28.5 °C incubator. For sham embryos, the same surgical procedure is performed except that the cardinal vein was damaged using probes to mimic the damage caused by the bead, and the bead is not guided along the path to the cardinal vein, remaining in the yolk of the embryo instead.

### Drug treatments

To slow heart rate, embryos were transferred to 15 mM BDM. For both bead and drug experiments, embryos were stage matched and randomly assigned to control or treatment groups. For both bead and drug experiments, 3 hours 40 minutes after treatment, embryos were placed in 4 μM BODIPY-TR Ceramide (Invitrogen, ThermoFisher Scientific, Waltham, USA, D7540). Just before imaging, embryos were washed and anesthetized in 0.02% tricaine solution and then imaged using a spinning disk microscope to see if the AV valve has delaminated. Heartrate before imaging was measured at room temperature using a stereomicroscope and a timer. Heartrate at the time of imaging was measured based on movies obtained, while the embryos rested inside a 28.5 °C chamber.

To inhibit Nfatc1 activity, FK506 (Sigma-Aldrich) was used at a concentration of 2 μM.

### Immunohistochemistry

Embryos were fixed at the desired stage in 4% paraformaldehyde 3 to 4 hours at room temperature or overnight at 4 °C. After washing embryos in 1x PBST (PBS −0.1% Tween-20), the pericardial cavity of embryos was pierced using a 0.25-mm diameter stainless steel needle (Ted Pella), and the tails of the embryo were severed just posterior to the yolk extension. Embryos 48 to 50 hpf were permeabilized in 1x PBST containing 0.5% Triton-X 100 for 30 minutes at room temperature, embryos 55 to 60 hpf were permeabilized overnight at 4°C in 1X PBST containing 0.5% Triton-X 100 at 4 °C, and embryos older than 60 hpf were permeabilized overnight at 4 °C in 1x PBST containing 1% Triton-X 100. Embryos were then blocked overnight at 4 °C in 1x PBST supplemented with 5% BSA (anti-fibronectin) or 1% BSA and 10% NGS (anti-VECadherin, anti-ZO-1, and anti-Esama). Antibodies were used as follows: rabbit anti-fibronectin (F3648, Sigma-Aldrich) 1:100, rabbit anti-VECadherin [80] 1:500 (kind gift from Affolter lab), mouse anti-ZO-1 (Invitrogen) 1:100, rabbit anti-Esama 1:100 (kind gift from Affolter lab), goat anti-rabbit, and goat anti-mouse Alexa-647 secondary antibodies (Life Technologies, Carlsbad, USA, A-21235) 1:500. For embryos older than 80 hpf embryos, the skin of the embryo covering the heart was surgically removed using tungsten manipulator probes prior to imaging to reduce light scattering.

### RNAscope

Embryos were fixed overnight at 4 °C in 4% paraformaldehyde in PBS and were dehydrated using 100% methanol. RNAscope was performed using *snail1b-C3* and *twist1b-C1* probes from an RNAscope Fluorescent Multiplex kit (Advanced Cell Diagnostics, Newark, USA, 323110). TSA Plus fluorescein (PerkinElmer) and TSA cyanine 3 (PerkinElmer, Waltham, USA, NEL704A001KT) were used at 1:600 dilution. Embryos were kept for 3 days in 1:20 phalloidin 647 (Thermo Fisher Scientific) at 4 °C after the RNAscope protocol to stain F-actin. Embryos were imaged using a Leica SP8 confocal microscope equipped with an infrared, red, and 2-photon laser (set to 927 nm to image EGFP) and a Leica HCX IRAPO L, × 25, NA0.95 water immersion objective. For quantification, one z slice corresponding roughly to the middle of the valve was selected per embryo. Using Fiji, the integrated intensity and area of 20 isolated dots from the selected z slice and 2 neighboring z slices, and the different ROI were measured. The average number of dots per unit area was calculated by

$$Average\;dot\;number\;per\;unit\;area=\frac{{Dot\;number}_{ROI}}{{Area}_{ROI}},$$

where the total dot number in ROI is given by

$${Dot\;number}_{ROI}=\frac{{Intensity}_{ROI}-Background\times {Area}_{ROI}}{\overset{-}{{Intensity}_{dot}}}$$

The average intensity per single dot is given by

$$\overset{-}{{Intensity}_{dot}}=\frac{\sum_{i}^{20}Integrated\;intensity\;of\;doı(ı)-Background\times \sum_{i}^{20}area\;of\;of\;doı(ı)}{20},$$

and the background intensity is given by

$$Background=\frac{Integrated\;intensity\;of\;atrial\;ROI}{Area\;of\;atrial\;ROI\;}$$

Note that because there is a small measurement error associated with this method, when the number of dots in a valve region is very close to zero, the measured number of dots can be negative. The outline of the valve and valve cells is mainly determined using the background fluorescein stain, which labels cell membranes faintly. Where necessary, the determination of the outline of cells was also aided by the phalloidin channel, which stains red blood cells and the myocardium, as well as the brightfield channel, which can be useful for identifying the location of atrial endocardial cells.

### Photoconversion

Photoconversion was performed as previously described [27]. Briefly, embryos were mounted with the aid of a 3D printed mold in 0.7% low melting-point agarose supplemented with 50 mM BDM to inhibit heart contraction for the duration of the procedure. Photoconversion was performed using the FRAP module on an SP8 confocal microscope and a Leica HCX IRAPO L, × 25, NA0.95 water immersion objective. At 48 or 50 hpf, the region of interest exposed to 405 nm light to convert Kaede protein from its green form to its red form. A z-stack of the photoconverted heart was then acquired in the standard confocal mode to record the starting point of each experiment. Embryos were then removed from the agarose using a glass pipette, placed in fish water for 5 to 10 minutes until heart contraction resumed, and then put at 28.5 °C to develop individually under standard conditions until the time point of interest. Note that for analysis, we assume that photoconverted Kaede in the ventricle is degraded or diluted due to cell proliferation between 98 and 144 hpf.

### Electron microscopy

*Tg*(*ve-cad*:*ve-cadTS*) embryos were first imaged using a confocal microscope to ensure that VE-cadherin is down-regulated. The embryos were then fixed by immersion in 2.5% glutaraldehyde and 2.5% formaldehyde solution in 0.1M cacodylate buffer (PH = 7.2)) overnight at 4 °C. Embryos were rinsed 2 times in cacodylate buffer and followed by a 1 hour postfixation in 1% osmium tetroxide [OsO_4_] reduced by 1% potassium ferricyanide [K_3_Fe(CN)_6_] in dark on ice. Embryos were washed 1 time in cacodylate buffer and after extensive rinses in distilled water. The embryos were incubated in 1% uranyl acetate, for 2 hour on ice, and rinsed in water. Dehydration was performed in graded series of ethanol solutions (50%, 70%, 90%, and 100%; quickly rinsed and incubated for 20 minutes each), to be then infiltrated with epoxy 812. Semithin sections were cut at 2 μm by ultramicrotome (Leica Ultracut UCT) and stained with 1% Toluidine blue in 1% sodium borate, examined by Leica optical microscope (LEICA DMLB, Leica Microsystems, Germany). Ultrathin sections were cut at 70 nm and contrasted with uranyl acetate and lead citrate and examined at 70 kv with a Morgagni 268D electron microscope (FEI Electron Optics, Eindhoven, the Netherlands). Images were captured digitally by Mega View III camera (Soft Imaging System, Münster, Germany).

### Analysis of VE-cadherin (Cdh5) expression

In Fig 3B’–3B”, we used ImageJ to measure the average signal intensity of each cell–cell interface in the z-slice. For Fig 3B’, at 55 and 65 hpf, the average of the average signal intensity for cell–cell interfaces of luminal cells (depicted in purple Fig 3B) is divided by the average of the average signal intensity for cell–cell interfaces of abluminal cells (depicted in orange in Fig 3B). Similarly, at 80 hpf, the average of the average signal intensity of cell–cell interfaces of the outer layer of the valve (depicted in purple in Fig 3B) is divided by the average of the average signal intensity of cell–cell interfaces of the inner layer of the valve and the AVC (depicted in orange in Fig 3B). For Fig 3B”, the same method was used except that the average maximum signal intensity for cell–cell interfaces was used instead of the mean of the average signal intensity. Statistical significance relative to a theoretical value of 1 was calculated using the Wilcoxon rank-sum test.

### Analysis of podocalyxin localisation

For all podocalyxin quantifications, we first use Imaris to create a new coordinate system [22] and a new image stack (the x-axis points in the direction of the rise of the lumen, the y-axis points in the direction of the length of the lumen, and the z-axis in the direction of the span of the lumen). Z-slices are maximally 2 μm apart. For each z-slice, we use Fiji to measure the average green and red signal along the apical and basal sides of the cells. The data in the boxplots show the normalized ratio (green signal /red signal), where each data point corresponds to 1 embryo. For S7H, S7O, S7P and S7Q Fig, we use z-slices corresponding to the middle half of the valve for analysis. For S7K Fig, we use only z-slices in which cells with protrusions directed into the CJ are present.

### Phenotypic analyses

Cell counting and valve volume measurements were performed on Imaris software (Bitplane, Zürich, Switzerland). At 48 hpf, the AVC was defined using morphological landmarks where the edges of the AVC correspond to points of inflection of the endocardial wall. We classify valves as thick when they are 3 or more cell layers thick at the distal end of the valve or more than 3 cell layers thick at the proximal end of the valve. At 98 hpf, the inner layer of the valve may touch the AVC wall when the heart is stopped or fixed if the heart valve is very thick. Thus, for cell counting in these valves, we use the brightness of *fli1a* driven EGFP (tends to be down-regulated in VICs) and cell shape (organized for luminal cells) in addition to cell position to assign cells to “AVC wall,” “VIC,” or “Inner Layer” region categories. When imaging the beating heart, valves are counted as “delaminated” when a gap (sinus) is observed between the inner layer of the valve leaflet and the AVC wall at the central region of the valve.

### EdU incorporation assay

*Gata1* mutants and controls were transferred to 0.5 mmol/L EdU in egg water/0.5% dimethyl sulfoxide (DMSO) from 74 to 98 hpf. For FK506 experiments, EdU was added to media already containing DMSO or FK506 to reach a final concentration of 0.5 mmol/L EdU. The larvae were then fixed with 4% PFA for 3.5 hours at room temperature and washed with 1x PBST. The pericardial cavity was pierced using an ultrafine needle, the tails cut, and the entire larvae permeabilized overnight at 4°C in 1x PBST containing 1% Triton-X 100. Detection of EdU was performed using a Click-iT EdU Alexa Fluor 647 Kit (Thermo Fisher Scientific), where larvae were incubated in the Click-iT reaction cocktail for 1 hour. Embryos that show no staining in the heart and surrounding tissues were excluded from analysis.

### Shear stress modeling

In order to model shear stress at the AVC wall over the cardiac cycle, we treat blood in wild-type as a 2-phase solution, containing red blood cells and plasma, and blood in *gata1* mutants as a 1-phase solution, containing only plasma.

To estimate the viscosity of blood plasma, we use the empirical equation from Corcione’s work [81], which reviews various experimental works with nanoparticle volume fractions in the range from 0.0001 to 0.071, temperatures in the range between 293 K and 333 K, and particle sizes in the range from 25 to 200 nm. The empirical equation has a 1.84% standard deviation of error.

$$\frac{\mu_{eff}}{\mu_{f}}=\frac{1}{1-34.87{\left(\frac{d_{p}}{d_{f}}\right)}^{-0.3}\varphi^{1.03}}$$

$$d_{f}=0.1{\left(\frac{6M}{N\pi \rho_{f0}}\right)}^{1/3},$$

where μ_f_ and μ_eff_ are the dynamic viscosity of the base fluid and the effective dynamic viscosity of the nanofluid (N m^−2^s), M is the molecular weight of the base fluid, (kg mol^−1^), N is the Avogadro number (6.022e^23^ mol^−1^), *φ* is the volume fraction of the nanoparticles, ρ_f0_ is the mass density of the base fluid calculated at temperature T_0_ = 293 K (kg m^−3^), d_p_ is the diameter of the nanoparticle (m), and d_f_ is the equivalent diameter of a base fluid molecule (m). Zebrafish blood plasma has previously been reported to have a relatively constant dynamic viscosity of 1.5 mPa.s over a range of temperatures [82]. Zebrafish were kept at 28.5 °C (301.65 K), and the density of the plasma was approximated to be 997 kg m^−3^. We approximate the molecular weight of the plasma to be 100 kDa by averaging the protein profiles based on weight percentage [83] (using the table provided in the Supporting information of Li and colleagues’ work [83]).

To estimate the shear rate and shear stress experienced by cells of the AVC wall, we first image *Tg(flk*:*EGFP; gata1*:*DsRed)* embryos to measure red blood cell velocity and position in 65 hpf wild-type embryos. Images of the AVC wall and red blood cells were captured on the spinning disk at 1,000 frames per second for 2 cardiac cycles. Additional images of the entire heart were captured at 100 frames per second to ensure correct identification of the AVC wall contour. Pairwise image registration was used to track blood cells motion. Free-form image registration was performed with a grid of 5.6 microns, with an intensity mean square metric using Simple Elastix and an initialization with half the value of the previous time step’s deformation map to smooth the velocity changes. With image pixels above an intensity threshold identified as blood cells, shear rate is computed assuming a linear flow profile between the blood cell and the AVC surface for each cell that passes through near to the AVC wall. Shear stress values was then calculated (shear stress = shear rate × viscosity).

For the *gata1* mutants, XYTZ images of the heart were captured at a frame rate of 100 frames per second for 80 frames, and the beating heart was realigned using BeatSync2.0 [79]. We track the ventricle volumes across time with a motion estimation algorithm [40] and calculate flow rates via the rate of volume changes of the ventricle. Subsequently, AVC shear rate is estimated with the assumption of a parabolic flow profile across a maximum inscribed circle in the cross section of the AVC, thus providing conservatively maximum wall shear rate values in the *gata1* mutants. Maximal shear stress values were then calculated (shear stress = shear rate × viscosity).

### *Gata1* mutant rescue experiment

Anesthetized embryos were mounted in a glass bottom petri dish (Matek) with 0.7% low melting point agarose. 4.6 nl of water (controls) or nanoemulsion containing 3.3% of DiI dye salt with tetraphenylborate counterion, DiI-TPB [84], 95 nm red fluorescent lipid droplets in PBS (rescue) was injected into the bloodstream of 60 hpf *gata1* mutants via the common cardinal vein near the inflow of the heart. The dye-loaded nanoemulsion was made using a spontaneous emulsification procedure described in [84]. A Nanoject II (Drummond Scientific, Broomall, USA) microinjector, which requires backfilling with mineral oil, was used for injections. Because injected drops of nanoemulsion tend to stay stuck to the glass capilliary, the needle is kept within the common cardinal vein for about a minute after injection until the red nanoemulsion is seen to fully disperse in the circulation before pulling out the needle. After injection, embryos were carefully unmounted and returned to egg water with 0.003% PTU and the 28.5 °C incubator. Embryos were left to grow until 98 hpf when their hearts were stopped using BDM, and their superior AV valves were imaged using a confocal microscope (see “Confocal and 2-photon imaging” in the Materials and methods section).

To estimate the increase in blood viscosity caused by the injection of the nanoemulsion into the bloodstream of *gata1* mutants, a sample of the nanoemulsion was diluted 1:13 in PBS to match its expected concentration in the blood plasma and the viscosity of the diluted nanoemulsion was measured using a microfluidic rheometer (total blood volume was assumed to be 60 nl based on [85]). We found that the addition of the nanoemulsion to PBS increased its viscosity from 0.92 ± 0.02 cP to 1.10 ± 0.06 cP (*n* = 20, *p* < 0.005). The microfluidic rheometer comprised of a polydimethylsiloxane (PDMS, Dow Corning, USA) microchannel bonded to a microscope glass slide (VWR, USA) by oxygen plasma treatment. The channel had a square cross section with a 45-μm width. Flow was driven by a constant input pressure of 200 mbar with a pressure controller (MFCS-EZ, Fluigent, France). The samples were suspended with 2 um polystyrene tracer beads (0.1% v/v concentration, Thermo Fisher Scientific) for flow rate measurement using particle tracking. The microfluidic rheometer was calibrated using viscosity reference standards (Paragon Scientific, UK).

### Quantification of GFP^high^ and GFP^low^ cell number in the *Tg(twist1b*:*GFP line)*

Images of the whole heart and pericardial cavity were captured using the with laser intensity set so that the entire dynamic range of GFP signal is captured. GFP^high^ cells in the superior AV valve were classified as cells that can be seen to be GFP positive to the naked eye in autocontrasted images. GFP^low^ cells were classified as cells that can only be seen to be GFP positive when the brightness/contrast was further adjusted.

### Graphs, statistics, and image orientation

We did not compute or predict the number of samples necessary for statistical differences because the standard deviation of our study’s population was not known before starting our analysis. The number of embryos used for each experiment (*n*) is provided in the figures and/or figure legends. Notched Tukey boxplots were plotted using the IoSR Matlab Toolbox package **(**https://github.com/IoSR-Surrey/MatlabToolbox). The notch is centered on the median and extends to 1.58*IQR/sqrt(*N*), where *N* is the sample size. Bar graphs and dot plots in the manuscript were created using GraphPad Prism. The bar in dot plots marks the median. Error bars in bar graphs show standard deviation. No data points were excluded as outliers for analysis of statistical significance. Where exact *p*-values are not stated, stars represent ranges of *p*-values: n.s. *p* > 0.05; * *p* ≤ 0.05; ** *p* ≤ 0.01; *** *p* ≤ 0.001; **** *p* ≤ 0.0001. Images are orientated such that ventricle is on/toward the left, the atrium on/toward the right.

## Supporting information

S1 Fig Superior AV valves form via collective migration into the CJ followed by delamination. (A) Valve morphology as revealed using *Tg(fli1a*:*gal4ff;UAS*:*EGFP-CAAX)*. The stereotypical morphology of valves at different developmental stages is shown (each row shows the valve of a different embryo). Left column shows the whole heart. Middle column shows images of the valve corresponding to the blue-boxed region in the left column. Right column shows our interpretation of valve morphology. Scale bar left column: 50 μm; Scale bar middle column: 10 μm. (B, C) Frames from time-lapse movies using *Tg(fli1a*:*gal4ff;UAS*:*EGFP-CAAX)* embryos showing endocardial cells migrating collectively into the CJ. Images of the beating heart were acquired every hour from at 53 hpf (B, B’) or 49 hpf (C, C’) until 63 hpf. A single z slice of a single point in the cardiac cycle is shown. (B’) and (C’) show the yellow-boxed region of the heart shown in (B) and (C), respectively. The yellow arrowheads point to endocardial cells that extend their process into the CJ and subsequently leads the migration of other cells. Scale bar: 10 μm. At, atrium; AV, atrioventricular valve; CJ, cardiac jelly; hpf, hours postfertilization; V, ventricle.
(TIF)

S2 Fig Imaging valves in beating hearts reveals a delamination step during superior AV valve morphogenesis. (A–A””’) Images of a 76 hpf valve that has not delaminated. The same embryo was imaged twice, first while the heart was beating (A, A”, A””), then again once the heart has been stopped using BDM and tricaine (A’, A”’, A””’). (B–B””’) Similar to (A–A””’) except images show a 76 hpf valve that has delaminated. (A”””, A”””’) and (B”””, B”””’) shows our interpretation of the images in (A”, A”’) and (B”, B”’), respectively. The cyan arrowhead points to the gap seen between the inner layer of the leaflet and the AVC wall observed during heartbeat. (C) Frames from a time-lapse movie using a *Tg(fli1a*:*gal4ff;UAS*:*EGFP-CAAX)* embryo that has been immersed in BODIPY TR Ceramide undergoing valve delamination. The images shown correspond to the point in the cardiac cycle when valve cells are the least compressed. (C’) shows our interpretation of the images in (C) based on examination of the z-stack. A gap between the 2 cell layers is first observed at 81 hpf and the valve leaflet appears to be free moving by 82 hpf. Purple arrowheads point to a cell that remains inside the CJ to form one of the hinge cells of the valve leaflet. Cyan arrowheads point to the gap between the valve and the AVC wall. Scale bar in (A, A’, B, B’): 50 μm. Scale bar in (A”–A””’, B”–B””’, C): 10 μm. At, atrium; AV, atrioventricular valve; BDM, 2,3-butanedione monoxime; CJ, cardiac jelly; hpf, hours postfertilization; V, ventricle.
(TIF)

S3 Fig Abluminal hinge cells in newly delaminated leaflets are derived from endocardial cells of the ventricular edge of the AVC at 48 hpf. (A-A”’) Photoconversion experiments revealing the origin of cells in the newly formed valve leaflet. Each row shows an example of an embryo that has been photoconverted at 48 to 50 hpf, allowed to develop normally, and then imaged again at 80 hpf. Images at 80 hpf correspond to the point in the cardiac cycle when valve cells are the least compressed. Scale bar first column: 50 μm. Scale bar second column: 10 μm. (A) Example of an embryo where an AVC endocardial cell adjacent to the ventricle has been photoconverted. (A’) An example of an embryo where endocardial cells of the ventricle and the AVC have been photoconverted. (A”) An example of an embryo where AVC endocardial cells adjacent to the atrium have been photoconverted (A”’) An example of an embryo where endocardial cells of the ventricle have been photoconverted. (B) Example of an embryo where ventricular endocardial cells and AVC endocardial cells adjacent to the ventricle were photoconverted at 48 to 50 hpf, allowed to develop normally, and then imaged again at 98 hpf. Scale bar first column: 50 μm. Scale bar second column: 10 μm. (C) Model of valve leaflet formation. Cells are color-coded to show their fate. At 50 hpf, red cells represent ventricular endocardial cells. Yellow cells represent endocardial cells at the ventricular edge of the AVC. Green cells represent the remaining endocardial cells in the AVC. Blue cells represent atrial endocardial cells in the AVC. In subsequent stages, color schemes are kept to show the position and fate of cells over time. Cells derived from cells at 50 hpf due to cell proliferation are colored the same color as their mothers. Arrows in the top drawing indicate cell movements. Gray box marks period at which delamination can occur (65 to 80 hpf). Delamination itself takes place within 1 hour. At, atrium; AVC, atrioventricular canal; hpf, hours postfertilization; V, ventricle.
(TIF)

S4 Fig VE-cadherin expression is reexpressed during delamination stages. (A) Representative images of *Tg(fli1a*:*LifeAct-EGFP)* embryos immunostained with VE-cadherin (Cdh5) at time points between 48 and 120 hpf. The rightmost column shows the VE-cadherin signal overlaid on top of an image showing our interpretation of valve cell morphology based on the EGFP signal. Scale bar: 10 μm. Red arrowheads point to VE-cadherin positive cell–cell interfaces, cyan arrowheads point to cell–cell interfaces where VE-cadherin appears either very faint or absent. (B–B”) Quantification of immunostains shown in (A). (B) Schematic showing how “orange” and “purple” regions are specified and which cell–cell interfaces were used for measurement. (B’) Boxplots showing the mean VE-cadherin signal intensity of cell–cell interfaces in “orange” regions to mean VE-cadherin signal intensity of cell–cell interfaces in “purple” regions. *p*-Values above individual boxplots show levels of statistical significance between the mean ratio and a theoretical value of 1, as determined by a Wilcoxon rank-sum test. (B”) Same quantification as (B’), except the maximum VE-cadherin signal intensity is used instead of the mean VE-cadherin signal intensity. The data underlying both graphs can be found in S1 Data. hpf, hours postfertilization.
(TIF)

S5 Fig Tight junctions remain present when VE-cadherin expression is down. (A) Representative images of *Tg(fli1a*:*LifeAct-EGFP)* embryos co-immunostained with ZO-1 and Esama at 65 hpf. Red arrowheads point to cell–cell interfaces between abluminal cells where ZO-1 and Esama signals colocalize. Dotted lines outline the Myo. Scale bar: 10 μm. (B–B”’) *Tg(fli1a*:*DsRed);Tg(ve-cad*:*ve-cad-TS)* embryos at 65 hpf were fixed after confirming the down-regulation of Cdh5 via confocal imaging and sectioned for electron microscopy. (C) Electron microscopy image of the same 65 hpf embryo in Fig 3 showing the entire heart valve at low magnification. (B’) Medium magnification electron microscopy image corresponding to the red boxed region shown in (B). (B”–B”’) High magnification electron microscopy images corresponding to boxed regions in (B’). Nuclei in (B) have been pseudo-colored orange and green correspond to the same pseudo-colored nuclei in (B’), (B”), and (B”’). Blue arrowhead indicates an adherens junction. Yellow arrowheads indicate tight junctions. hpf, hours postfertilization; Myo, myocardium; ZO-1, zonula occludens-1.
(TIF)

S6 Fig FN expression at the basal side of the superior layer of abluminal cells is lost during delamination. FN immunostaining of developing valves in the *Tg(fli1a*:*LifeAct-EGFP)* background at different developmental stages between 48 and 98 hpf. Scale bar: 10 μm. Red arrowheads indicate the presence of FN surrounding abluminal cells at 60 and 65 hpf, blue arrowhead indicates the absence of FN around cells of the AVC wall at 80 hpf, and green arrowheads indicate the presence of FN between abluminal cells and luminal cells at 80 and 98 hpf. Rightmost column show schematics with our interpretation of results. FN, fibronectin; hpf, hours postfertilization.
(TIF)

S7 Fig Endocardial cells lose apical localization of podocalyxin during cell migration and regain it during delamination. Developing valves of *Tg(fli1a*:*myr-mCherry; fli1a*:*EGFP-Podxl)* embryos at developmental stages between 48 and 80 hpf. (A–C’) Representative example of a valve at 48 to 50 hpf where luminal cells have yet to send processes into the CJ. (A) shows the AVC as seen looking from the ventricle through the lumen to the atrium. Dotted white lines correspond to image planes shown in (B-B’) and (C-C’). (D-F’) Representative example of a valve at 48 to 50 hpf where some luminal cell(s) are sending processes into the CJ. (D) shows the AVC as seen looking from the ventricle through the lumen to the atrium. Dotted white lines correspond to image planes shown in (E–E’) and (F–F’). Orange arrowhead in (E–E’) points to a cell process directed into the CJ. (G) and (I, J) are schematics showing how valve regions were demarcated for quantification in (H) and (K), respectively. (H) and (K) are dot plots showing the ratio of GFP signal to mCherry signal at the apical membrane (solid line) over the ratio of GFP signal to mCherry signal at the basal membrane (dotted line) of the different valve regions. Values above 1 indicate preferential apical localization. Stars above dot plots indicate levels of statistical significance between the mean ratio and a theoretical value of 1, as determined by a Wilcoxon rank-sum test. (L–L’, M–M’, N–N’) Images of *Tg(fli1a*:*myr-mCherry; fli1a*:*EGFP-Podxl)* valves at 60, 65, and 80 hpf, respectively. Red arrows in L’ point to podocalyxin signal at the lateral membranes of abluminal cells. (L”, M”, N”) Schematics showing how valve regions are demarcated for analysis in (O, P, Q) respectively. (O, P, Q) are dot plots showing the ratio of GFP signal to mCherry signal at the apical membrane (solid line) over the ratio of GFP signal to mCherry signal at the basal membrane (dotted line) of the different valve regions. Values above 1 indicate preferential apical localization. Stars above dot plots indicate levels of statistical significance between the mean ratio and a theoretical value of 1, as determined by a Wilcoxon rank-sum test. The data underlying all the graphs can be found in S1 Data. AVC, atrioventricular canal; CJ, cardiac jelly; hpf, hours postfertilization.
(TIF)

S8 Fig Use of a transgenic reporter line for cumulative *twist1b* expression shows that *twist1b* is expressed in cells that underwent EndoMT. (A–A”) Three examples of 75 hpf hearts in the *TgBAC(twist1b*:*GFP);Tg(fli1a*:*LifeAct-mCherry)* background. The first column shows images of the entire heart. Scale bar: 50 μm. second to fourth columns show the superior AV valve and are zoomed in images corresponding to the boxed regions in the first column. Scale bars: 10 μm. Fifth column shows our interpretation of the valve in images of the second column. AV, atrioventricular valve; At, atrium; EndoMT, endothelial–mesenchymal transition; V, ventricle.
(TIF)

S9 Fig Perturbing mechanical forces associated with heartbeat inhibits splitting of the 2-cell layered structure. (A) Schematic of the method used showing bead insertion in the ventricle or yolk followed by immunostaining. (B) Flow profile in the AVC over 1 cardiac cycle for occluded and sham embryos, with the ends of the rectangle corresponding to the start of atrial systole. White, gray, and red regions show the fraction of the cardiac cycle corresponding to no flow, forward flow, and reversing flow, respectively. (C) Dot plots showing the heartrate of occluded and sham embryos at the time of imaging (76 hpf). Statistical difference between means was calculated via Student *t* test. (D) Representative images of the developing superior AV valve at 76 hpf, 4 hours after bead insertion, for occluded and sham embryos. The cardiac cycle was imaged at 100 frames per second and a single frame corresponding to when valve cells are not compressed is shown. Cyan arrowhead points to the gap between AVC wall and the inner layer of the valve leaflet. Scale bars: 50 μm. (E) Graph showing the percentage of split versus nonsplit valves for occluded and sham embryos. Dotted line shows the expected percentage of split valves at 72 hpf. Statistical significance was determined using Fisher exact test. (F) Representative images of the developing superior AV valve immunostained for VE-cadherin in occluded and sham embryos. Scale bars: 10 μm. (G) Quantification of VE-cadherin expression (Cdh5), analyzed by region as shown in the rightmost column in (F). *p*-Values were determined by Welch *t* test. (H) Graph showing heartrate measured at different time points for BDM-treated embryos and their controls. Time point t_0_ corresponds to just before treatment, t_10_ corresponds to 10 minutes after treatment, t_240_ corresponds to 240 minutes after treatment, and t_i_ corresponds to the time of imaging, when both treated embryos and controls have been placed in normal media with a low concentration of tricaine anesthetic. Statistical significance was calculated using Student *t* test. (I) Graph showing percentage of split versus nonsplit valves for BDM-treated embryos and their controls. Dotted line shows the expected percentage of split valves at 72 hpf. Statistical significance was determined using Fisher exact test. The data underlying all the graphs can be found in S1 Data. AV, atrioventricular valve; AVC, atrioventricular canal; BDM, 2,3-butanedione monoxime; hpf, hours postfertilization.
(TIF)

S10 Fig At 65 hpf, luminal cells of the AVC experience 3 to 4 times lower levels of maximal shear stress in *gata1* mutants compared to wild-type embryos. (A) Images of a 65 hpf wild-type embryo in the *Tg(kdrl*:*EGFP; gata1*:*DsRed)* background used for shear stress modeling. Red blood cells express DsRed and are tracked automatically. Segmented red blood cells are pseudo-colored according to their velocity. Dotted lines show the location of the AVC as observed using the EGFP signal. (B) Graph showing the modeled shear stress for wild-type and *gata1* mutants. Curves for embryos are aligned such that the AVC lumen is the widest at 125 ms. Centreline shows mean, shaded error shows the range of values. The 95th percentile for shear stress of wild type is calculated and smoothed with an envelope of ±2 time frames. (C) Examples of images taken of a *gata1* mutant in the *Tg(fli1a;gal4ff;UAS*:*Kaede)* background used for shear stress modeling. Values used to plot the graph can be found in S1 Data. At, atrium; AVC, atrioventricular canal; hpf, hours postfertilization; V, ventricle.
(TIF)

S11 Fig At 65 hpf, *gata1* mutants without pericardial edema have normal valve morphology. (A) Representative 3D views of the heart in a *gata1* control embryo and a *gata1* mutant embryo at 48 hpf. Cyan dots are markers of endocardial cell nuclei in the AVC as determined semiautomatically using Imaris software. (B) Dot plot showing the number of endocardial cells in the AVC at 48 hpf. (C) Representative images of 65 hpf *gata1* mutants with and without pericardial edema. Middle and right panels show the zoomed in images of boxed regions in the left panel. Yellow arrow points to the region where pericardial edema is most obvious. (D) Representative images of AV valves in 65 hpf *gata1* mutants with and without pericardial edema. Blue arrow points to an aberrant connection between abluminal cell structure and luminal AVC cells. (E) Graph showing number of abluminal cells in *gata1* controls and mutants at 65 hpf. (F) Graph showing heartrate of *gata1* controls and mutants at 65 hpf as calculated from movies of the beating heart taken at 100 frames per second. Statistical significance was calculated using Student *t* test. (J, K) *Gata1* controls (G) and mutants (H) immunostained for VE-cadherin (Cdh5) and ZO-1 at 65 hpf. All embryos had abluminal cells that down-regulated VE-cadherin but were immunopositive for ZO-1. Cyan arrows indicate cell–cell interfaces where VE-cadherin is down-regulated and ZO-1 signal is present. Scale bars: 20 μm. The data underlying all the graphs can be found in S1 Data. AV, atrioventricular valve; AVC, atrioventricular canal; hpf, hours postfertilization; ZO-1, zonula occludens-1.
(TIF)

S12 Fig *Gata1* mutants have delayed delamination. (A) *Gata1* controls and *gata1* mutants in the *Tg(fli1a*:*gal4ff;UAS*:*Kaede)* were stained with BODIPY TR Ceramide and imaged using the spinning disk at 100 frames per second at 80 hpf. Embryos with valves that have not delaminated at 80 hpf were unmounted and returned to the incubator to develop normally until 98 hpf when they were imaged again. Left column shows a *gata1* control valve that has delaminated at 80 hpf. Middle column shows a *gata1* mutant that has failed to delaminate at 80 hpf. Right column shows the same *gata1* mutant at 98 hpf, where the valve has delaminated. Top row shows the entire heart, bottom row shows the enlarged image of the boxed region. Cyan arrowheads indicate the gap between the valve and the AVC wall. Scale bars: 50 μm. (B) Graph showing percentage of valves that have split by 80 hpf, percentage of valves that have not split at 80 hpf but have split by 98 hpf, and valves that have not split by 98 hpf. The difference between the percentage of valves split at 80 hpf versus valves not split at 80 hpf was shown to be statistically significant using Fisher exact test. The data underlying the graph can be found in S1 Data. AVC, atrioventricular canal; hpf, hours postfertilization.
(TIF)

S13 Fig Photoconversion experiments show that AVC endocardial cells in 65 hpf *gata1* mutants do not undergo ectopic EndoMT. (A) Flow diagram summarizing the method used in this set of photoconversion experiments. At 48 to 50 hpf, embryonic hearts were stopped using BDM and atrial side of the AVC was photoconverted. The embryos were then returned to normal media and allowed to grow normally until 65 hpf, when the heart was stopped again using BDM and the valve imaged (first and second columns in (B–C’)). They were then returned to normal media and allowed to grow normally until 98 hpf, when the heart was stopped using BDM and they were imaged again (third and fourth columns in (B–C’)). (B–B’) Representative images of *gata1* controls showing that photoconverting the inferior layer of the abluminal bilayer or luminal endocardial cells of the AVC at 65 hpf does not result in photoconverted cells located abluminally at 98 hpf. (C–C’) Representative images of *gata1* mutants. Like *gata1* controls, photoconverting luminal endocardial cells of the AVC at 65 hpf does not result in photoconverted cells located abluminally at 98 hpf. AVC, atrioventricular canal; BDM, 2,3-butanedione monoxime; EndoMT, endothelial–mesenchymal transition; hpf, hours postfertilization.
(TIF)

S14 Fig Injection of a viscous solution into the bloodstream partially rescues the *gata1* mutant phenotype. (A) Schematic showing how we injected water or a solution containing lipid nanodroplets into the bloodstream. (B) Examples of 98 hpf *gata1* mutant valves injected with water (left panel) or lipid nanodroplets (middle and right panels). The number of VICs counted in each valve is shown. (C) Graph showing that the number of valves with normal thickness was not statistically different between fish that were injected with water and those that were injected with lipid nanodroplets. *p*-Values are based on Fisher exact test. (D) Graph showing that injecting *gata1* embryos with lipid nanodroplets results in fewer abluminal cells at 98 hpf. *p*-Values are based on the student *t* test. Scale bars: 10 μm. The data underlying both graphs can be found in S1 Data. hpf, hours postfertilization; VIC, valve interstitial cell.
(TIF)

S15 Fig Thick valves are associated with decreased valve function. (A) Images showing stereotypical *gata1* control and mutant fish with and without pericardial edema at 98 hpf, respectively. White asterisks highlight the swim bladder, which is underdeveloped in *gata1* mutants. Images on the right are zoomed in images corresponding to the boxed regions. Yellow arrow points to where pericardial edema is evident. (B) Dot plot showing heartrate of *gata1* mutants and controls at 80 hpf. (C) Dot plot showing heartrate of *klf2* mutants and controls at 80 hpf. (D, E) Images of *klf2* mutants and controls in the *Tg(fli1a*:*gal4ff;UAS*:*Kaede)* background. (D) Representative images of *klf2* mutant valves with and without pericardial edema at 65 hpf. Note that in the mutant with edema, there is only 1 layer of cells within the CJ. Embryos are screened between 65 and 80 hpf and those with pericardial edema are excluded from analyses shown in (E, F, and G). (E) Representative images of *klf2* control and mutant valves at 98 hpf. (F, G) Graph showing the percentage of valves at 98 hpf with thick or normal morphology in *klf2* controls and mutants (F), and in *klf2a*^*+/−*^ *klf2b*^*+/−*^ embryos and *klf2a*^*+/−*^ *klf2b*^*−/−*^ embryos (G). *p*-Values were calculated using Fisher exact test. (H) *Klf2* controls and mutants without pericardial edema at 80 hpf were used for a survival study. By 17 dpf, a significantly greater number of *klf2* mutant larvae have died based on the Gehan–Breslow–Wilcoxon statistical test. (I) Method used for assessing flow profiles of *klf2* mutants with normal or thick valves. (J) Example of a *klf2* control valve and 2 examples of *klf2* mutant superior AV valves at 98 hpf. Scale bars: 10 μm. (J’–J”’) Flow profiles across the AVC for the valves shown in left, middle, and right panels of (J), respectively. The ends of the rectangles corresponding to the start of atrial systole. White, gray, and red regions show the fraction of the cardiac cycle corresponding to no flow, forward flow, and RF, respectively. (K) The percentage of the cardiac cycle showing RF across the AVC was binned into 3 categories: 0%, greater than 0% but less than 20%, and greater than 20%. Graph showing the percentage of *klf2* control embryos, *klf2* mutant embryos where superior AV valves have normal thickness, and *klf2* mutant embryos where superior AV valves are abnormally thick with RF at 98 hpf. *p*-Values are based on Fisher exact test for embryos with greater than 20% of their cardiac cycle showing RF. The data underlying all the graphs can be found in S1 Data. AV, atrioventricular valve; CJ, cardiac jelly; hpf, hours postfertilization; RF, reversing flow.
(TIF)

S16 Fig *Klf2* mutants show defects in delamination, but *klf2* is up-regulated in *gata1* mutants. (A) Selected time frames of movies of the beating heart of 80 hpf *klf2a*^*+/−*^ *klf2b*^*+/−*^, *klf2a*^*+/−*^ *klf2b*^*−/−*^ and *klf2*^*−/−*^ mutants showing when the valve appears the least compressed. Cyan arrowhead indicates the gap between the inner layer of the valve leaflet and the AVC wall. Scale bar: 10 μm. (B) Representative images of *klf2*^*+/+*^ controls and *klf2*^*−/−*^ mutants where the ventricle and the ventricular edge of the AVC were photoconverted at 48 hpf (left column) and imaged again at 98 hpf (middle and right columns). In 8/8 *klf2*^*+/+*^ controls, all abluminal cells were photoconverted at 98 hpf. In 6/9 *klf2*^*−/−*^ mutants, some abluminal cells were not photoconverted (white asterisks). Scale bars: 10 μm. (C) Representative images of *gata1* mutant and control valves at 65 hpf that have been stained using *klf2a* and *klf2b* probes. (D,E) are dot plots showing the number of detected dots, each corresponding to 1 *klf2a* (D) or 1 *klf2b* (E) mRNA molecule, calculated for luminal endocardial cells of 2 different regions. Region 1 corresponds to the region shaded in pink in (C), while region 2 corresponds to the region shaded in light blue in (C). *p*-Values were determined using Student *t* test. Scale bars: 10 μm. (F) Graph showing percentage of *klf2a*^*+/−*^ *klf2b*^*+/−*^, *klf2a*^*+/−*^, *klf2b*^*−/−*^, and *klf2*^*−/−*^ mutant valves at 80 hpf that have delaminated, as determined from movies such as that shown in (A). Statistical significance was determined using Fisher exact test. The data underlying all the graphs can be found in S1 Data. At, atrium; AVC, atrioventricular canal; hpf, hours postfertilization; V, ventricle.
(TIF)

S17 Fig FK506-treated embryos have no delamination delay, but have pericardial edema, delamination defects, and thick valves. (A) Representative images of 80 hpf superior AV valves imaged in the beating heart of embryos treated with DMSO or FK506 from 60 to 80 hpf. Cyan arrowheads point to the gap between the AVC wall and the inner layer of the valve leaflet. Scale bars: 10 μm. (B) Representative images of 98 hpf superior AV valves imaged in embryos treated with DMSO or FK506 from 60 to 80 hpf. Scale bars: 10 μm. (C) Graph showing percentage of normal and thick valves in embryos treated with DMSO or FK506 from 60 to 80 hpf. *p*-Values were calculated using Fischer exact test. (D) Representative images of 104 hpf embryos that have been treated with DMSO or FK506 from 60 to 80 hpf. Right-side images are zoomed in images of the boxed regions. Yellow arrow points toward where pericardial edema is most obvious in the FK506-treated embryo. Yellow asterisks indicate the swim bladder, which is smaller or absent in FK506-treated embryos. (E) Graph showing percentage of embryos in (D) that have pericardial edema. *p*-Values were calculated using Fisher exact test. (F) Graph showing cell numbers by valve region at 98 hpf for embryos treated with DMSO or FK506 from 60 hpf. Statistical significance was determined by multiple *t* tests. (G) Representative images of embryos that have been treated with DMSO or FK506 from 60 hpf, fixed at 84 hpf, then immunostained for Cdh5 and ZO-1. White asterisks mark additional abluminal cells in FK506-treated embryos that express ZO-1 but not Cdh5. Scale bars: 10 μm. The data underlying all the graphs can be found in S1 Data. AVC, atrioventricular valve; hpf, hours postfertilization; ZO-1, zonula occludens-1.
(TIF)

S1 Movie Related to S1B and S1B’ Fig. Time-lapse imaging was performed using the *Tg(fli1a*:*gal4ff;UAS*:*EGFP-CAAX)* line starting at 53 hpf. The movie shows how a cell (segmented in blue) migrates into the CJ over 3 hours. The 3D heart was reconstructed for the entire cardiac cycle at each developmental time point, and the point of the cardiac cycle just before atrial contraction is shown. CJ, cardiac jelly; hpf, hours postfertilization.
(MOV)

S2 Movie Related to S1C and S1C’ Fig. Time-lapse imaging was performed using the *Tg(fli1a*:*gal4ff;UAS*:*EGFP-CAAX)* line starting at 49 hpf. The movie shows the heart at the 57 hpf time point of the time-lapse movie. The morphology of 3 cells (segmented yellow, magenta and blue) have processes directed into the CJ, forming 2 separate leading fronts for the collective migration of endocardial cells into the CJ. CJ, cardiac jelly; hpf, hours postfertilization.
(AVI)

S3 Movie Related to Fig 1E. Example of a newly formed valve leaflet at 72 hpf captured during imaging of the beating heart. The *Tg(fli1a*:*gal4ff;UAS*:*EGFP-CAAX)* embryo was treated with BODIPY TR. The movie first shows a single z slice as the movie cycles through the cardiac cycle. It then shows the z-stack of the valve at a time point when it is closed. Cyan arrows show the gap between the inner layer of the valve and the AVC wall. Note that there is still points of contact between the inner layer of the valve and the AVC at z position = 22 to 30 microns. AVC, atrioventricular canal; hpf, hours postfertilization.
(AVI)

S4 Movie Related to Fig 1I. Time-lapse imaging was performed for a photoconverted *Tg(fli1a*:*gal4ff;UAS*:*Kaede)* heart to capture the delamination process. The cardiac cycle of the AVC at consecutive developmental time points is shown (the movie is looped 3 times). Photoconverted cells are shown in magenta, nonphotoconverted cells are shown in green. The blue arrow points to where the tissue layers separate during each heartbeat.
(MP4)

S5 Movie Related to S2C Fig. Time-lapse imaging was performed for a *Tg(fli1a*:*gal4ff;UAS*:*EGFP-CAAX)* embryo treated with BODIPY TR ceramide to capture the valve delamination process. The movie here shows a single z-slice at consecutive developmental time points, starting at 80 hpf. hpf, hours postfertilization.
(MOV)

S6 Movie Related to S9D Fig. The cardiac cycle was imaged for *Tg(fli1a*:*gal4ff;UAS*:*EGFP-CAAX)* embryos that had a bead injected into the yolk (sham, left panel) or into the ventricle (occluded, right panel) at 72 hpf (membranes shown in green, bead is labeled with an asterisk). The embryo was treated with BODIPY TR ceramide (plasma, CJ and pericardial fluid shown in magenta) before imaging at 76 hpf. A single z-slice is shown. The blue arrow points to where the tissue layers of the delaminated valve separate during each heartbeat in the sham (left panel). Notice that the blood flows backward into the atrium after atrial contraction in embryo where blood flow is occluded (right panel). CJ, cardiac jelly; hpf, hours postfertilization.
(AVI)

S7 Movie Related to S12A Fig. An example of an 80 hpf *gata1* control embryo in the *Tg(fli1a*:*gal4ff;UAS*:*Kaede)* background that has been immersed with BODIPY TR ceramide where the superior AV has delaminated. AV, atrioventricular valve; hpf, hours postfertilization.
(AVI)

S8 Movie Related to Fig 10A. An example of an 80 hpf *gata1*^*−/−*^ embryo in the *Tg(fli1a*:*gal4ff;UAS*:*Kaede)* background that has been immersed with BODIPY TR ceramide where the superior AV has not delaminated. AV, atrioventricular valve; hpf, hours postfertilization.
(AVI)

S9 Movie Related to Fig 10A. The same embryo as that shown in Movie 8 at 98 hpf. At this stage, the superior AV has delaminated. AV, atrioventricular valve; hpf, hours postfertilization.
(AVI)

S10 Movie Related to Fig 6A. Example of a *gata1* control embryo in the *Tg(fli1a*:*gal4ff;UAS*:*Kaede)* background that has been stained for FN. The movie goes through the z stack to show 3D valve structure. FN, fibronectin.
(AVI)

S11 Movie Related to Fig 6A’. Example of a *gata1*^*−/−*^ embryo in the *Tg(fli1a*:*gal4ff;UAS*:*Kaede)* background that has been stained for FN. The movie goes through the z stack to show 3D valve structure. FN, fibronectin.
(AVI)

S12 Movie Related to S15J’ Fig. Example of a *klf2*^*+/+*^ embryonic heart showing no RF. In the top right corner, “0” indicates no flow from the ventricle to the AVC/atrium and “+” indicates positive flow from the AVC/atrium to the ventricle. AVC, atrioventricular canal; RF, reversing flow.
(AVI)

S13 Movie Related to S15J” Fig. Example of a *klf2*^*−/−*^ embryonic heart showing RF. In the top right corner, “0” indicates no flow from the ventricle to the AVC/atrium, “+” indicates positive flow from the AVC/atrium to the ventricle, and “-” indicates reversing flow from the ventricle to the AVC/atrium. AVC, atrioventricular canal; RF, reversing flow.
(AVI)

S1 Data Underlying data. (XLSX)

## Abbreviations

AV: atrioventricular valve
AVC: atrioventricular canal
BDM: 2,3-butanedione monoxime
CJ: cardiac jelly
EndoMT: endothelial–mesenchymal transition
hpf: hours postfertilization
IGBMC: Institut de Génétique et de Biologie Moléculaire et Cellulaire
MEndoT: mesenchymal–endothelial transition
Nfat: nuclear factor of activated T cells
PDMS: polydimethylsiloxane
PTU: 1-phenyl-2-thiourea
VIC: valve interstitial cell
ZO-1: zonula occludens-1
