# Supplementary figures and images for: Cardiac forces regulate zebrafish heart valve delamination by modulating Nfat signaling
Source: PLoS Biol. 2022 Jan 14;20(1):e3001505. doi: 10.1371/journal.pbio.3001505 (PMC8794269; doi:10.1371/journal.pbio.3001505)

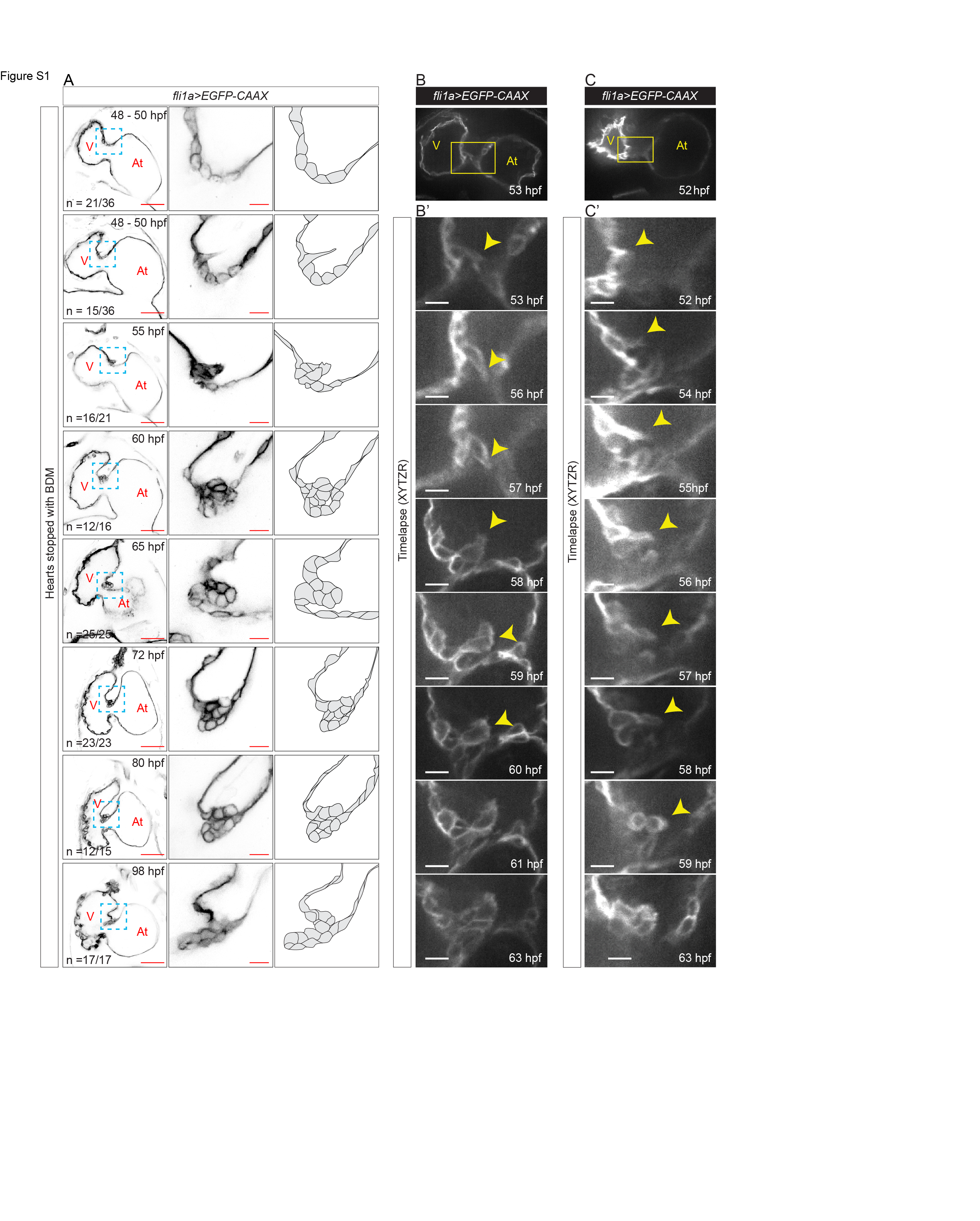

Supplement: S1 Fig — (A) Valve morphology as revealed using Tg(fli1a:gal4ff;UAS:EGFP-CAAX). The stereotypical morphology of valves at different developmental stages is shown (each row shows the valve of a different embryo). Left column shows the whole heart. Middle column shows images of the valve corresponding to the blue-boxed region in the left column. Right column shows our interpretation of valve morphology. Scale bar left column: 50 μm; Scale bar middle column: 10 μm. (B, C) Frames from time-lapse movies using Tg(fli1a:gal4ff;UAS:EGFP-CAAX) embryos showing endocardial cells migrating collectively into the CJ. Images of the beating heart were acquired every hour from at 53 hpf (B, B’) or 49 hpf (C, C’) until 63 hpf. A single z slice of a single point in the cardiac cycle is shown. (B’) and (C’) show the yellow-boxed region of the heart shown in (B) and (C), respectively. The yellow arrowheads point to endocardial cells that extend their process into the CJ and subsequently leads the migration of other cells. Scale bar: 10 μm. At, atrium; AV, atrioventricular valve; CJ, cardiac jelly; hpf, hours postfertilization; V, ventricle. (TIF) [file pbio.3001505.s001.tif]

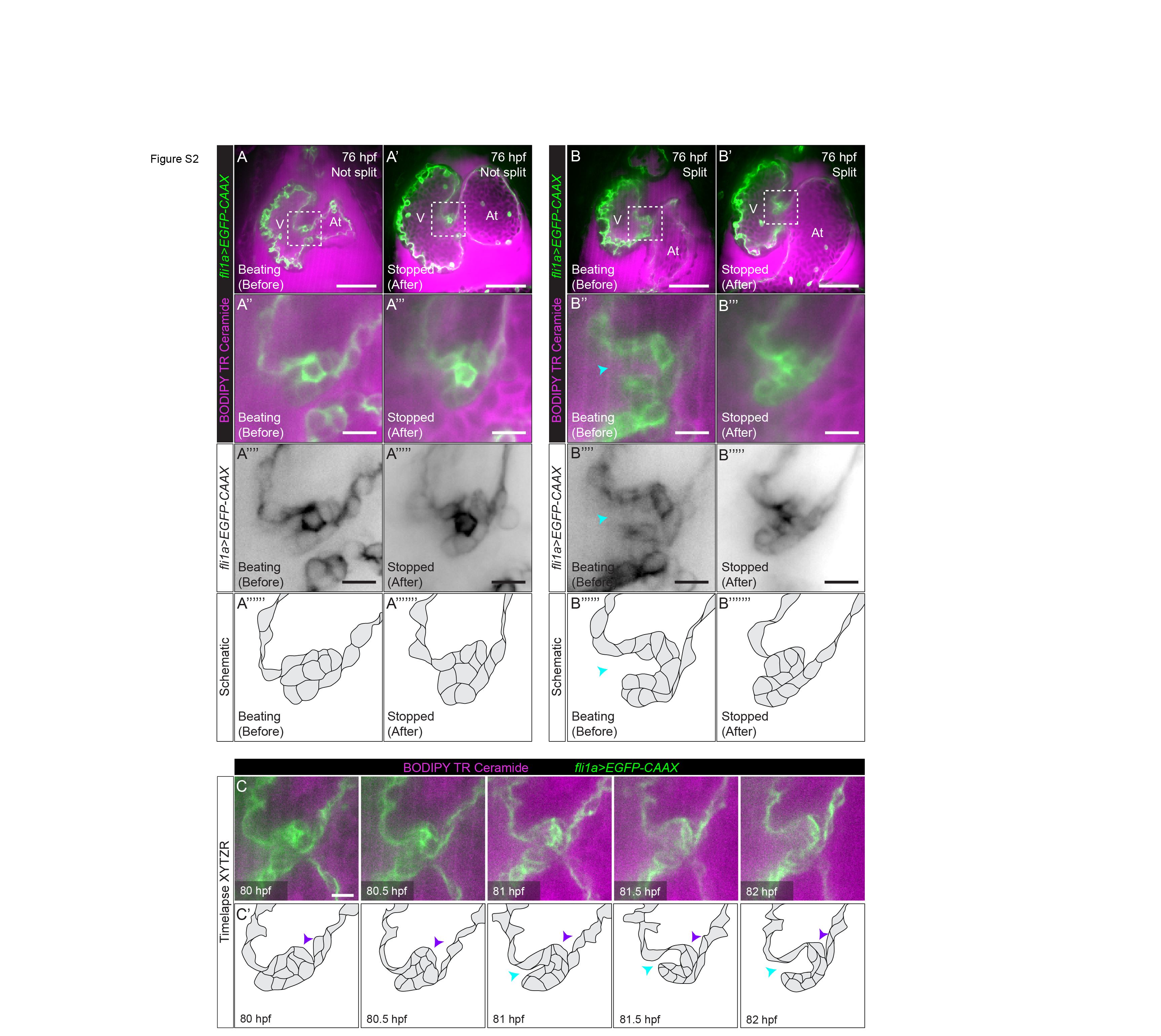

Supplement: S2 Fig — (A–A””’) Images of a 76 hpf valve that has not delaminated. The same embryo was imaged twice, first while the heart was beating (A, A”, A””), then again once the heart has been stopped using BDM and tricaine (A’, A”’, A””’). (B–B””’) Similar to (A–A””’) except images show a 76 hpf valve that has delaminated. (A”””, A”””’) and (B”””, B”””’) shows our interpretation of the images in (A”, A”’) and (B”, B”’), respectively. The cyan arrowhead points to the gap seen between the inner layer of the leaflet and the AVC wall observed during heartbeat. (C) Frames from a time-lapse movie using a Tg(fli1a:gal4ff;UAS:EGFP-CAAX) embryo that has been immersed in BODIPY TR Ceramide undergoing valve delamination. The images shown correspond to the point in the cardiac cycle when valve cells are the least compressed. (C’) shows our interpretation of the images in (C) based on examination of the z-stack. A gap between the 2 cell layers is first observed at 81 hpf and the valve leaflet appears to be free moving by 82 hpf. Purple arrowheads point to a cell that remains inside the CJ to form one of the hinge cells of the valve leaflet. Cyan arrowheads point to the gap between the valve and the AVC wall. Scale bar in (A, A’, B, B’): 50 μm. Scale bar in (A”–A””’, B”–B””’, C): 10 μm. At, atrium; AV, atrioventricular valve; BDM, 2,3-butanedione monoxime; CJ, cardiac jelly; hpf, hours postfertilization; V, ventricle. (TIF) [file pbio.3001505.s002.tif]

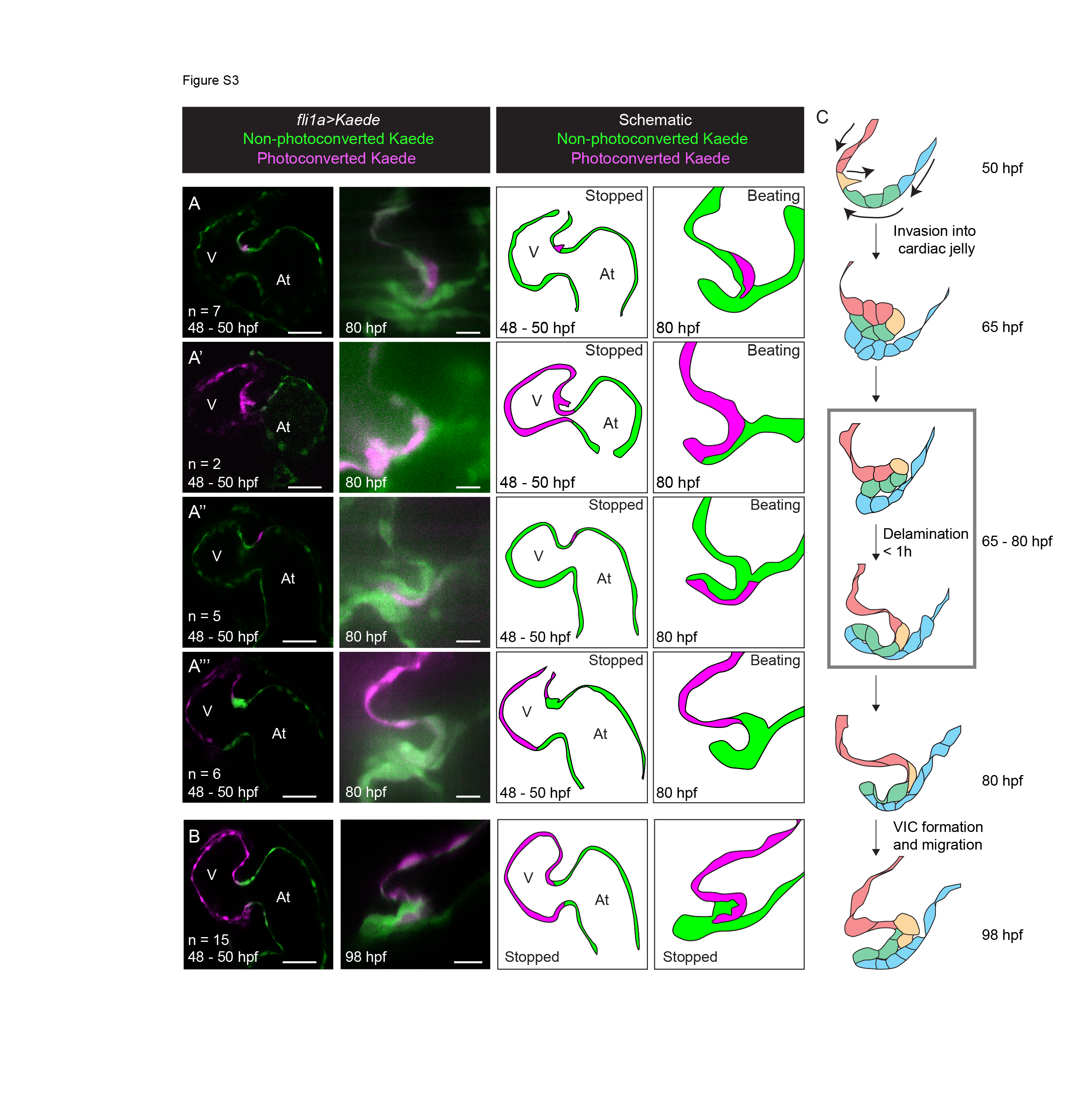

Supplement: S3 Fig — (A-A”’) Photoconversion experiments revealing the origin of cells in the newly formed valve leaflet. Each row shows an example of an embryo that has been photoconverted at 48 to 50 hpf, allowed to develop normally, and then imaged again at 80 hpf. Images at 80 hpf correspond to the point in the cardiac cycle when valve cells are the least compressed. Scale bar first column: 50 μm. Scale bar second column: 10 μm. (A) Example of an embryo where an AVC endocardial cell adjacent to the ventricle has been photoconverted. (A’) An example of an embryo where endocardial cells of the ventricle and the AVC have been photoconverted. (A”) An example of an embryo where AVC endocardial cells adjacent to the atrium have been photoconverted (A”’) An example of an embryo where endocardial cells of the ventricle have been photoconverted. (B) Example of an embryo where ventricular endocardial cells and AVC endocardial cells adjacent to the ventricle were photoconverted at 48 to 50 hpf, allowed to develop normally, and then imaged again at 98 hpf. Scale bar first column: 50 μm. Scale bar second column: 10 μm. (C) Model of valve leaflet formation. Cells are color-coded to show their fate. At 50 hpf, red cells represent ventricular endocardial cells. Yellow cells represent endocardial cells at the ventricular edge of the AVC. Green cells represent the remaining endocardial cells in the AVC. Blue cells represent atrial endocardial cells in the AVC. In subsequent stages, color schemes are kept to show the position and fate of cells over time. Cells derived from cells at 50 hpf due to cell proliferation are colored the same color as their mothers. Arrows in the top drawing indicate cell movements. Gray box marks period at which delamination can occur (65 to 80 hpf). Delamination itself takes place within 1 hour. At, atrium; AVC, atrioventricular canal; hpf, hours postfertilization; V, ventricle. (TIF) [file pbio.3001505.s003.tif]

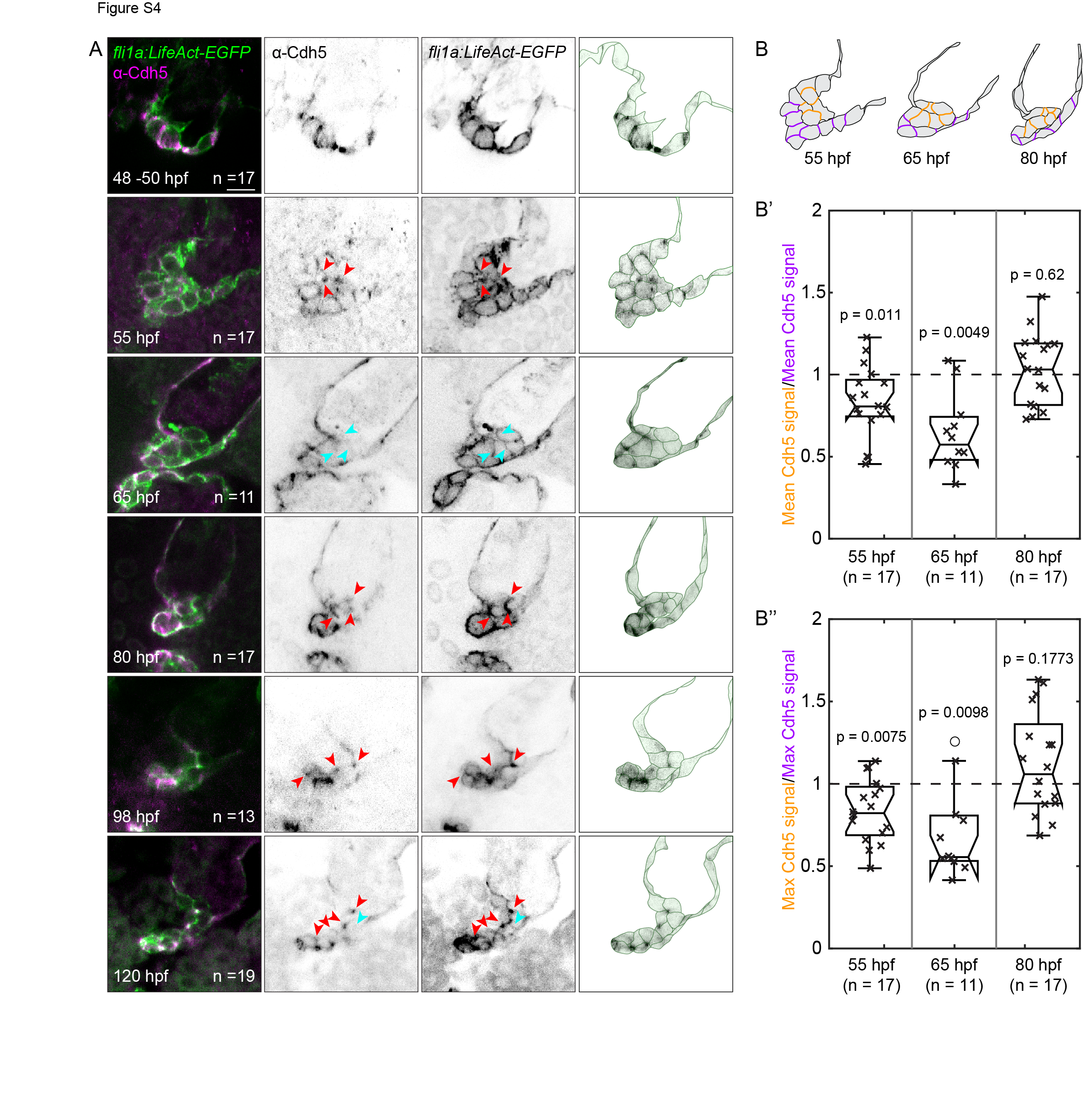

Supplement: S4 Fig — (A) Representative images of Tg(fli1a:LifeAct-EGFP) embryos immunostained with VE-cadherin (Cdh5) at time points between 48 and 120 hpf. The rightmost column shows the VE-cadherin signal overlaid on top of an image showing our interpretation of valve cell morphology based on the EGFP signal. Scale bar: 10 μm. Red arrowheads point to VE-cadherin positive cell–cell interfaces, cyan arrowheads point to cell–cell interfaces where VE-cadherin appears either very faint or absent. (B–B”) Quantification of immunostains shown in (A). (B) Schematic showing how “orange” and “purple” regions are specified and which cell–cell interfaces were used for measurement. (B’) Boxplots showing the mean VE-cadherin signal intensity of cell–cell interfaces in “orange” regions to mean VE-cadherin signal intensity of cell–cell interfaces in “purple” regions. p-Values above individual boxplots show levels of statistical significance between the mean ratio and a theoretical value of 1, as determined by a Wilcoxon rank-sum test. (B”) Same quantification as (B’), except the maximum VE-cadherin signal intensity is used instead of the mean VE-cadherin signal intensity. The data underlying both graphs can be found in S1 Data. hpf, hours postfertilization. (TIF) [file pbio.3001505.s004.tif]

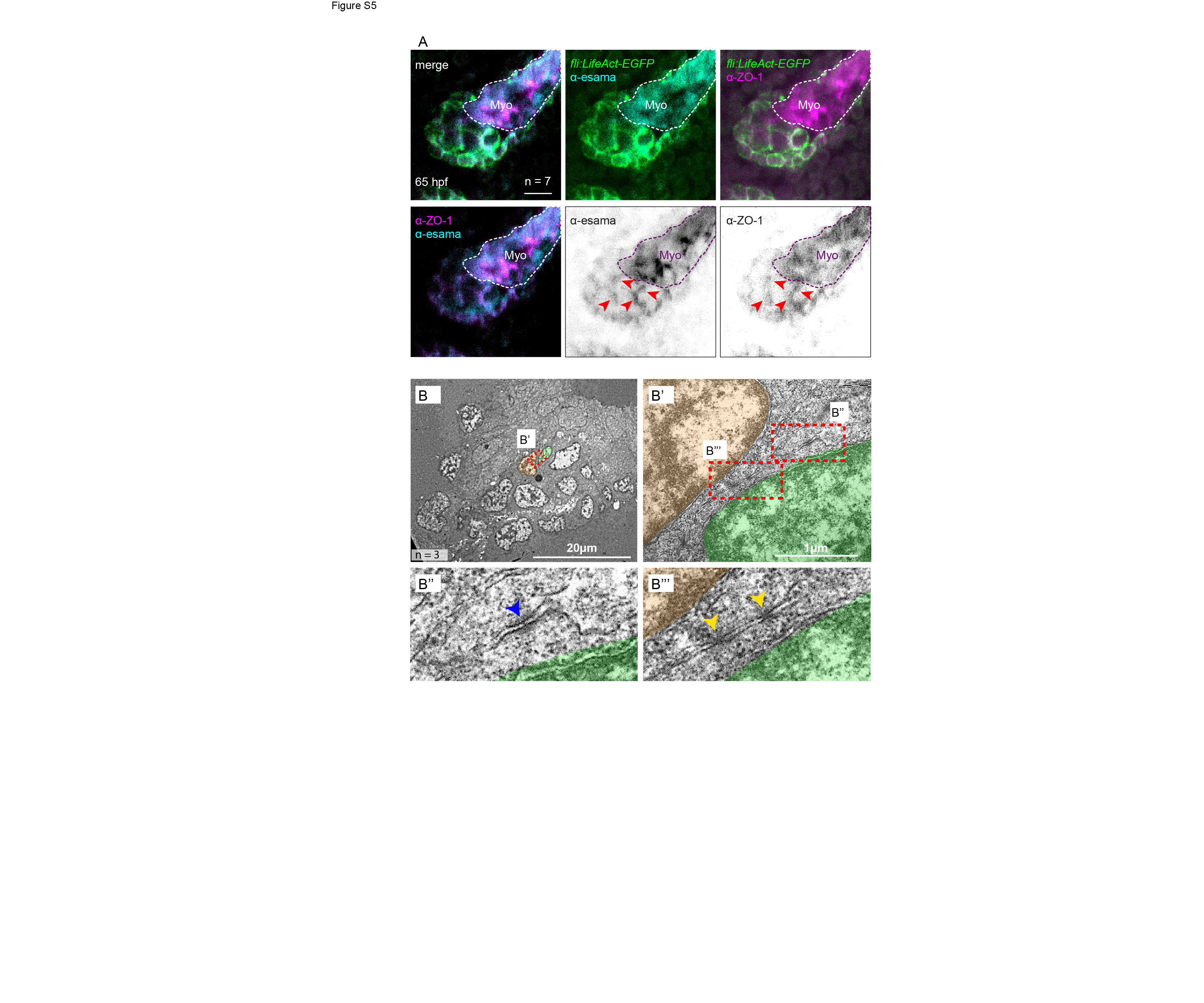

Supplement: S5 Fig — (A) Representative images of Tg(fli1a:LifeAct-EGFP) embryos co-immunostained with ZO-1 and Esama at 65 hpf. Red arrowheads point to cell–cell interfaces between abluminal cells where ZO-1 and Esama signals colocalize. Dotted lines outline the Myo. Scale bar: 10 μm. (B–B”’) Tg(fli1a:DsRed);Tg(ve-cad:ve-cad-TS) embryos at 65 hpf were fixed after confirming the down-regulation of Cdh5 via confocal imaging and sectioned for electron microscopy. (C) Electron microscopy image of the same 65 hpf embryo in Fig 3 showing the entire heart valve at low magnification. (B’) Medium magnification electron microscopy image corresponding to the red boxed region shown in (B). (B”–B”’) High magnification electron microscopy images corresponding to boxed regions in (B’). Nuclei in (B) have been pseudo-colored orange and green correspond to the same pseudo-colored nuclei in (B’), (B”), and (B”’). Blue arrowhead indicates an adherens junction. Yellow arrowheads indicate tight junctions. hpf, hours postfertilization; Myo, myocardium; ZO-1, zonula occludens-1. (TIF) [file pbio.3001505.s005.tif]

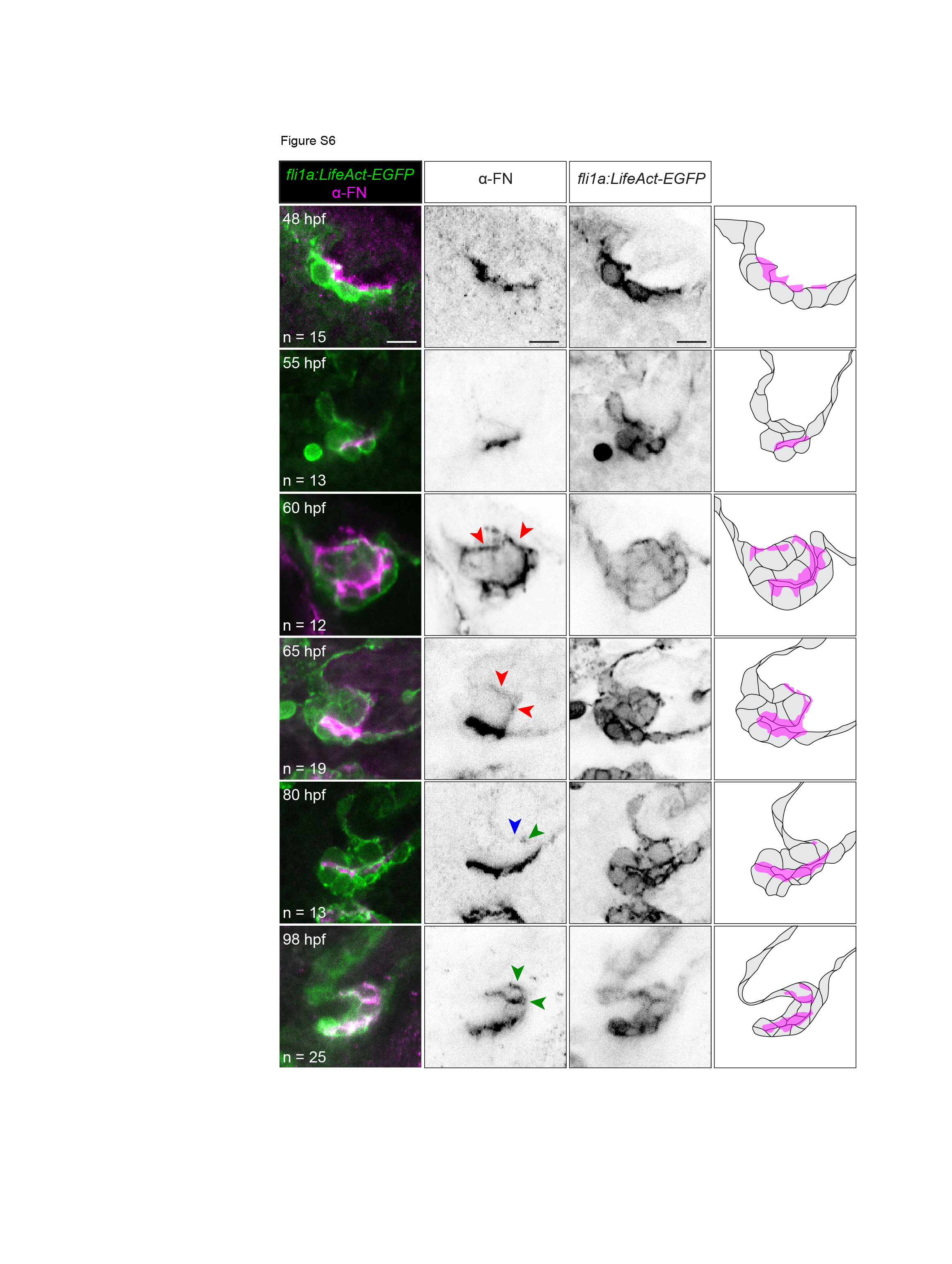

Supplement: S6 Fig — FN immunostaining of developing valves in the Tg(fli1a:LifeAct-EGFP) background at different developmental stages between 48 and 98 hpf. Scale bar: 10 μm. Red arrowheads indicate the presence of FN surrounding abluminal cells at 60 and 65 hpf, blue arrowhead indicates the absence of FN around cells of the AVC wall at 80 hpf, and green arrowheads indicate the presence of FN between abluminal cells and luminal cells at 80 and 98 hpf. Rightmost column show schematics with our interpretation of results. FN, fibronectin; hpf, hours postfertilization. (TIF) [file pbio.3001505.s006.tif]

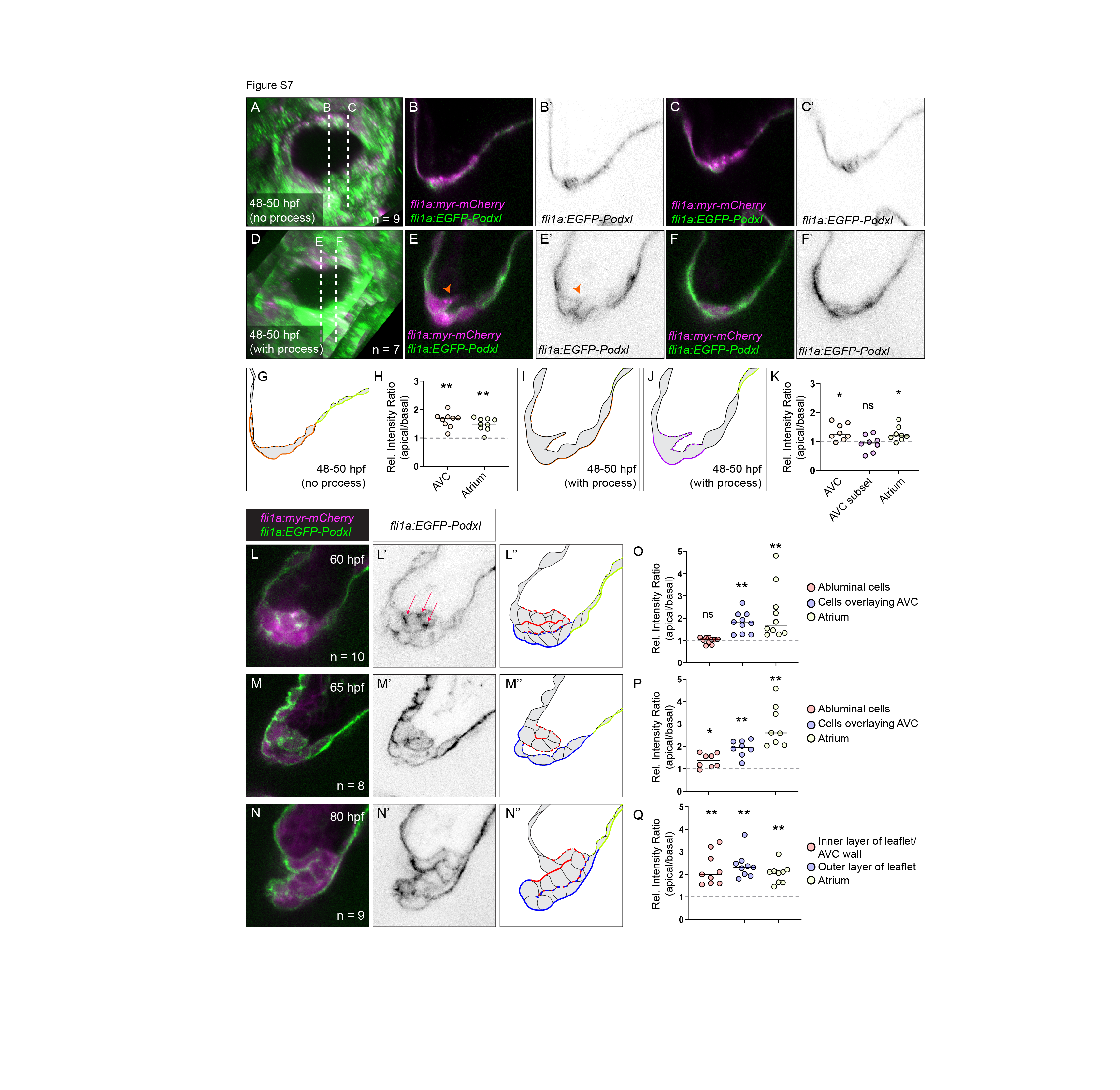

Supplement: S7 Fig — Developing valves of Tg(fli1a:myr-mCherry; fli1a:EGFP-Podxl) embryos at developmental stages between 48 and 80 hpf. (A–C’) Representative example of a valve at 48 to 50 hpf where luminal cells have yet to send processes into the CJ. (A) shows the AVC as seen looking from the ventricle through the lumen to the atrium. Dotted white lines correspond to image planes shown in (B-B’) and (C-C’). (D-F’) Representative example of a valve at 48 to 50 hpf where some luminal cell(s) are sending processes into the CJ. (D) shows the AVC as seen looking from the ventricle through the lumen to the atrium. Dotted white lines correspond to image planes shown in (E–E’) and (F–F’). Orange arrowhead in (E–E’) points to a cell process directed into the CJ. (G) and (I, J) are schematics showing how valve regions were demarcated for quantification in (H) and (K), respectively. (H) and (K) are dot plots showing the ratio of GFP signal to mCherry signal at the apical membrane (solid line) over the ratio of GFP signal to mCherry signal at the basal membrane (dotted line) of the different valve regions. Values above 1 indicate preferential apical localization. Stars above dot plots indicate levels of statistical significance between the mean ratio and a theoretical value of 1, as determined by a Wilcoxon rank-sum test. (L–L’, M–M’, N–N’) Images of Tg(fli1a:myr-mCherry; fli1a:EGFP-Podxl) valves at 60, 65, and 80 hpf, respectively. Red arrows in L’ point to podocalyxin signal at the lateral membranes of abluminal cells. (L”, M”, N”) Schematics showing how valve regions are demarcated for analysis in (O, P, Q) respectively. (O, P, Q) are dot plots showing the ratio of GFP signal to mCherry signal at the apical membrane (solid line) over the ratio of GFP signal to mCherry signal at the basal membrane (dotted line) of the different valve regions. Values above 1 indicate preferential apical localization. Stars above dot plots indicate levels of statistical significance between the mean ratio and a [file pbio.3001505.s007.tif]

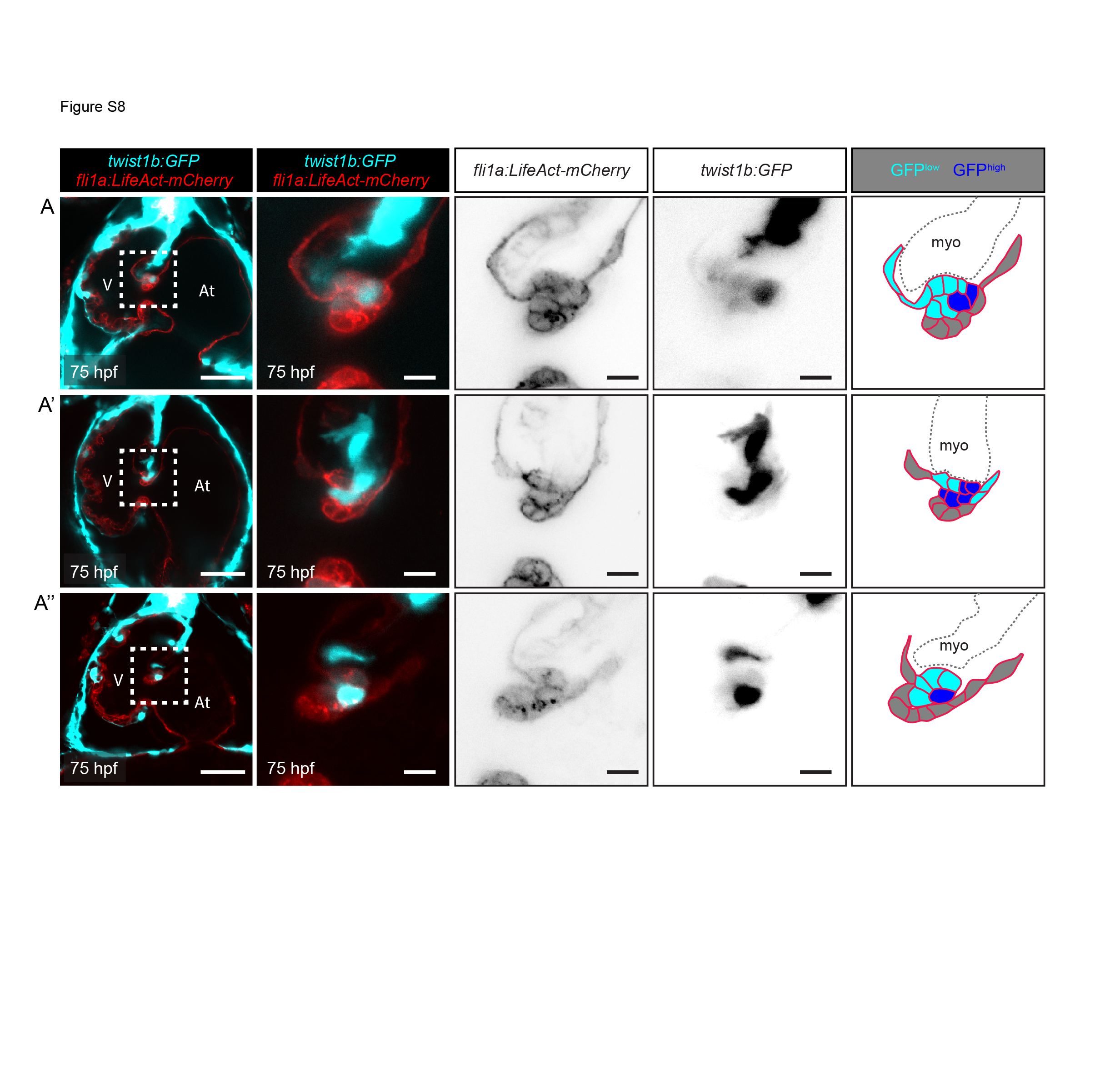

Supplement: S8 Fig — (A–A”) Three examples of 75 hpf hearts in the TgBAC(twist1b:GFP);Tg(fli1a:LifeAct-mCherry) background. The first column shows images of the entire heart. Scale bar: 50 μm. second to fourth columns show the superior AV valve and are zoomed in images corresponding to the boxed regions in the first column. Scale bars: 10 μm. Fifth column shows our interpretation of the valve in images of the second column. AV, atrioventricular valve; At, atrium; EndoMT, endothelial–mesenchymal transition; V, ventricle. (TIF) [file pbio.3001505.s008.tif]

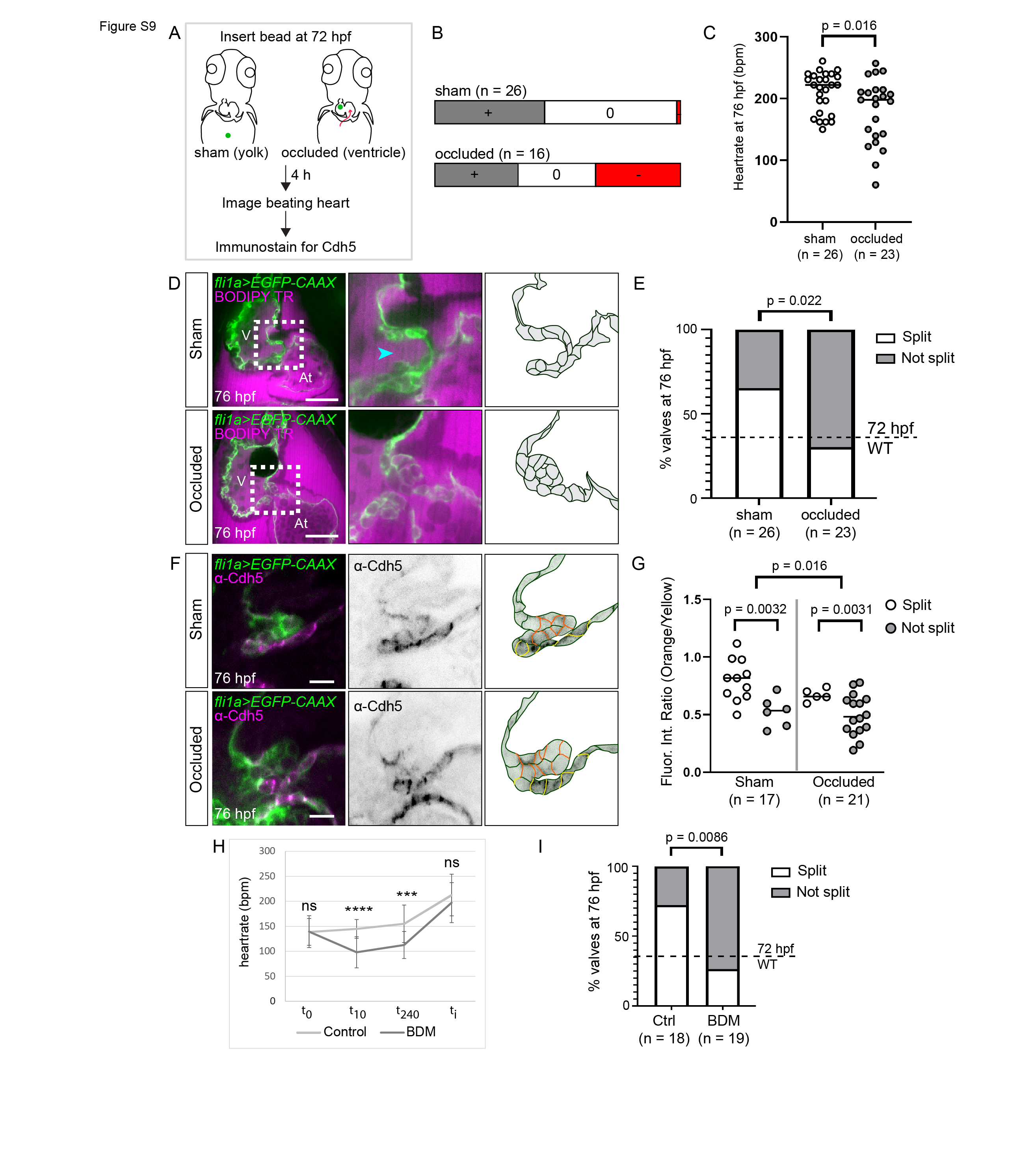

Supplement: S9 Fig — (A) Schematic of the method used showing bead insertion in the ventricle or yolk followed by immunostaining. (B) Flow profile in the AVC over 1 cardiac cycle for occluded and sham embryos, with the ends of the rectangle corresponding to the start of atrial systole. White, gray, and red regions show the fraction of the cardiac cycle corresponding to no flow, forward flow, and reversing flow, respectively. (C) Dot plots showing the heartrate of occluded and sham embryos at the time of imaging (76 hpf). Statistical difference between means was calculated via Student t test. (D) Representative images of the developing superior AV valve at 76 hpf, 4 hours after bead insertion, for occluded and sham embryos. The cardiac cycle was imaged at 100 frames per second and a single frame corresponding to when valve cells are not compressed is shown. Cyan arrowhead points to the gap between AVC wall and the inner layer of the valve leaflet. Scale bars: 50 μm. (E) Graph showing the percentage of split versus nonsplit valves for occluded and sham embryos. Dotted line shows the expected percentage of split valves at 72 hpf. Statistical significance was determined using Fisher exact test. (F) Representative images of the developing superior AV valve immunostained for VE-cadherin in occluded and sham embryos. Scale bars: 10 μm. (G) Quantification of VE-cadherin expression (Cdh5), analyzed by region as shown in the rightmost column in (F). p-Values were determined by Welch t test. (H) Graph showing heartrate measured at different time points for BDM-treated embryos and their controls. Time point t0 corresponds to just before treatment, t10 corresponds to 10 minutes after treatment, t240 corresponds to 240 minutes after treatment, and ti corresponds to the time of imaging, when both treated embryos and controls have been placed in normal media with a low concentration of tricaine anesthetic. Statistical significance was calculated using Student t test. (I) Graph showing percentage of spl [file pbio.3001505.s009.tif]

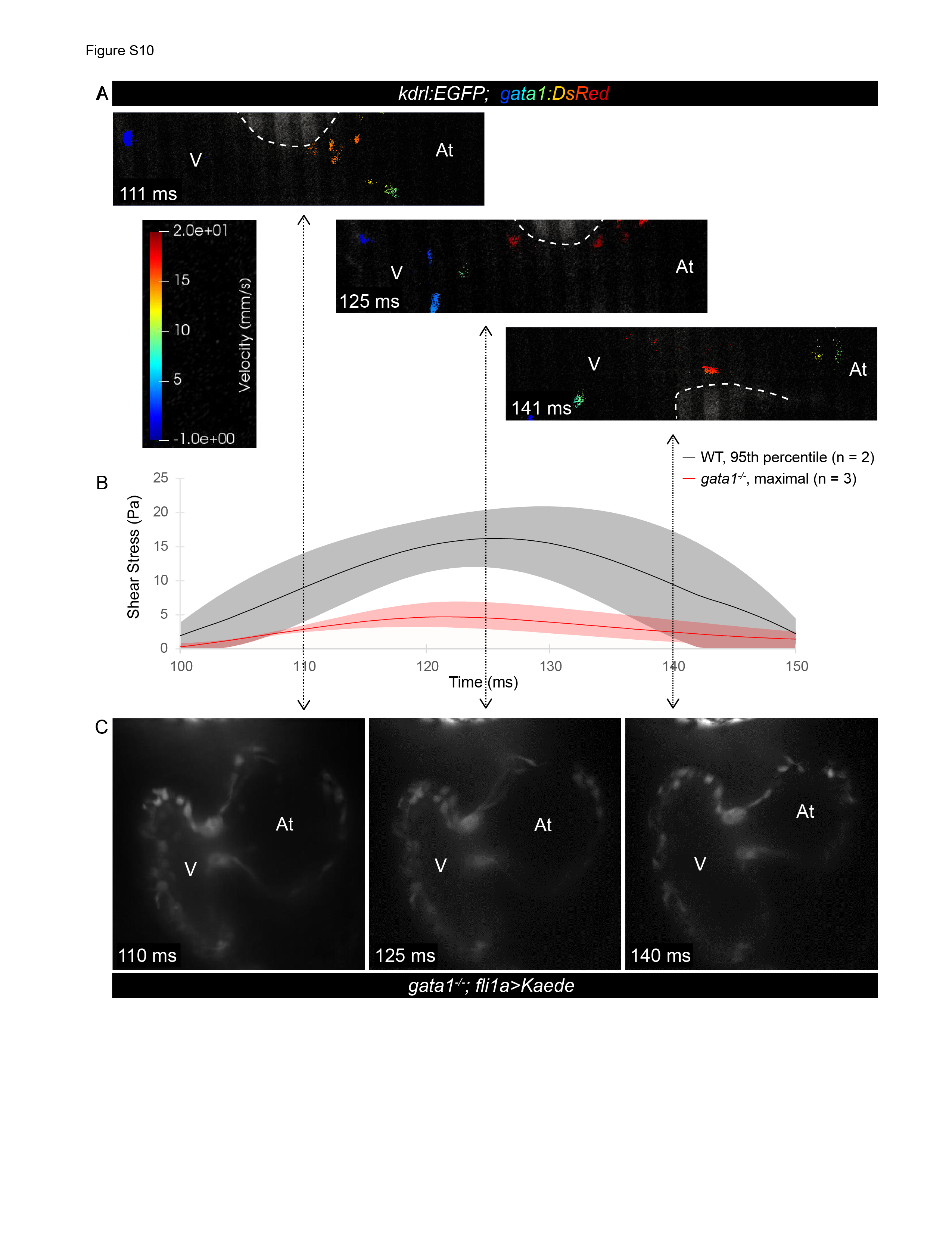

Supplement: S10 Fig — (A) Images of a 65 hpf wild-type embryo in the Tg(kdrl:EGFP; gata1:DsRed) background used for shear stress modeling. Red blood cells express DsRed and are tracked automatically. Segmented red blood cells are pseudo-colored according to their velocity. Dotted lines show the location of the AVC as observed using the EGFP signal. (B) Graph showing the modeled shear stress for wild-type and gata1 mutants. Curves for embryos are aligned such that the AVC lumen is the widest at 125 ms. Centreline shows mean, shaded error shows the range of values. The 95th percentile for shear stress of wild type is calculated and smoothed with an envelope of ±2 time frames. (C) Examples of images taken of a gata1 mutant in the Tg(fli1a;gal4ff;UAS:Kaede) background used for shear stress modeling. Values used to plot the graph can be found in S1 Data. At, atrium; AVC, atrioventricular canal; hpf, hours postfertilization; V, ventricle. (TIF) [file pbio.3001505.s010.tif]

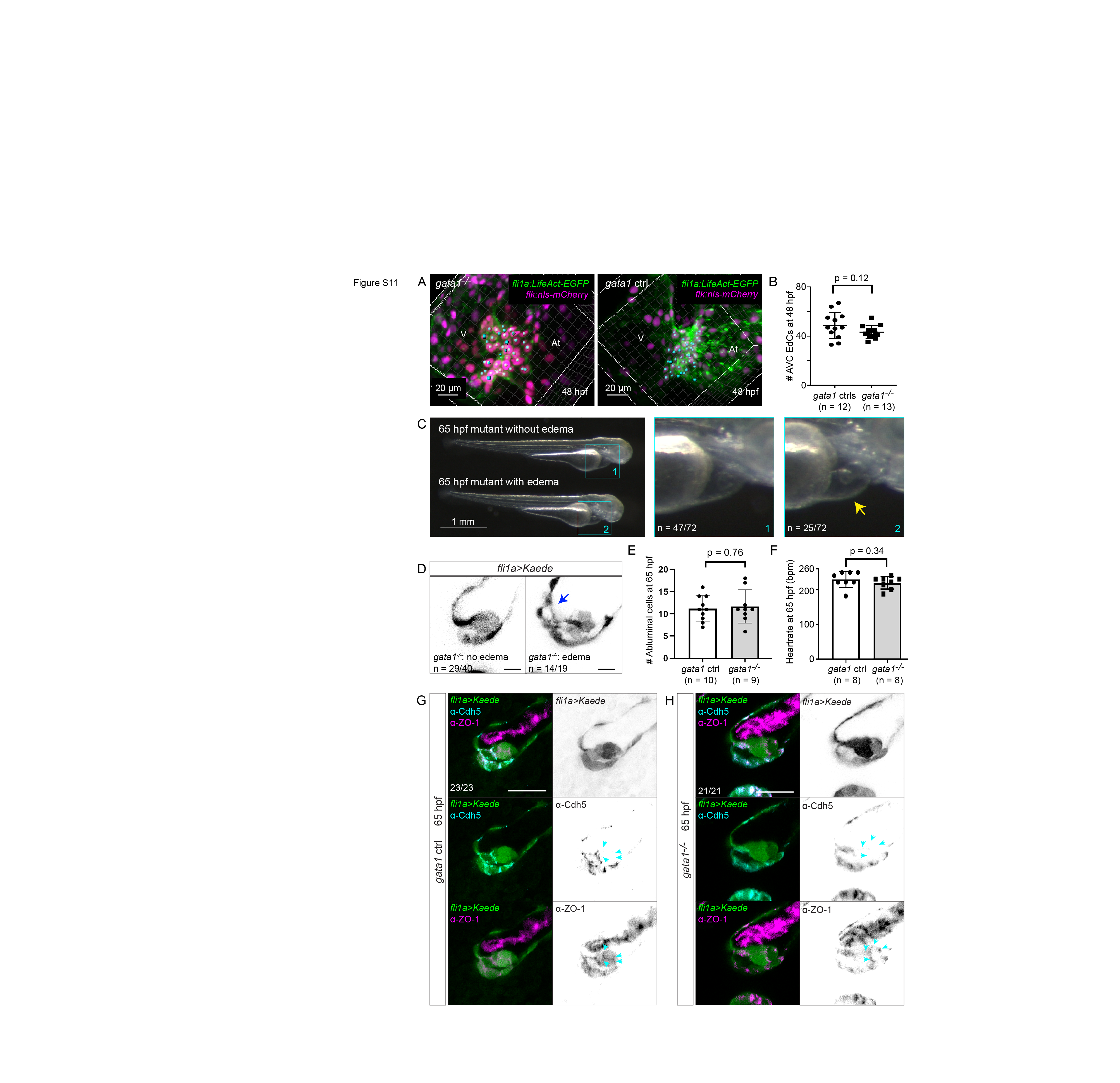

Supplement: S11 Fig — (A) Representative 3D views of the heart in a gata1 control embryo and a gata1 mutant embryo at 48 hpf. Cyan dots are markers of endocardial cell nuclei in the AVC as determined semiautomatically using Imaris software. (B) Dot plot showing the number of endocardial cells in the AVC at 48 hpf. (C) Representative images of 65 hpf gata1 mutants with and without pericardial edema. Middle and right panels show the zoomed in images of boxed regions in the left panel. Yellow arrow points to the region where pericardial edema is most obvious. (D) Representative images of AV valves in 65 hpf gata1 mutants with and without pericardial edema. Blue arrow points to an aberrant connection between abluminal cell structure and luminal AVC cells. (E) Graph showing number of abluminal cells in gata1 controls and mutants at 65 hpf. (F) Graph showing heartrate of gata1 controls and mutants at 65 hpf as calculated from movies of the beating heart taken at 100 frames per second. Statistical significance was calculated using Student t test. (J, K) Gata1 controls (G) and mutants (H) immunostained for VE-cadherin (Cdh5) and ZO-1 at 65 hpf. All embryos had abluminal cells that down-regulated VE-cadherin but were immunopositive for ZO-1. Cyan arrows indicate cell–cell interfaces where VE-cadherin is down-regulated and ZO-1 signal is present. Scale bars: 20 μm. The data underlying all the graphs can be found in S1 Data. AV, atrioventricular valve; AVC, atrioventricular canal; hpf, hours postfertilization; ZO-1, zonula occludens-1. (TIF) [file pbio.3001505.s011.tif]

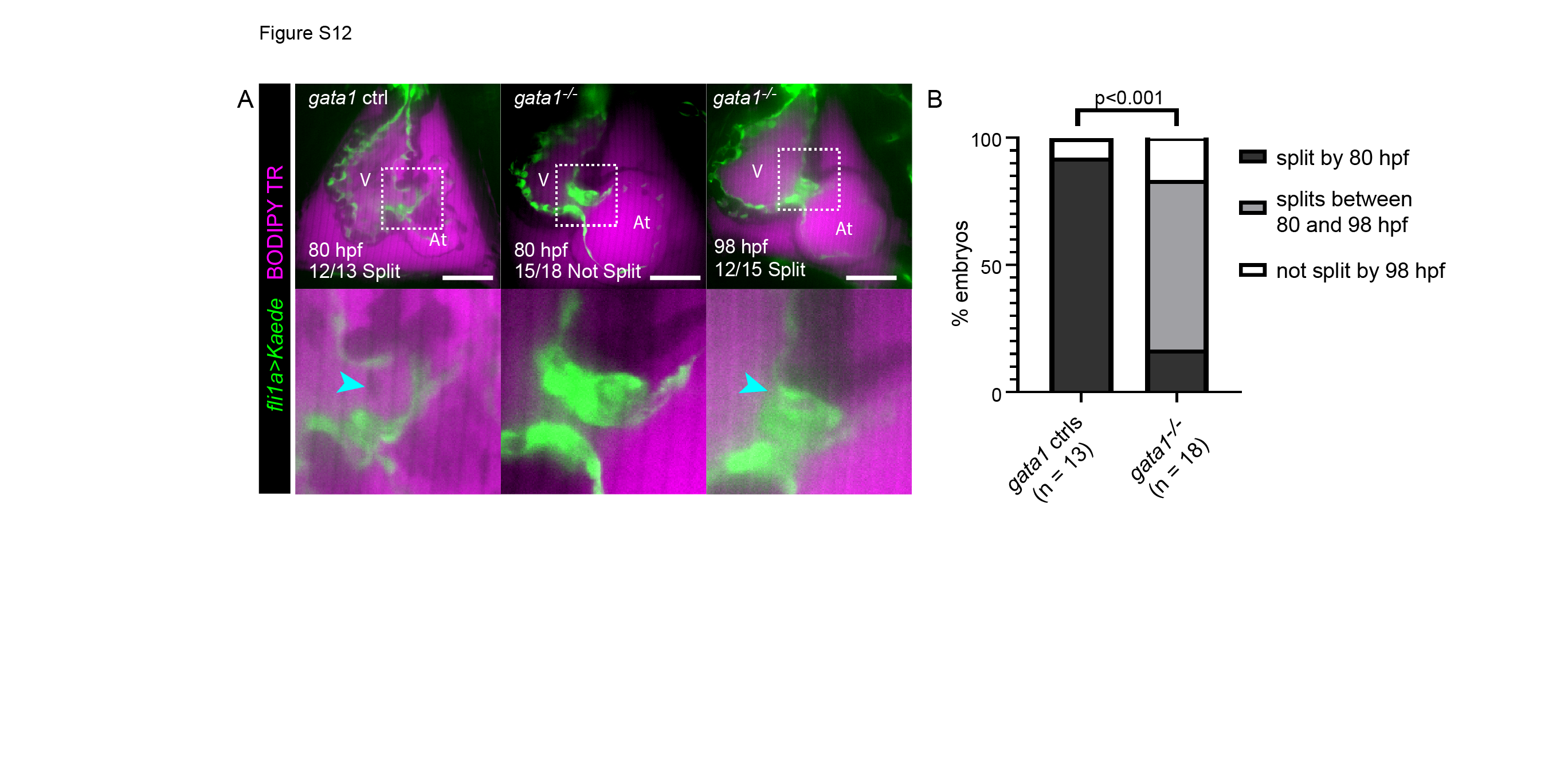

Supplement: S12 Fig — (A) Gata1 controls and gata1 mutants in the Tg(fli1a:gal4ff;UAS:Kaede) were stained with BODIPY TR Ceramide and imaged using the spinning disk at 100 frames per second at 80 hpf. Embryos with valves that have not delaminated at 80 hpf were unmounted and returned to the incubator to develop normally until 98 hpf when they were imaged again. Left column shows a gata1 control valve that has delaminated at 80 hpf. Middle column shows a gata1 mutant that has failed to delaminate at 80 hpf. Right column shows the same gata1 mutant at 98 hpf, where the valve has delaminated. Top row shows the entire heart, bottom row shows the enlarged image of the boxed region. Cyan arrowheads indicate the gap between the valve and the AVC wall. Scale bars: 50 μm. (B) Graph showing percentage of valves that have split by 80 hpf, percentage of valves that have not split at 80 hpf but have split by 98 hpf, and valves that have not split by 98 hpf. The difference between the percentage of valves split at 80 hpf versus valves not split at 80 hpf was shown to be statistically significant using Fisher exact test. The data underlying the graph can be found in S1 Data. AVC, atrioventricular canal; hpf, hours postfertilization. (TIF) [file pbio.3001505.s012.tif]

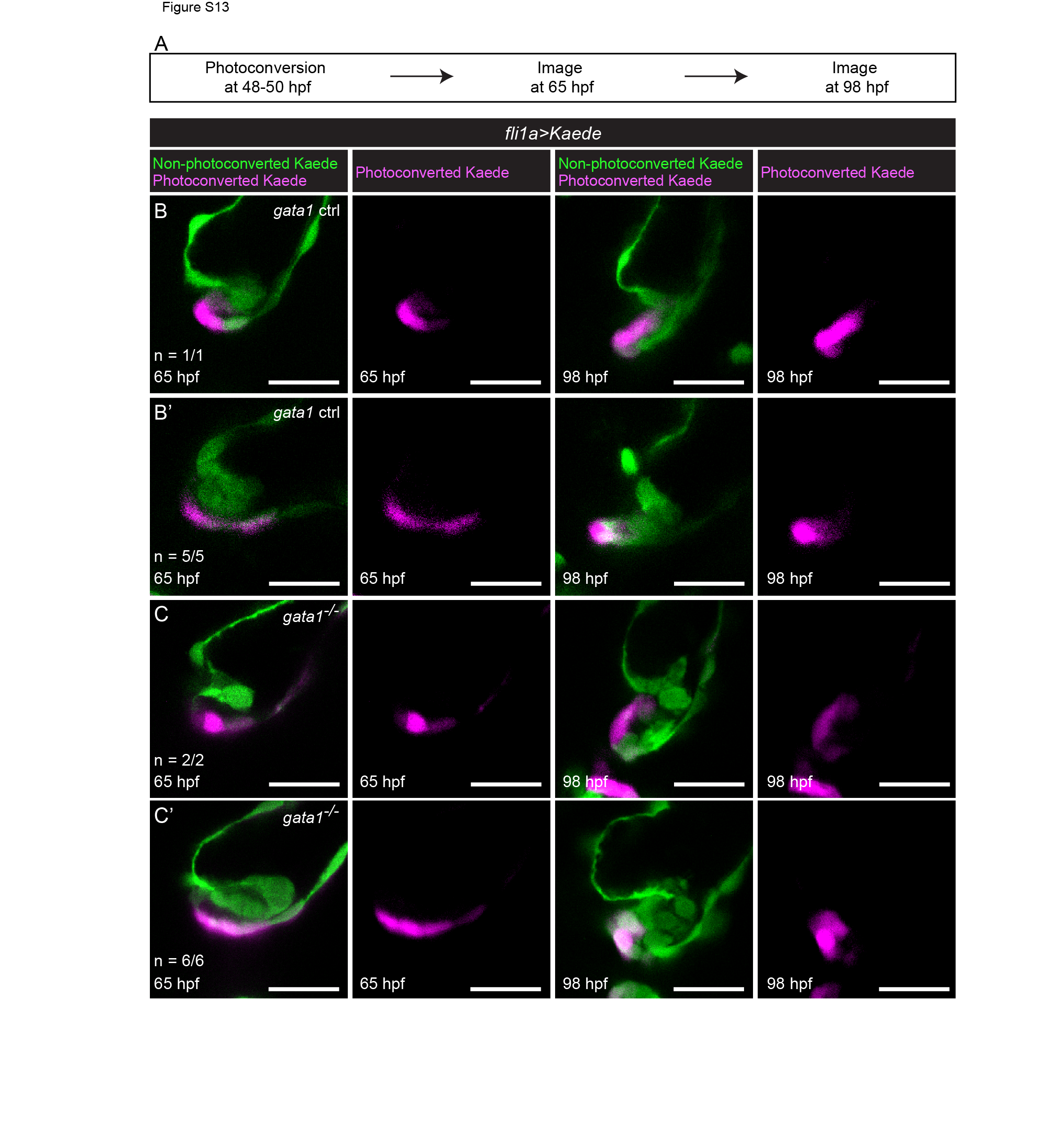

Supplement: S13 Fig — (A) Flow diagram summarizing the method used in this set of photoconversion experiments. At 48 to 50 hpf, embryonic hearts were stopped using BDM and atrial side of the AVC was photoconverted. The embryos were then returned to normal media and allowed to grow normally until 65 hpf, when the heart was stopped again using BDM and the valve imaged (first and second columns in (B–C’)). They were then returned to normal media and allowed to grow normally until 98 hpf, when the heart was stopped using BDM and they were imaged again (third and fourth columns in (B–C’)). (B–B’) Representative images of gata1 controls showing that photoconverting the inferior layer of the abluminal bilayer or luminal endocardial cells of the AVC at 65 hpf does not result in photoconverted cells located abluminally at 98 hpf. (C–C’) Representative images of gata1 mutants. Like gata1 controls, photoconverting luminal endocardial cells of the AVC at 65 hpf does not result in photoconverted cells located abluminally at 98 hpf. AVC, atrioventricular canal; BDM, 2,3-butanedione monoxime; EndoMT, endothelial–mesenchymal transition; hpf, hours postfertilization. (TIF) [file pbio.3001505.s013.tif]

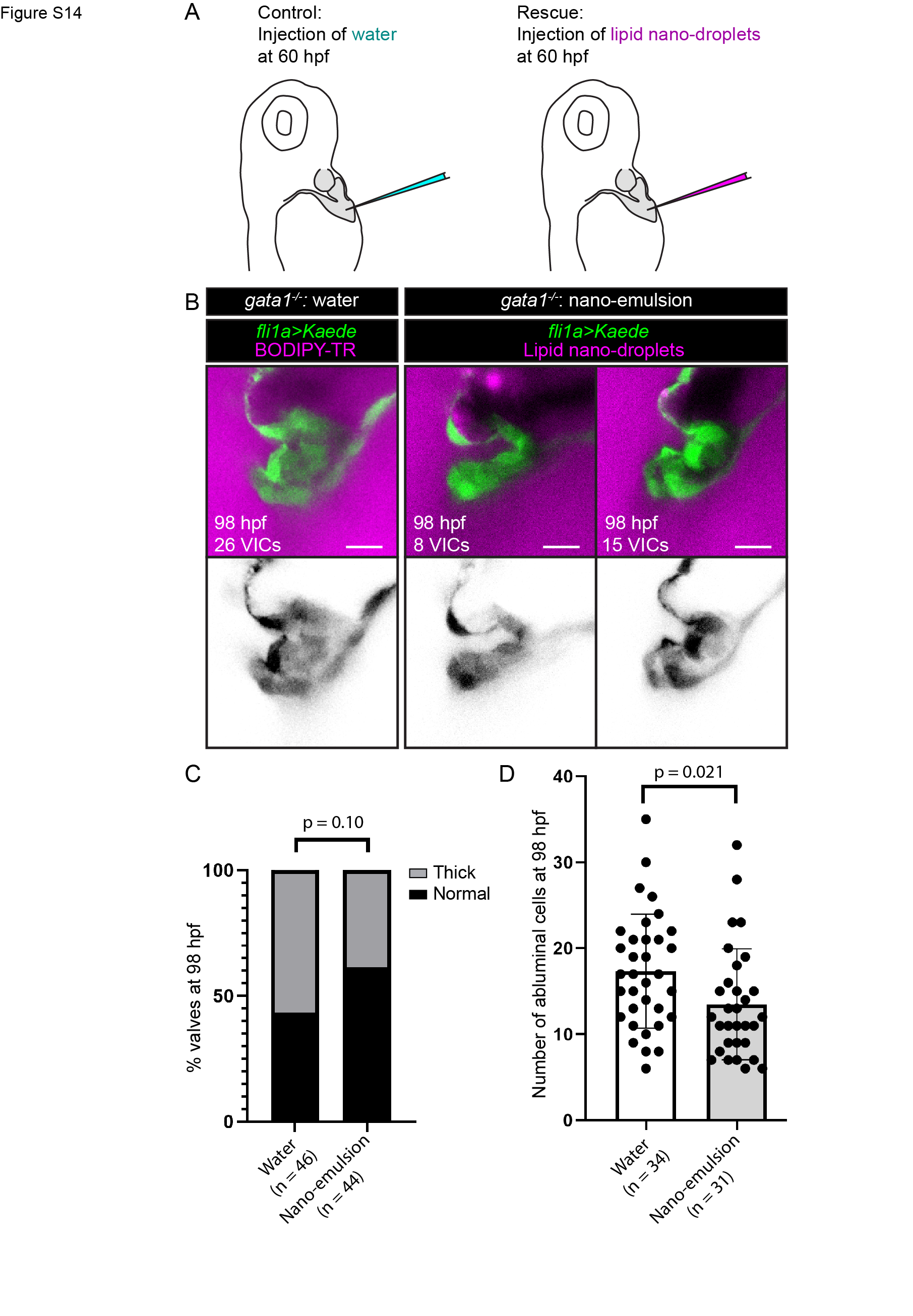

Supplement: S14 Fig — (A) Schematic showing how we injected water or a solution containing lipid nanodroplets into the bloodstream. (B) Examples of 98 hpf gata1 mutant valves injected with water (left panel) or lipid nanodroplets (middle and right panels). The number of VICs counted in each valve is shown. (C) Graph showing that the number of valves with normal thickness was not statistically different between fish that were injected with water and those that were injected with lipid nanodroplets. p-Values are based on Fisher exact test. (D) Graph showing that injecting gata1 embryos with lipid nanodroplets results in fewer abluminal cells at 98 hpf. p-Values are based on the student t test. Scale bars: 10 μm. The data underlying both graphs can be found in S1 Data. hpf, hours postfertilization; VIC, valve interstitial cell. (TIF) [file pbio.3001505.s014.tif]

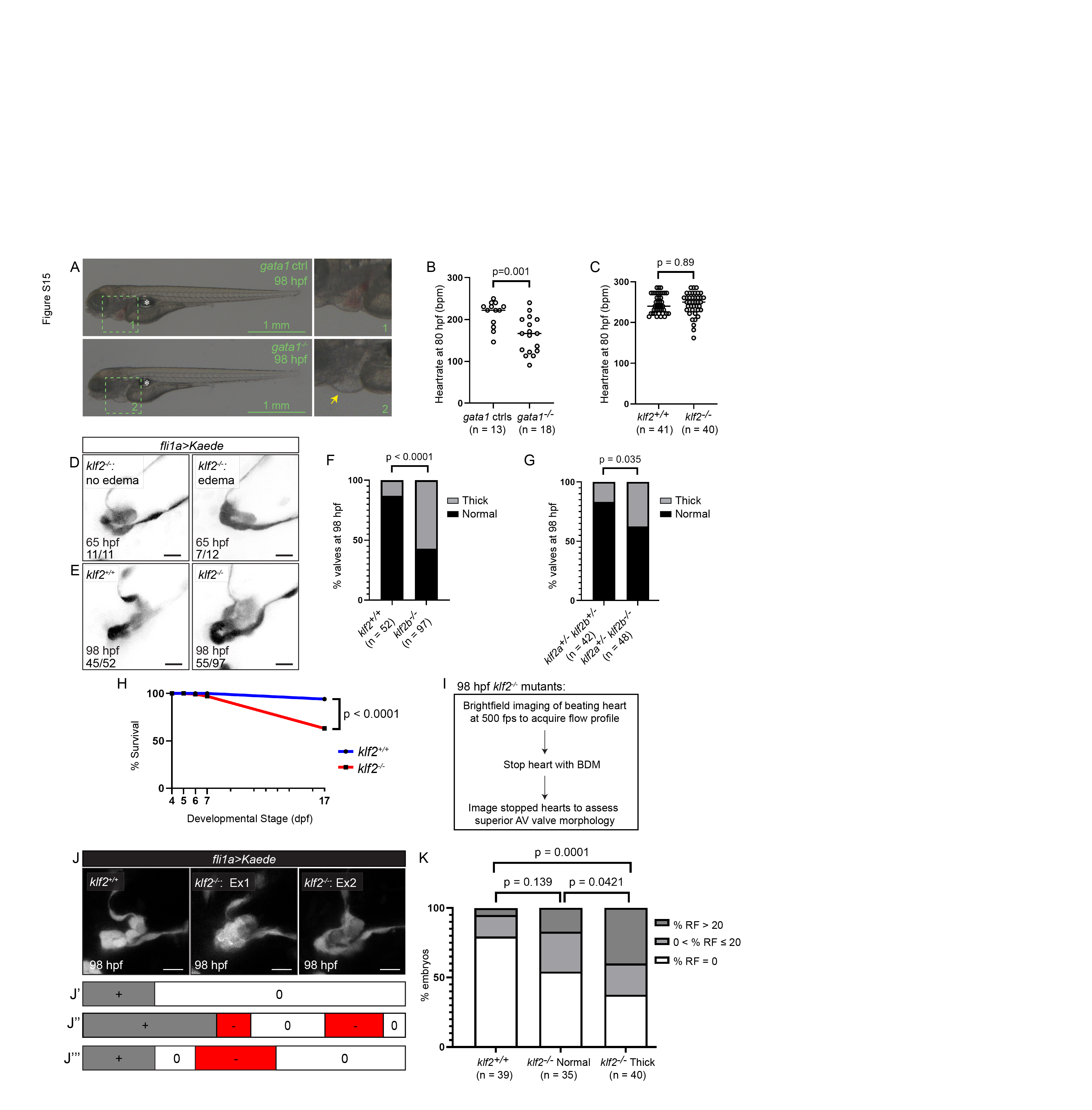

Supplement: S15 Fig — (A) Images showing stereotypical gata1 control and mutant fish with and without pericardial edema at 98 hpf, respectively. White asterisks highlight the swim bladder, which is underdeveloped in gata1 mutants. Images on the right are zoomed in images corresponding to the boxed regions. Yellow arrow points to where pericardial edema is evident. (B) Dot plot showing heartrate of gata1 mutants and controls at 80 hpf. (C) Dot plot showing heartrate of klf2 mutants and controls at 80 hpf. (D, E) Images of klf2 mutants and controls in the Tg(fli1a:gal4ff;UAS:Kaede) background. (D) Representative images of klf2 mutant valves with and without pericardial edema at 65 hpf. Note that in the mutant with edema, there is only 1 layer of cells within the CJ. Embryos are screened between 65 and 80 hpf and those with pericardial edema are excluded from analyses shown in (E, F, and G). (E) Representative images of klf2 control and mutant valves at 98 hpf. (F, G) Graph showing the percentage of valves at 98 hpf with thick or normal morphology in klf2 controls and mutants (F), and in klf2a+/− klf2b+/− embryos and klf2a+/− klf2b−/− embryos (G). p-Values were calculated using Fisher exact test. (H) Klf2 controls and mutants without pericardial edema at 80 hpf were used for a survival study. By 17 dpf, a significantly greater number of klf2 mutant larvae have died based on the Gehan–Breslow–Wilcoxon statistical test. (I) Method used for assessing flow profiles of klf2 mutants with normal or thick valves. (J) Example of a klf2 control valve and 2 examples of klf2 mutant superior AV valves at 98 hpf. Scale bars: 10 μm. (J’–J”’) Flow profiles across the AVC for the valves shown in left, middle, and right panels of (J), respectively. The ends of the rectangles corresponding to the start of atrial systole. White, gray, and red regions show the fraction of the cardiac cycle corresponding to no flow, forward flow, and RF, respectively. (K) The percentage of the cardiac cycle showing RF across the [file pbio.3001505.s015.tif]

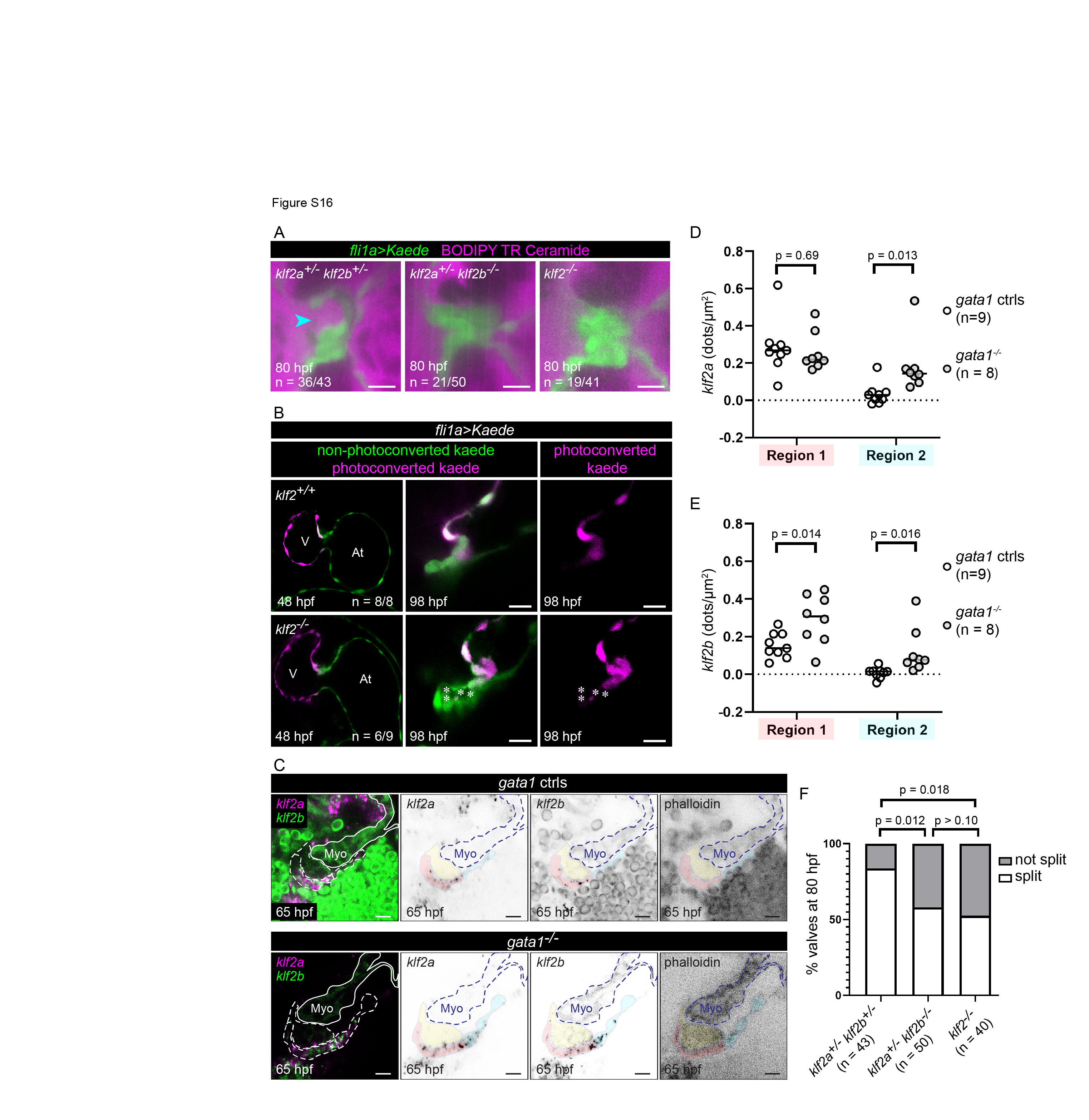

Supplement: S16 Fig — (A) Selected time frames of movies of the beating heart of 80 hpf klf2a+/− klf2b+/−, klf2a+/− klf2b−/− and klf2−/− mutants showing when the valve appears the least compressed. Cyan arrowhead indicates the gap between the inner layer of the valve leaflet and the AVC wall. Scale bar: 10 μm. (B) Representative images of klf2+/+ controls and klf2−/− mutants where the ventricle and the ventricular edge of the AVC were photoconverted at 48 hpf (left column) and imaged again at 98 hpf (middle and right columns). In 8/8 klf2+/+ controls, all abluminal cells were photoconverted at 98 hpf. In 6/9 klf2−/− mutants, some abluminal cells were not photoconverted (white asterisks). Scale bars: 10 μm. (C) Representative images of gata1 mutant and control valves at 65 hpf that have been stained using klf2a and klf2b probes. (D,E) are dot plots showing the number of detected dots, each corresponding to 1 klf2a (D) or 1 klf2b (E) mRNA molecule, calculated for luminal endocardial cells of 2 different regions. Region 1 corresponds to the region shaded in pink in (C), while region 2 corresponds to the region shaded in light blue in (C). p-Values were determined using Student t test. Scale bars: 10 μm. (F) Graph showing percentage of klf2a+/− klf2b+/−, klf2a+/−, klf2b−/−, and klf2−/− mutant valves at 80 hpf that have delaminated, as determined from movies such as that shown in (A). Statistical significance was determined using Fisher exact test. The data underlying all the graphs can be found in S1 Data. At, atrium; AVC, atrioventricular canal; hpf, hours postfertilization; V, ventricle. (TIF) [file pbio.3001505.s016.tif]

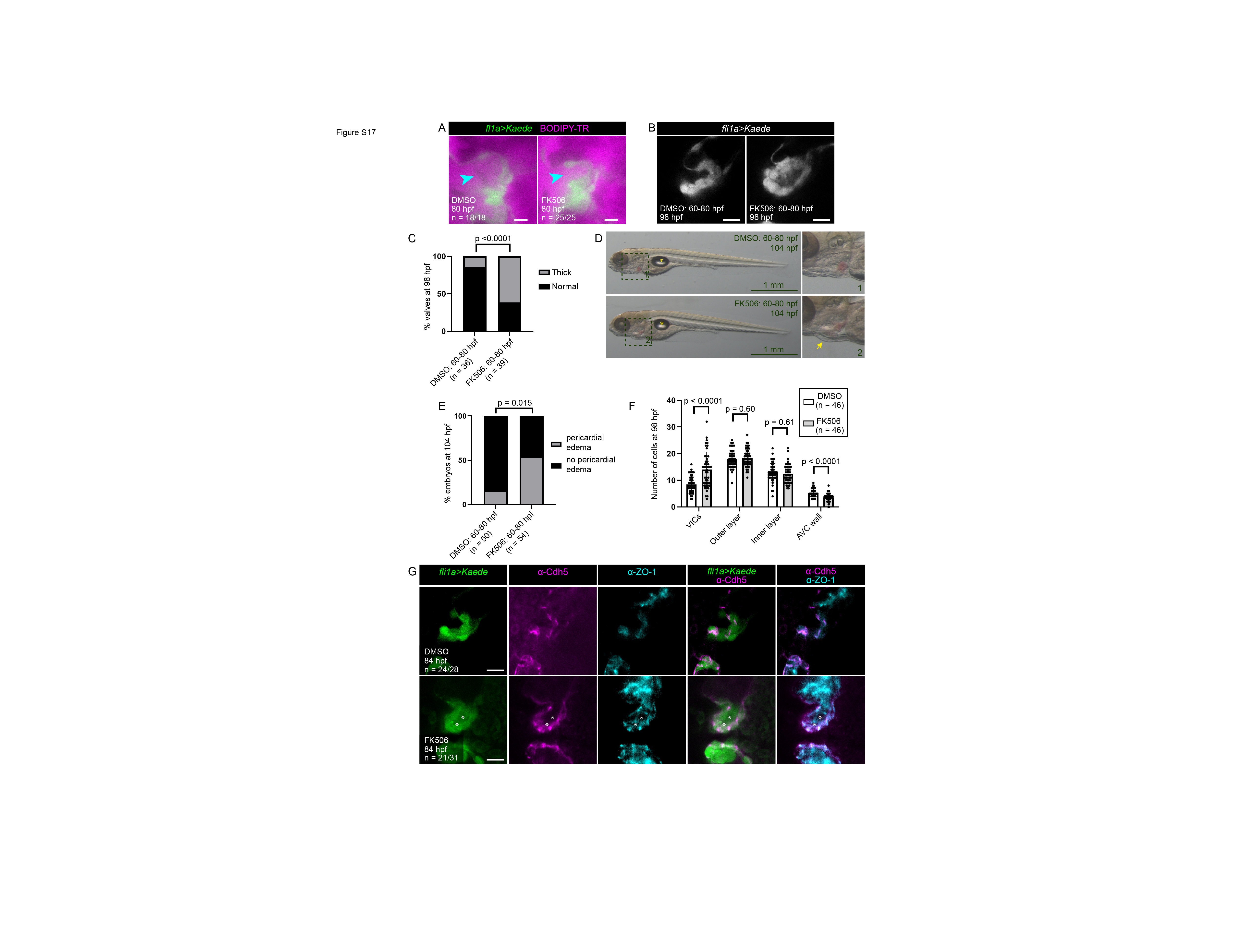

Supplement: S17 Fig — (A) Representative images of 80 hpf superior AV valves imaged in the beating heart of embryos treated with DMSO or FK506 from 60 to 80 hpf. Cyan arrowheads point to the gap between the AVC wall and the inner layer of the valve leaflet. Scale bars: 10 μm. (B) Representative images of 98 hpf superior AV valves imaged in embryos treated with DMSO or FK506 from 60 to 80 hpf. Scale bars: 10 μm. (C) Graph showing percentage of normal and thick valves in embryos treated with DMSO or FK506 from 60 to 80 hpf. p-Values were calculated using Fischer exact test. (D) Representative images of 104 hpf embryos that have been treated with DMSO or FK506 from 60 to 80 hpf. Right-side images are zoomed in images of the boxed regions. Yellow arrow points toward where pericardial edema is most obvious in the FK506-treated embryo. Yellow asterisks indicate the swim bladder, which is smaller or absent in FK506-treated embryos. (E) Graph showing percentage of embryos in (D) that have pericardial edema. p-Values were calculated using Fisher exact test. (F) Graph showing cell numbers by valve region at 98 hpf for embryos treated with DMSO or FK506 from 60 hpf. Statistical significance was determined by multiple t tests. (G) Representative images of embryos that have been treated with DMSO or FK506 from 60 hpf, fixed at 84 hpf, then immunostained for Cdh5 and ZO-1. White asterisks mark additional abluminal cells in FK506-treated embryos that express ZO-1 but not Cdh5. Scale bars: 10 μm. The data underlying all the graphs can be found in S1 Data. AVC, atrioventricular valve; hpf, hours postfertilization; ZO-1, zonula occludens-1. (TIF) [file pbio.3001505.s017.tif]
